# Stereoselective Multigram-Scale Tn Antigen Synthesis via the Iron-Catalyzed Glycal 1,2-*cis*-Aminoglycosylation

**DOI:** 10.1021/acs.orglett.5c01560

**Published:** 2025-05-16

**Authors:** Le Yin, Dakang Zhang, Zixiang Jiang, Hao Xu

**Affiliations:** Department of Chemistry, 8244 Brandeis University, 415 South Street, Waltham, Massachusetts 02453, United States

## Abstract

We report here a new catalytic and exclusively *cis*-selective glycosylation strategy for multigram scale synthesis of biologically valuable Tn antigens. The underlying iron-catalyzed glycal 1,2-*cis*-aminoglycosylation method is effective with a variety of galactosyl donors and amino acid acceptors with consistently high stereoselectivity. Rapid and scalable postglycosylation transformations readily afford single diastereomeric Tn antigens in high yields.

## Graphical abstract

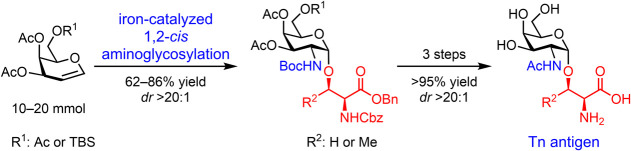

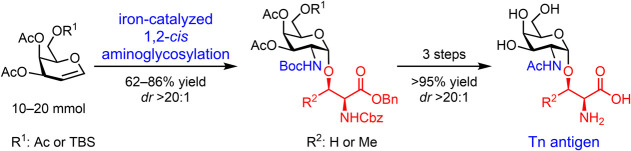

Glycosylation is an important protein post-translational modification that plays a key role in regulating protein structures and functions.^1^ Unlike *N*-linked glycosylation^2,3^ that happens cotranslationally on a specific consensus sequence, *O*-linked glycosylation^4^ occurs after translation on serine and threonine residues that do not seem to belong to any consensus sequence. Therefore, chemical and chemo-enzymatic synthesis is the most important approach for providing *O*-linked glycopeptides and glycoproteins in high homogeneity, which is indispensable for studying the biology of protein *O*-linked glycosylation.^5,6^

The most prevalent *O*-linked glycosylation in eukaryotes is mucin-type glycosylation,^7^ in which *N*-acetylgalactosamine (GalNAc) is directly connected to a serine or threonine residue of the target protein through an α-glycosidic linkage. These structures serve as the foundational scaffolds for further enzymatic elaboration to generate diverse glycan structures. As a result, incorporation of a single diastereomeric *O*-galactosyl amino acid (Tn antigen (**1**) in Figure 1) in peptide synthesis followed by enzymatic glycan extension has become the standard procedure for the synthesis of *O*-linked glycopeptides and glycoproteins.^8–11^

**Figure 1:**
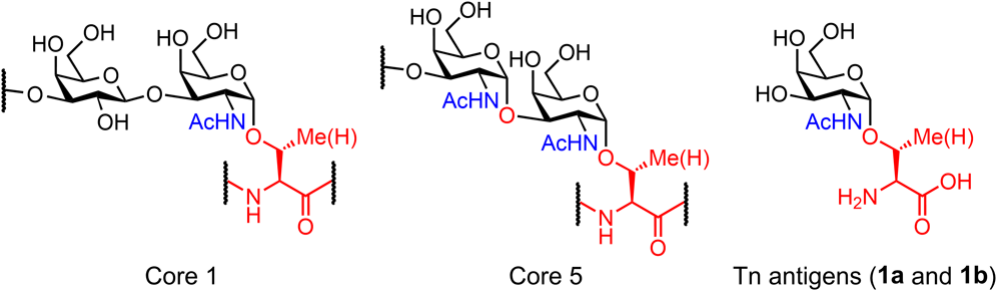
Representative mucin-type *O*-linked glycoprotein core structures and Tn antigen structure.

The synthesis of single diastereomeric Tn antigens is not trivial because the embedded 1,2-*cis*-α-amidoglycosidic linkages are difficult to form reliably in high stereoselectivity.^12^ Over the past several decades, numerous elegant glycosylation methods were developed and an array of glycosyl donors were invented that minimize neighboring group participation and thus enhance the 1,2-*cis*-α-selectivity.^12–26^ Some of these valuable methods can provide stereochemically pure Tn antigens,^16,27–31^ but they are most effective with specific substrates: structural variations either in galactosyl donors or amino acid acceptors often compromise the high 1,2-*cis*-selectivity and afford a diastereomeric mixture.^16,26,28–42^ This has been particularly problematic for serine acceptors,^26,29,39^ which may contribute to the hefty price of Tn antigen (**1a**) ($417/mg from Millipore–Sigma). Notably, there has not been an exclusively *cis*-selective glycosylation strategy for Tn antigen synthesis that is also broadly effective with various substrates. We report herein a generally applicable glycosylation strategy for multigram-scale synthesis of single diastereomeric Tn antigens via the iron-catalyzed glycal 1,2-*cis*-aminoglycosylation.

We have developed an iron-catalyzed glycal aminoglycosylation method for 1,2-*cis*-aminoglycoside synthesis.^43^ This exclusively *cis*-selective method is effective with a broad range of glycosyl donors and acceptors (eq 1 in Scheme 1). Electron-rich glycals are the most reactive substrates and they are connected with primary acceptors to afford 1,2-*cis*-aminoglycosides using a variety of amination reagents (such as **3a** and **3b**). However, sterically more hindered **3b** is necessary for glycosylation of these donors with secondary acceptors to minimize the competing *cis*-aminoacyloxylation.^44^ Interestingly, although tri-*O*-acyl-d-glucals are not suitable glycosyl donors, electron-deficient glycals with at least a 3-*O*-silyl group are excellent substrates. For these donors, glycosylation with **3a** is effective for both primary and secondary acceptors. We also discovered that this glycosylation method is compatible with amino acid–based acceptors. Aminoglycosylation of 4,6-di-*O*-acetyl-3-*O*-TBS-d-galactal (**2**) with *N*-Cbz-protected serine and threonine methyl esters **4a** and **4b**, afforded fully protected Tn antigens **5a** and **5b** in good yields (73–84% yield, *dr* > 20:1, eq 2 in Scheme 1).^43^ Notably, amination reagent **3b** proves more effective for glycosylation of **2** with **4b**.

**Scheme 1:**
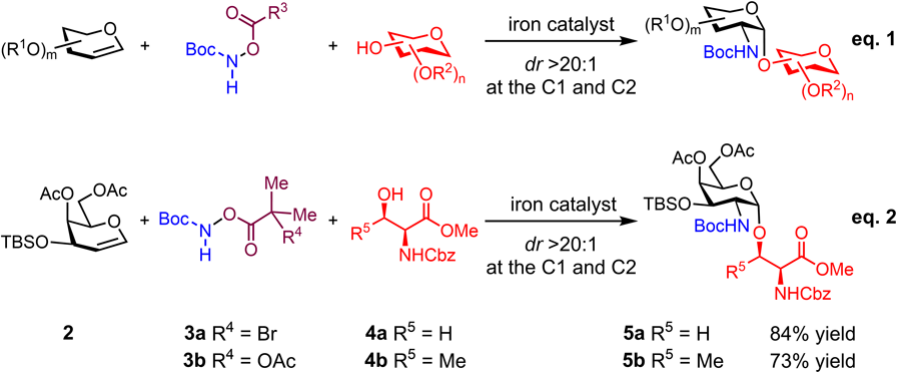
Iron-Catalyzed Glycal 1,2-*cis*-Aminoglycosylation and Its Applications in *O*-Galactosyl Amino Acid Synthesis

During scaling up the glycosylation and Tn antigen synthesis, we identified two opportunities to further optimize this synthetic process. First, **2** is prepared from tri-*O*-acetyl-d-galactal, which entails two steps that need chromatographic purifications: one of them involves an enzymatic reaction (using Amano Lipase from *Pseudomonas fluorescens*)^43^ with an extended reaction time (Figure S1). Next, protecting group adjustment on **5a**/**5b** for Tn antigen synthesis requires lengthy reaction times (*N*-Boc→*N*-Ac and OTBS→OAc) with aqueous workups (Figure S2).

To address these two issues, we aimed to identify new galactosyl donors that are both readily available and amenable to rapid postglycosylation deprotection. Entirely by serendipity, we discovered that 3,4-di-*O*-acetyl-6-*O*-TBS-D-galactal (**7**) is an excellent galactosyl donor in the iron-catalyzed glycal *cis*-aminoglycosylation with *N*-Cbz-protected serine **4a** and threonine **4b** in the presence of catalyst **6** ^45^ (68% and 60% yield, *dr* > 20:1, eq 3 and eq 4 in Scheme 2). This bench-stable iron catalyst can be easily prepared from Fe­(BF_4_)_2_(H_2_O)_6_ and tridentate ligand **L1** in the presence of molecular sieves. Interestingly, **7** exhibits even higher reactivity than **2**, which requires fine-tuning of the glycosylation parameters. Reaction optimization revealed that use of 1,4-dioxane as the cosolvent (CH_2_Cl_2_/1,4-dioxane: 9:1) increased the glycosylation yield (86% and 72% yield, *dr* > 20:1, Scheme 2) while minimizing the competing glycal *cis*-aminoacyloxylation.^44^ Notably, replacement of 1,4-dioxane with THF, DME, or diethyl ether shut down the glycosylation (Figure S3). It is also worth noting that **7** can be prepared from d-galactal using a one-pot procedure (Figure S4) and that both glycosylation reactions can be readily scaled up to a 20 mmol-scale. Our mechanistic studies suggested that the iron catalyst activates a serine or threonine glycosyl acceptor and amination reagent **3** when it facilitates the cooperative atom transfer of both moieties to a glycosyl donor in an exclusively *cis*-selective manner (Figure S5).^43,46^ Notably, both a 2-amidoglycosyl radical^47,48^ and a 2-amidoglycosyl oxocarbenium ion are plausible intermediates.

**Scheme 2:**
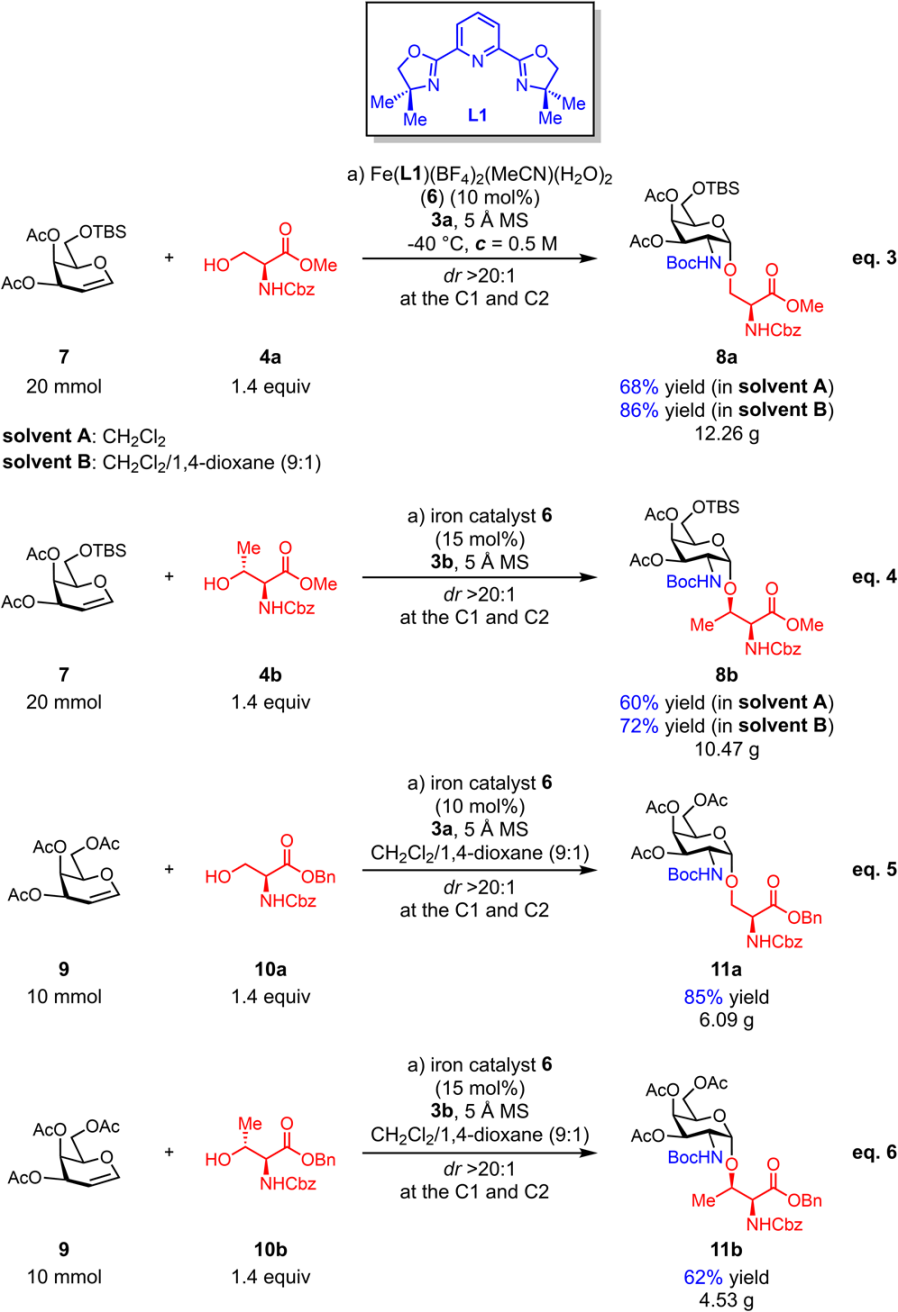
Engaging Challenging Galactosyl Donors in the Iron-Catalyzed Glycal 1,2-*cis*-Aminoglycosylation^a^

a Reactions were carried out with iron catalyst **6** (10–15 mol %) and amination reagent **3a**/**3b** (1.5 equiv) in CH_2_Cl_2_ or CH_2_Cl_2_/1,4-dioxane in the presence of 5 Å MS at −40 °C for 2–3 h. See Supporting Information for details.

To further streamline the Tn antigen synthesis, we explored directly applying commercially available tri-*O*-acetyl-d-galactal (**9**) as the donor while using *N*-Cbz serine and threonine benzyl esters **10a** and **10b** as the acceptors (eq 5 and eq 6 in Scheme 2). Surprisingly, unlike tri-*O*-acetyl-d-glucal, which is completely unreactive in the iron-catalyzed glycal *cis*-aminoglycosylation,^43^ **9** is a decent substrate: its glycosylation with serine benzyl ester **10a** afforded **11a** in high yield (85% on a 10 mmol scale, *dr* > 20:1, Scheme 2), even though a less-efficient glycosylation was observed with threonine acceptor **10b** (62% yield for **11b** on a 10 mmol scale, *dr* > 20:1, Scheme 2).

Based upon these discoveries, we subsequently evaluated a range of galactosyl donors and amino acid acceptors to explore the generality of this method (Figure 2). In addition to *N*-Cbz serine and threonine methyl esters, 3,4-di-*O*-acetyl-6-*O*-TBS-d-galactal (**7**) can readily glycosylate *N*-Cbz serine and threonine benzyl and *tert*-butyl esters, affording products **12**–**13** in good yields (64–75%). Furthermore, *N*-Boc serine and threonine methyl esters are also excellent substrates, generating the corresponding products **14a** and **14b** in high yields. Notably, glycosylation with *N*-Fmoc serine and threonine acceptors did lead to single diastereomeric products, albeit in low yields with low conversions, presumably due to the poor solubility of *N*-Fmoc amino acids under cryogenic conditions (Figure S6). Interestingly, this method is also compatible with glucals, delivering the corresponding glucosyl serine and threonine in high yields with the exclusive 1,2-*cis*-selectivity (Figure S7).

**Figure 2:**
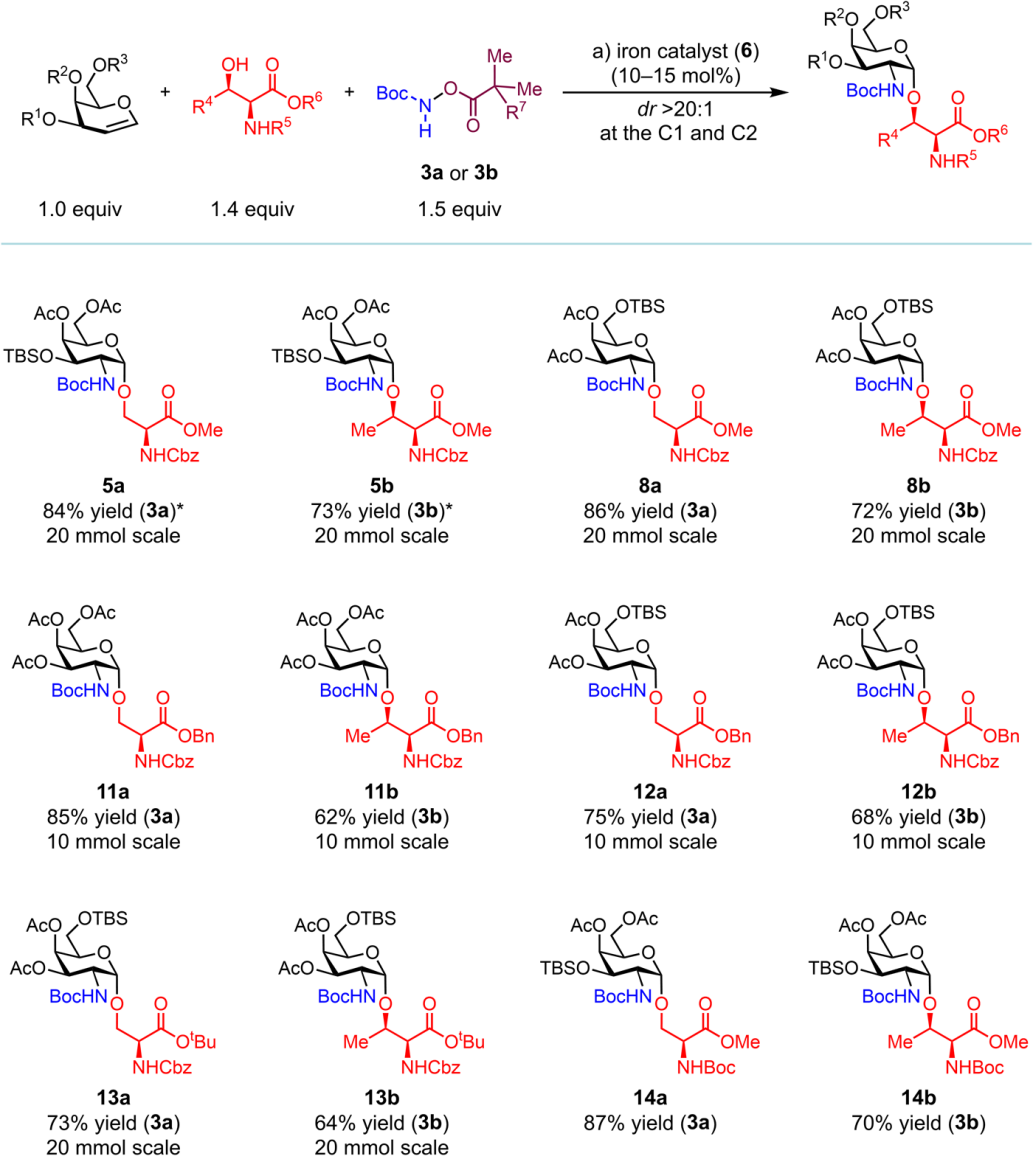
Substrate scope for the *O*-galactosyl amino acid synthesis via the iron-catalyzed 1,2-*cis*-selective glycal aminoglycosylation. All yields are isolated yields. ^*a*^Reactions were carried out with iron catalyst **6** (10–15 mol %) in CH_2_Cl_2_/1,4-dioxane (9:1) in the presence of 5 Å MS at −40 °C for 2–4 h. *These reactions were carried out in CH_2_Cl_2_. See Supporting Information for details.

With single diastereomeric fully protected Tn antigens prepared on multigram scales, we next investigated rapid and scalable deprotection procedures that minimize aqueous workups (Figure 3). Facile *N*-Boc deprotection of **11a** using 4 N HCl in 1,4-dioxane followed by HCl removal *in vacuo* and *N*-acetylation provided the product almost quantitatively (*N*-Boc→*N*-Ac) in 6 h. Next, standard hydrogenolysis at 1 atm afforded **15a** in 2 h (>95% yield). Finally, rapid methanolysis of *O*-acetyl groups in **15a** quantitatively furnished serine Tn antigen **1a** in 30 min. Alternatively, **15a** can be readily converted to *N*-Fmoc-protected serine Tn antigen **16a** in 30 min (94% yield). Similarly, fully protected threonine Tn antigen **12b** can be rapidly converted to either threonine Tn antigen **1b** or *N*-Fmoc-protected threonine Tn antigen **16b** (86–89% yield over three steps). It is worth noting that *N*-Boc and 6-*O*-TBS removal from **12b** is achieved quantitatively in a single step and that facile deprotection procedures were also developed for other fully protected Tn antigens (Figure S10).

**Figure 3:**
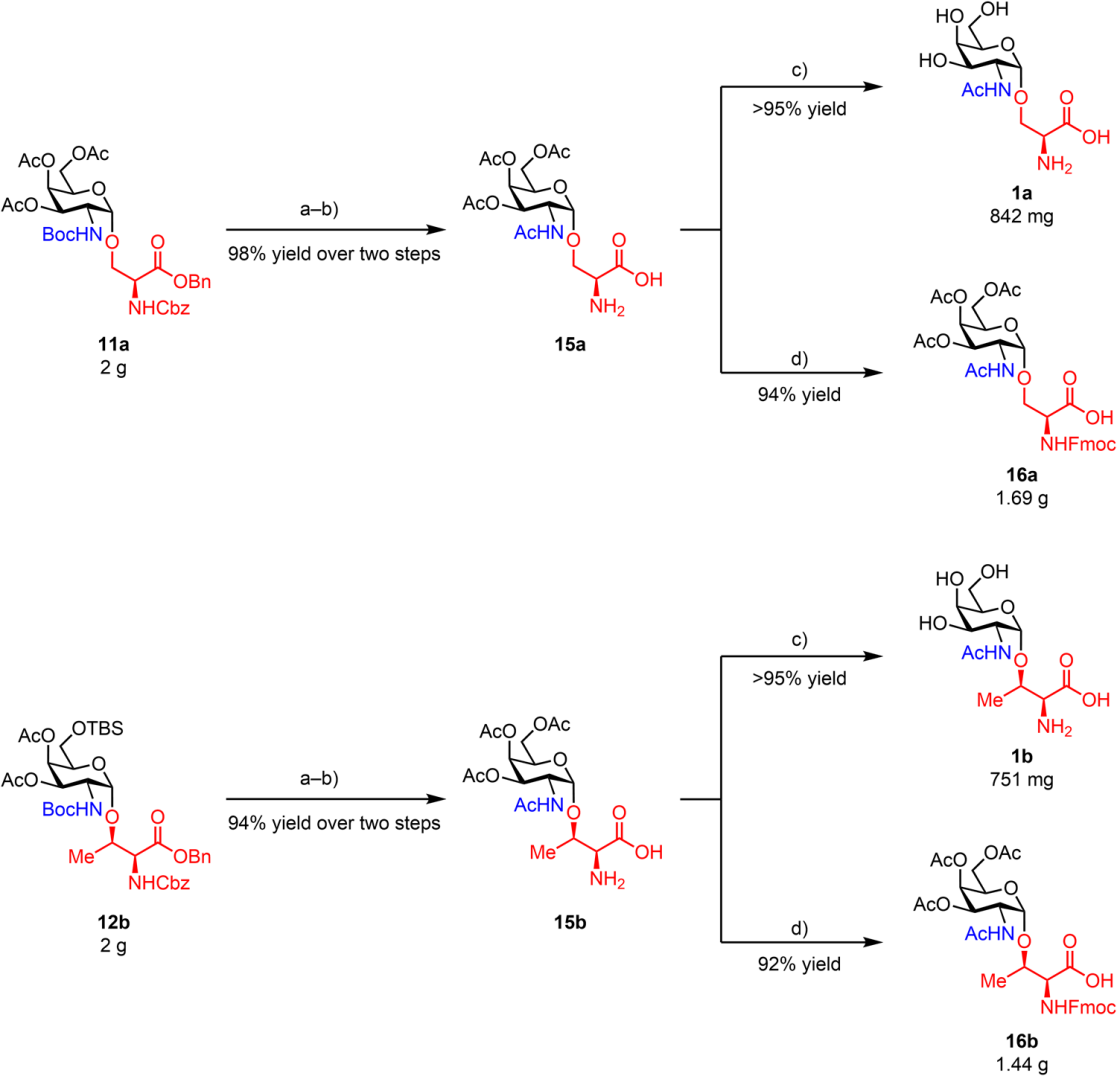
Postglycosylation transformations to afford Tn antigens. ^*a*^4 N HCl in 1,4-dioxane (5.0 equiv), 0 to 22 °C, followed by HCl removal *in vacuo*; then Ac_2_O (2.0 equiv), pyridine (3.0 equiv), CH_2_Cl_2_, 0 to 22 °C. ^*b*^Pd/C (20 wt %), H_2_ (1 atm), MeOH, 22 °C. ^*c*^K_2_CO_3_ (0.5 equiv), MeOH, 0 °C. ^*d*^FmocOSu (1.0 equiv), saturated aqueous NaHCO_3_, MeCN, 0 °C.

In summary, we have developed a highly stereoselective catalytic glycosylation strategy for multigram scale synthesis of biologically valuable Tn antigens. The underlying iron-catalyzed exclusively 1,2-*cis*-selective glycosylation is applicable to a wide variety of galactosyl donors and amino acid acceptors. Rapid and scalable postglycosylation deprotection procedures minimize aqueous workups and readily afford single diastereomeric Tn antigens.

## Supplementary Material



## Data Availability

The data underlying this study are available in the published article and its Supporting Information.

## Notes

Experimental procedure, characterization data for all new compounds and selected NMR spectra. (PDF)

## References

[1] Dwek R. A. (1996). Glycobiology:
Toward Understanding the Function of Sugars. Chem. Rev..

[2] Kornfeld R., Kornfeld S. (1985). Assembly of Asparagine-Linked
Oligosaccharides. Annu. Rev. Biochem..

[3] Li C., Wang L.-X. (2018). Chemoenzymatic Methods
for the Synthesis of Glycoproteins. Chem. Rev..

[4] Steen P. V. d., Rudd P. M., Dwek R. A., Opdenakker G. (1998). Concepts and
Principles of *O*-Linked Glycosylation. Crit. Rev. Biochem. Mol. Biol..

[5] Grogan M. J., Pratt M. R., Marcaurelle L. A., Bertozzi C. R. (2002). Homogeneous Glycopeptides
and Glycoproteins for Biological Investigation. Annu. Rev. Biochem..

[6] Yang W., Ramadan S., Zu Y., Sun M., Huang X., Yu B. (2024). Chemical Synthesis and Functional
Evaluation of Glycopeptides and
Glycoproteins Containing Rare Glycosyl Amino Acid Linkages. Nat. Prod. Rep..

[7] Marcaurelle L. A., Bertozzi C. R. (2002). Recent Advances in the Chemical Synthesis of Mucin-Like
Glycoproteins. Glycobiology.

[8] Pratt M. R., Bertozzi C. R. (2005). Synthetic Glycopeptides
and Glycoproteins as Tools
for Biology. Chem. Soc. Rev..

[9] Malekan H., Fung G., Thon V., Khedri Z., Yu H., Qu J., Li Y., Ding L., Lam K. S., Chen X. (2013). One-pot Multi-enzyme
(OPME) Chemoenzymatic Synthesis of Sialyl-Tn-MUC1 and Sialyl-T-MUC1
Glycopeptides Containing Natural or Non-Natural Sialic Acid. Bioorg. Med. Chem..

[10] Zhang J., Liu D., Saikam V., Gadi M. R., Gibbons C., Fu X., Song H., Yu J., Kondengaden S. M., Wang P. G., Wen L. (2020). Machine-Driven Chemoenzymatic
Synthesis
of Glycopeptide. Angew. Chem., Int. Ed..

[11] Lin P.-H., Xu Y., Bali S. K., Kim J., Gimeno A., Roberts E. T., James D., Almeida N. M. S., Loganathan N., Fan F., Wilson A. K., Jonathan
Amster I., Moremen K. W., Liu J., Jiménez-Barbero J., Huang X. (2024). Solid-Phase-Supported
Chemoenzymatic Synthesis and Analysis of Chondroitin Sulfate Proteoglycan
Glycopeptides. Angew. Chem., Int. Ed..

[12] Nigudkar S. S., Demchenko A. V. (2015). Stereocontrolled
1,2-*cis* Glycosylation
as the Driving Force of Progress in Synthetic Carbohydrate Chemistry. Chem. Sci..

[13] Kaifu R., Osawa T. (1977). Synthesis of *O*-(2-acetamido-2-deoxy-α-D-galactopyranosyl)-*N*-tosyl-L-serine. Carbohydr. Res..

[14] Ratcliffe R. M., Baker D. A., Lemieux R. U. (1981). Synthesis
of the T [β-D-Gal-(1→3)-α-D-GalNAc]-antigenic
Determinant in a Form Useful for the Preparation of an Effective Artificial
Antigen and the Corresponding Immunoadsorbent. Carbohydr. Res..

[15] Kunz H., Birnbach S. (1986). Synthesis of *O*-Glycopeptides
of the
Tumor-Associated Tn- and T-Antigen Type and Their Binding to Bovine
Serum Albumin. Angew. Chem., Int. Ed..

[16] Winterfeld G. A., Schmidt R. R. (2001). Nitroglycal Concatenation:
A Broadly Applicable and
Efficient Approach to the Synthesis of Complex *O*-Glycans. Angew. Chem., Int. Ed..

[17] Benakli K., Zha C., Kerns R. J. (2001). Oxazolidinone
Protected 2-Amino-2-deoxy-D-glucose Derivatives
as Versatile Intermediates in Stereoselective Oligosaccharide Synthesis
and the Formation of α-Linked Glycosides. J. Am. Chem. Soc..

[18] Orgueira H. A., Bartolozzi A., Schell P., Seeberger P. H. (2002). Conformational
Locking of the Glycosyl Acceptor for Stereocontrol in the Key Step
in the Synthesis of Heparin. Angew. Chem., Int.
Ed..

[19] Svarovsky S. A., Barchi J. J. (2003). Highly Efficient
Preparation of Tumor Antigen-Containing
Glycopeptide Building Blocks from Novel Pentenyl Glycosides. Carbohydr. Res..

[20] Manabe S., Ishii K., Ito Y. (2006). *N*-Benzyl-2,3-oxazolidinone
as a Glycosyl Donor for Selective α-Glycosylation and One-Pot
Oligosaccharide Synthesis Involving 1,2-*cis*-Glycosylation. J. Am. Chem. Soc..

[21] Park J., Kawatkar S., Kim J.-H., Boons G.-J. (2007). Stereoselective
Glycosylations of 2-Azido-2-deoxy-glucosides Using Intermediate Sulfonium
Ions. Org. Lett..

[22] Ryan D. A., Gin D. Y. (2008). Ring-Opening of
Aziridine-2-Carboxamides with Carbohydrate
C1-*O*-Nucleophiles. Stereoselective Preparation of
α- and β-*O*-Glycosyl Serine Conjugates. J. Am. Chem. Soc..

[23] Mensah E. A., Nguyen H. M. (2009). Nickel-Catalyzed Stereoselective Formation of α-2-Deoxy-2-Amino
Glycosides. J. Am. Chem. Soc..

[24] Codeé J. D. C., Wang L., Zhang Y., Overkleeft H. S., van der Marel G. A. (2020). Reagent Controlled Glycosylations
for the Assembly
of Well-Defined Pel Oligosaccharides. J. Org.
Chem..

[25] Wang S., Chen C., Gadi M. R., Saikam V., Liu D., Zhu H., Bollag R., Liu K., Chen X., Wang F., Wang P. G., Ling P., Guan W., Li L. (2021). Chemoenzymatic
Modular Assembly of *O*-GalNAc Glycans for Functional
Glycomics. Nat. Commun..

[26] Mensah E. A., Yu F., Nguyen H. M. (2010). Nickel-Catalyzed
Stereoselective Glycosylation with
C(2)*-N*-Substituted Benzylidene D-Glucosamine and
Galactosamine Trichloroacetimidates for the Formation of 1,2-*cis*-2-Amino Glycosides. Applications to the Synthesis of
Heparin Disaccharides, GPI Anchor Pseudodisaccharides, and α-GalNAc. J. Am. Chem. Soc..

[27] Cato D., Buskas T., Boons G. J. (2005). Highly Efficient Stereospecific Preparation
of Tn and TF Building Blocks Using Thioglycosyl Donors and the Ph_2_SO/Tf_2_O Promotor System. J. Carbohydr. Chem..

[28] Chen X.-T., Sames D., Danishefsky S. J. (1998). Exploration of Modalities in Building
α-*O*-Linked Systems through Glycal Assembly:
A Total Synthesis of the Mucin-Related F1a Antigen. J. Am. Chem. Soc..

[29] Yu F., McConnell M. S., Nguyen H. M. (2015). Scalable Synthesis of Fmoc-Protected
GalNAc-Threonine Amino Acid and T_N_ Antigen via Nickel Catalysis. Org. Lett..

[30] Xu Z., Deng Y., Zhang Z., Ma W., Li W., Wen L., Li T. (2021). Diversity-Oriented Chemoenzymatic Synthesis of Sulfated
and Nonsulfated Core 2 *O*-GalNAc Glycans. J. Org. Chem..

[31] Shou K., Zhang Y., Ji Y., Liu B., Zhou Q., Tan Q., Li F., Wang X., Lu G., Xiao G. (2024). Highly Stereoselective
a-Glycosylation with GalN_3_ Donors Enabled Collective Synthesis
of Mucin-Related Tumor Associated Carbohydrate Antigens. Chem. Sci..

[32] Kunz H., Birnbach S., Wernig P. (1990). Synthesis of Glycopeptides with the
Tn and T Antigen Structures, and Their Coupling to Bovine Serum Albumin. Carbohydr. Res..

[33] Nakahara Y., Iijima H., Sibayama S., Ogawa T. (1990). A Highly Stereoselective
Synthesis of Di- and Trimeric Sialosyltn Epitope: A Partial Structure
of Glycophorin A. Tetrahedron Lett..

[34] Elofsson M., A. Salvador L., Kihlberg J. (1997). Preparation of Tn and Sialyl Tn Building
Blocks Used in Fmoc Solid-Phase Synthesis of Glycopeptide Fragments
from HIV gp120. Tetrahedron.

[35] Kuduk S. D., Schwarz J. B., Chen X.-T., Glunz P. W., Sames D., Ragupathi G., Livingston P. O., Danishefsky S. J. (1998). Synthetic
and Immunological Studies on Clustered Modes of Mucin-Related Tn and
TF *O*-Linked Antigens: The Preparation of a Glycopeptide-Based
Vaccine for Clinical Trials against Prostate Cancer. J. Am. Chem. Soc..

[36] Winterfeld G. A., Ito Y., Ogawa T., Schmidt R. R. (1999). A Novel and Efficient Route towards
α-GalNAc-Ser and α-GalNAc-Thr Building Blocks for Glycopeptide
Synthesis. Eur. J. Org. Chem..

[37] Koeller K. M., Smith M. E. B., Wong C.-H. (2000). Chemoenzymatic
Synthesis of PSGL-1
Glycopeptides: Sulfation on Tyrosine Affects Glycosyltransferase-Catalyzed
Synthesis of the *O*-Glycan. Bioorg. Med. Chem..

[38] Kerns R. J., Zha C. X., Benakli K., Liang Y. Z. (2003). Extended
Applications
and Potential Limitations of Ring-Fused 2,3-Oxazolidinone Thioglycosides
in Glycoconjugate Synthesis. Tetrahedron Lett..

[39] Corzana F., Busto J. H., Jiménez-Osés G., García
de Luis M., Asensio J. L., Jiménez-Barbero J., Peregrina J. M., Avenoza A. (2007). Serine versus Threonine Glycosylation:
The Methyl Group Causes a Drastic Alteration on the Carbohydrate Orientation
and on the Surrounding Water Shell. J. Am. Chem.
Soc..

[40] Shaik A. A., Nishat S., Andreana P. R. (2015). Stereoselective
Synthesis of Natural
and Non-natural Thomsen-nouveau Antigens and Hydrazide Derivatives. Org. Lett..

[41] Medina S., Harper M. J., Balmond E. I., Miranda S., Crisenza G. E. M., Coe D. M., McGarrigle E. M., Galan M. C. (2016). Stereoselective
Glycosylation of 2-Nitrogalactals Catalyzed by a Bifunctional Organocatalyst. Org. Lett..

[42] Zhang M., Gan J., Peng P., Li T. (2025). Stereoselective
Synthesis of 1,2-*cis-O*-Linked Glycosyl Amino Acids
via Additive-Modulation
for Glycopeptide Synthesis. Chem.—Eur.
J..

[43] Li H., Zhang D., Li C., Yin L., Jiang Z., Luo Y., Xu H. (2024). Stereoselective Glycosylation
for 1,2-*cis*-Aminoglycoside Assembly by Cooperative
Atom Transfer Catalysis. J. Am. Chem. Soc..

[44] Lu D.-F., Zhu C.-L., Jia Z.-X., Xu H. (2014). Iron­(II)-Catalyzed
Intermolecular Amino-Oxygenation of Olefins through the N–O
Bond Cleavage of Functionalized Hydroxylamines. J. Am. Chem. Soc..

[45] Lu D.-F., Zhu C.-L., Sears J. D., Xu H. (2016). Iron­(II)-Catalyzed
Intermolecular Aminofluorination of Unfunctionalized Olefins Using
Fluoride Ion. J. Am. Chem. Soc..

[46] Radović A., Wolford N. J., Li H., Brennessel W. W., Xu H., Neidig M. L. (2023). Mechanistic Studies
of Iron-PyBOX-Catalyzed Olefin
Amino-Oxygenation with Functionalized Hydroxylamines. Organometallics.

[47] Chen A., Lili X., Zhenghong Z., Shiyin Z., Tianyi Y., Zhu F. (2021). Recent Advances in
Glycosylation Involving Novel Anomeric Radical
Precursors. J. Carbohydr. Chem..

[48] Chen A., Yang B., Zhou Z., Zhu F. (2022). Recent Advances in
Transition-metal-Catalyzed Glycosyl Cross-coupling Reactions. Chem. Catal.

