# Supplementary material for: Stereoselective Multigram-Scale Tn Antigen Synthesis via the Iron-Catalyzed Glycal 1,2-cis-Aminoglycosylation
Source: Org Lett. 2025 May 16;27(21):5515–20. doi: 10.1021/acs.orglett.5c01560 (PMC12124936; doi:10.1021/acs.orglett.5c01560)
Supplement: Supplementary file 1 [file ol5c01560_si_001.pdf]

Supporting Information

Stereoselective Multigram-Scale Tn Antigen Synthesis via the Iron-Catalyzed Glycal 1,2-*cis*-Aminoglycosylation

Le Yin,‡ Dakang Zhang,‡ Zixiang Jiang, and Hao Xu*

haohxu@brandeis.edu

Department of Chemistry, Brandeis University, 415 South Street, Waltham, MA 02453, USA

A. General Information

B. First-Generation Synthesis of Fully Protected Tn Antigens via the Iron-Catalyzed Glycal 1,2-*cis*-Aminoglycosylation

C. Second-Generation Synthesis of Fully Protected Tn Antigens with Challenging Galactosyl Donors via the Iron-Catalyzed Glycal 1,2-*cis*-Aminoglycosylation

D. Procedures for Post-glycosylation Transformations to Afford Tn Antigens and *O*-Galactosyl Amino Acids

E. References

F. NMR Spectra

A. General Information

General Procedures. All reactions were performed in oven-dried or flame-dried round-bottom flasks and vials. Stainless steel syringes and cannula were used to transfer air- and moisture-sensitive liquids. Flash chromatography was performed using silica gel 60 (230−400 mesh) from Sigma–Aldrich.

Materials. Commercial reagents were purchased from Sigma–Aldrich, TCI, Oakwood Chemicals, Combi-Blocks, Chem-Impex, Thermo Fischer Scientific and used as received. All solvents were used after being freshly distilled unless otherwise noted.

Instrumentation. Proton nuclear magnetic resonance (^{1}H NMR) spectra and carbon nuclear magnetic resonance (^{13}C NMR) spectra were recorded on Advance NEO 400 (400 MHz) and Varian 400-MR (400 MHz). Chemical shifts for protons are reported in parts per million downfield from tetramethylsilane and are referenced to the NMR solvent residual peak ($CHCl_3$ δ 7.26, CD_3OD δ 3.31, acetone-d_6 δ 2.05). Chemical shifts for carbons are reported in parts per million downfield from tetramethylsilane and are referenced to the carbon resonances of the NMR solvent ($CDCl_3$ δ 77.0, CD_3OD δ 49.0, acetone-d_6 δ 29.8). Data are represented as follows: chemical shift, multiplicity (br = broad, s = singlet, d = doublet, t = triplet, q = quartet, quint = quintet, m = multiplet), coupling constants in Hertz (Hz), and integration. The mass spectroscopic data were obtained at Brandeis Mass Spectrometry Facility using a Bruker timsTOF Pro instrument by electrospray ionization (ESI). Infrared (IR) spectra were obtained using a Nicolet IR200 spectrometer with a diamond ATR. Data are represented as follows: frequency of absorption (cm^{-1}) and absorption strength (s = strong, m = medium, w = weak). Optical rotations were measured on a Jasco P-2000 Polarimeter. The cuvette dimension is 10 cm and holds 1.5 mL.

Abbreviations Used: EtOAc–ethyl acetate, MeOH–methanol, Et_2O–diethyl ether, CH_2Cl_2–dichloromethane, MeCN–acetonitrile, DME–dimethoxyethane, THF–tetrahydrofuran, TBAF–tetra-*n*-butylammonium fluoride, AcOH–acetic acid, TFA–trifluoroacetic acid, DMAP–4-

dimethylaminopyridine, TMSOTf–trimethylsilyl trifluoromethanesulfonate, Ac_2O−acetic anhydride, FmocOSu–9-fluorenylmethyl *N*-succinimidyl carbonate.

B. First-Generation Synthesis of Fully Protected Tn Antigens via the Iron-Catalyzed Glycal 1,2-*cis*-Aminoglycosylation

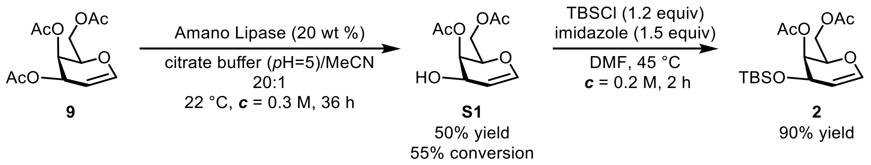

Figure S1. Synthesis of 4,6-di-*O*-Acetyl-3-*O*-TBS-D-Galactal via the Enzymatic Hydrolysis of tri-*O*-Acetyl-D-Galactal.

To a 500 mL round bottom flask were added *Amano Lipase from Pseudomonas fluorescens* (2.0 g, 20 wt %), 3,4,6-tri-*O*-acetyl-D-galactal (**9**)[1] (10.0 g, 36.73 mmol, 1.0 equiv), aqueous citric acid/trisodium citrate buffer (116.6 mL, *p*H = 5.0), and MeCN (5.8 mL). The mixture was stirred vigorously at room temperature for 17 h. EtOAc (150 mL) was added to dilute the reaction. The organic phase was separated from the aqueous phase and the aqueous phase was further extracted with EtOAc (100 mL × 4). The combined organic phase was washed with brine (20 mL) and dried over anhydrous Na_2SO_4. After concentration *in vacuo*, the residue was purified through column chromatography (hexanes/EtOAc: from 100:1 to 2:1) to afford the desired product **S1** (4.23 g, 50% yield) as colorless oil and the recovered starting material **9** (4.51 g, 45%).

The transformation of **S1** to **2** was carried out according to recent literature procedure reported by us.[2]

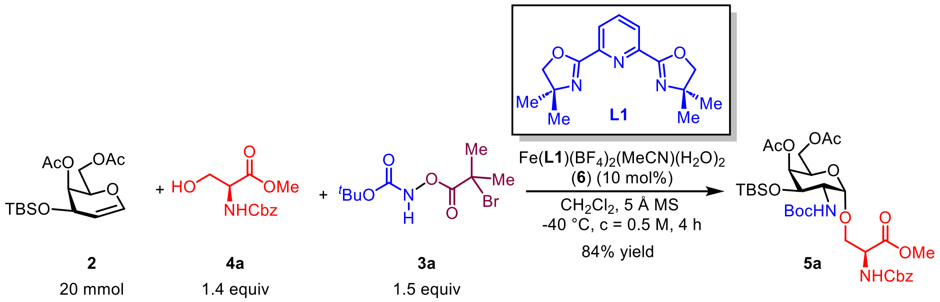

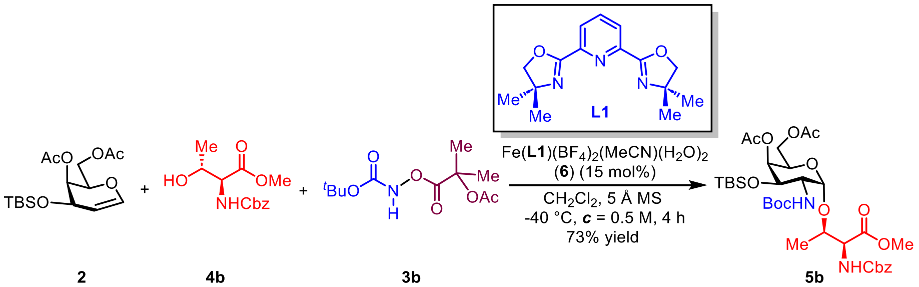

2 20 mmol | **4b** 1.4 equiv | **3b** 1.5 equiv | **5b**

These 1,2-*cis*-aminoglycosylation of 4,6-di-*O*-acetyl-3-*O*-TBS-D-galactal (**2**) with amino acid-derived glycosyl acceptors were carried out according to recent literature procedure reported by us.[2]

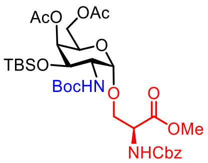

5a

***N*-Benzyloxycarbonyl-*O*-(4,6-di-*O*-aceyl-2-*tert*-butoxycarbonylamino-3-*O*-*tert*-butyldimethylsilyl-2-deoxy-α-D-galactopyranosyl)-L-serine methyl ester** (**5a**): $[\alpha]_D^{23}$ +78.5 (acetone, *c* = 1.0); IR ν_{max} (neat)/cm^{-1}: 2927 (w), 1747 (m), 1381 (m), 1137 (s), 1071 (m); ^{1}H NMR (400 MHz, $CDCl_3$) δ 7.40 – 7.28 (m, 5H), 5.76 (d, *J* = 8.2 Hz, 1H), 5.20 (d, *J* = 3.3 Hz, 1H), 5.18 – 5.06 (m, 2H), 4.77 (d, *J* = 3.7 Hz, 1H), 4.57 (dt, *J* = 7.9, 3.4 Hz, 1H), 4.36 (d, *J* = 10.2 Hz, 1H), 4.17 – 4.09 (m, 1H), 4.09 – 3.98 (m, 2H), 3.98 – 3.84 (m, 3H), 3.78 (s, 3H), 3.69 (dd, *J* = 10.4, 3.4 Hz, 1H), 2.08 (s, 3H), 2.01 (s, 3H), 1.40 (s, 9H), 0.81 (s, 9H),0.03 (s, 3H), 0.06 (s, 3H); ^{13}C NMR (100 MHz, $CDCl_3$) δ 170.5, 170.3, 170.2, 155.7, 154.9, 135.9, 128.5 (2C), 128.3, 128.2 (2C), 99.8, 79.6, 69.8, 69.5, 68.1, 67.8, 67.2, 62.7, 54.3, 52.6, 51.2, 28.4 (3C), 25.5 (3C), 20.7, 20.6, 17.6, -4.9, -5.1; HRMS: m/z (ESI) calcd for $C_{33}H_{53}N_2O_{13}Si^+$, $[M + H]^+$, 713.3311, found 713.3327. $^{1}J_{^{13}C1-H1}$ = 169.6 Hz.

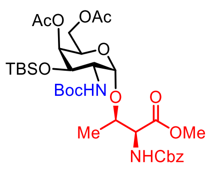

5b

***N*-Benzyloxycarbonyl-*O*-(4,6-di-*O*-aceyl-2-*tert*-butoxycarbonylamino-3-*O*-*tert*-butyldimethylsilyl-2-deoxy-α-D-galactopyranosyl)-L-threonine methyl ester (5b)**: $[\alpha]_D^{23}$ +69.6 (acetone, *c* = 1.0); IR ν_{max} (neat)/cm^{-1}: 2930 (w), 1718 (m), 1505 (m), 1366 (m), 1223 (m), 1167 (m), 947 (m), 697 (m); ^{1}H NMR (400 MHz, $CDCl_3$) δ 7.45 – 7.12 (m, 5H), 5.49 (d, *J* = 9.5 Hz, 1H), 5.17 (d, *J* = 3.4 Hz, 1H), 5.09 (s, 2H), 4.79 (d, *J* = 3.7 Hz, 1H), 4.48 – 4.38 (m, 2H), 4.18-4.17 (m, 1H), 4.07 – 3.87 (m, 4H), 3.75 (s, 3H), 3.65 (dd, *J* = 10.5, 3.4 Hz, 1H), 2.04 (s, 3H), 1.95 (s, 3H), 1.37 (s, 9H), 1.27 (d, *J* = 6.4 Hz, 3H), 0.77 (s, 9H), 0.03 (s, 3H), 0.01 (s, 3H); ^{13}C NMR (100 MHz, $CDCl_3$) δ 171.1, 170.4, 170.3, 156.4, 155.1, 135.9, 128.6 (2C), 128.4, 128.3 (2C), 100.8, 79.6, 77.5, 70.0, 68.1, 67.9, 67.5, 62.9, 58.4, 52.6, 51.5, 28.5 (3C), 25.6 (3C), 20.8, 20.6, 17.7, 17.6, -4.5, -5.0; HRMS: m/z (ESI) calcd for $C_{34}H_{55}N_2O_{13}Si^+$, $[M + H]^+$, 727.3468, found 727.3488. $^1J_{^{13}C1\text{–}H1}$ = 175.6 Hz.

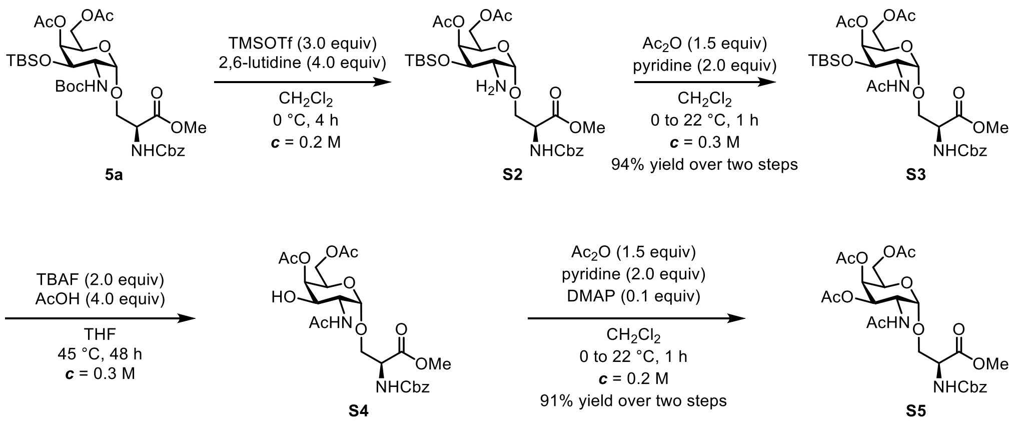

Figure S2. Post-glycosylation Transformations for Tn Antigen Synthesis.

The post-glycosylation deprotection procedure involved protecting group adjustment (*N*-Boc→*N*-Ac and OTBS→OAc). Both steps require lengthy reaction times and aqueous workups, which are not ideal.

C. Second-Generation Synthesis of Fully Protected Tn Antigens with Challenging Galactosyl Donors via the Iron-Catalyzed Glycal 1,2-*cis*-Aminoglycosylation

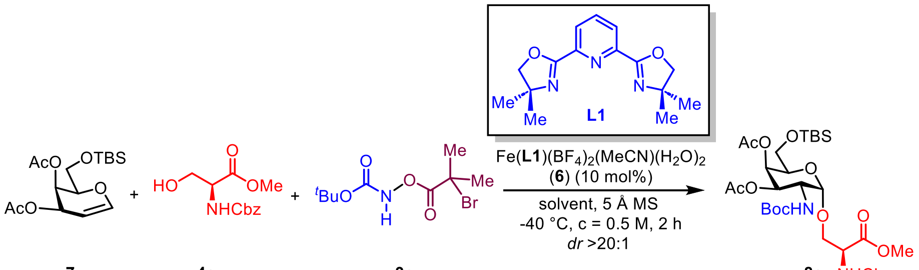

7 | 4a 1.4 equiv | 3a 1.5 equiv | 8a

entry	solvent	conversion (%)	yield of **8a** (%)
1	CH_2Cl_2	>95	68
2	CH_2Cl_2/Et_2O 9:1	<5	NA
3	CH_2Cl_2/THF 9:1	<5	NA
4	CH_2Cl_2/DME 9:1	<5	NA
5	CH_2Cl_2/1,4-dioxane 9:1	>95	86

Figure S3. Solvent Effect on the Iron-Catalyzed 1,2-*cis*-Aminoglycosylation with Challenging Galactosyl Donors.

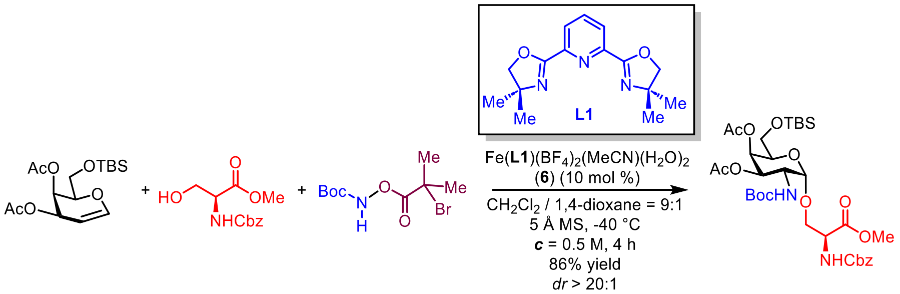

7 20 mmol | **4a** 1.4 equiv | **3a** 1.5 equiv | **8a**

3a was synthesized according to a literature procedure.[2] **4a** was synthesized according to a literature procedure.[3]

7 was synthesized according to the following procedure.

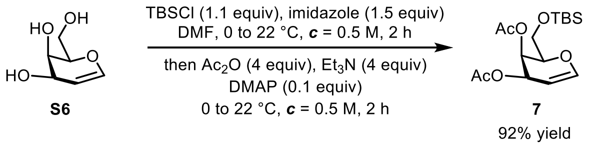

Figure S4. Synthesis of 3,4-di-*O*-Acetyl-6-*O*-TBS-D-Galactal (**7**) with a One-Pot Procedure.

To a flame-dried 250 mL round bottom flask were added D-galactal (**S6**) (5.85 g, 40 mmol, 1.0 equiv), imidazole (4.08 g, 60 mmol, 1.5 equiv), and anhydrous DMF (80 mL). The flask was cooled to 0 °C and TBSCl (6.63 g, 44 mmol, 1.1 equiv) was added in portions. The mixture was stirred at room temperature for 2 h with the progress monitored by TLC until completion. The flask was again cooled to 0 °C, and DMAP (489 mg, 4 mmol, 0.1 equiv), Et_3N (22.3 mL, 160 mmol, 4.0 equiv) were added. After stirring at 0 °C for 5 min, Ac_2O (15.1 mL, 160 mmol, 4.0 equiv) was added to the mixture. The reaction mixture was stirred for 2 h at 22 °C with the progress monitored by TLC until completion. The reaction was diluted with EtOAc (200 mL) and quenched with saturated aqueous NH_4Cl (240 mL). The aqueous phase was extracted with EtOAc (150 mL × 3). The combined organic phase was washed with brine (200 mL) and dried over anhydrous Na_2SO_4. After concentration *in vacuo*, the residue was purified through column chromatography (hexanes/EtOAc: from 100:1 to 8:1) to afford the desired product **7** (12.7 g, 92% yield) as colorless oil. The spectroscopic data of **7** match those reported in the literature.[4]

To a flame-dried 250 mL round bottom flask (flask **A**) equipped with a stir bar were added 3,4-di-*O*-acetyl-6-*O*-TBS-D-galactal (**7**) (6.89 g, 20 mmol, 1.0 equiv), *N*-Cbz serine methyl ester (**4a**) (7.09 g, 28 mmol, 1.4 equiv), iron catalyst Fe(**L1**)$(BF_4)_2$(MeCN)$(H_2O)_2$ (**6**) (1.16 g, 2 mmol, 10 mol %), and freshly activated 5 Å powdered molecular sieves (*ca.* 8 g). After the flask was evacuated and backfilled with N_2 twice, anhydrous CH_2Cl_2 (16 mL) and freshly distilled 1,4-dioxane (4 mL) were added and the flask was cooled to -78 °C. To a flame-dried 50 mL round bottom flask (flask **B**) was added acyloxyl carbamate **3a** (8.46 g, 30 mmol, 1.5 equiv). Flask **B** was evacuated and backfilled with N_2 twice and anhydrous CH_2Cl_2 (20 mL) was added. Then the solution in flask **B** was transferred to flask **A** via a syringe in 10 min. The reaction was kept

at -78 °C for an additional 3 min before switched to -40 °C. The reaction was kept at -40 °C for 4 h and quenched by precipitating the iron catalyst with Et_2O (80 mL) at the same temperature. The mixture was stirred for two minutes and subsequently warmed up to room temperature. The solution was then filtered through a short pad of Celite® and washed with saturated aq. $NaHCO_3$ solution (30 mL). The organic phase was separated from the aqueous one, which was further extracted with CH_2Cl_2 (30 mL × 3). The combined organic phase was dried over anhydrous Na_2SO_4 and concentrated *in vacuo*. The residue was purified through a silica gel flash column (hexanes/ethyl acetate: from 20:1 to 4:1) to afford the desired product **8a** as white foam (12.26 g, 86% yield).

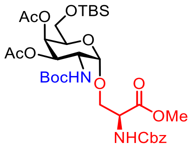

8a

***N*-Benzyloxycarbonyl-*O*-(3,4-di-*O*-acetyl-2-*tert*-butoxycarbonylamino-6-*O*-*tert*-butyldimethylsilyl-2-deoxy-α-D-galactopyranosyl)-L-serine methyl ester** (**8a**): $[\alpha]_D^{22}$ +52.1 (CH_2Cl_2, c = 1.0); IR ν_{max} (neat)/cm^{-1}: 3349 (w), 2954 (w), 2856 (w), 2362 (w), 1749 (s), 1720 (s), 1521 (m), 1456 (w), 1367 (m), 1242 (s), 1219 (s), 1171 (m), 1111 (m), 1067 (m), 1043(m), 839 (m), 778 (w); ^1H NMR (400 MHz, $CDCl_3$) δ 7.41 – 7.25 (m, 5H), 5.67 (d, J = 8.6 Hz, 1H), 5.39 (d, J = 3.7 Hz, 1H), 5.13 (ABq, $\Delta\nu_{AB}$ = 17.3 Hz, J_{AB} = 11.8 Hz, 2H), 4.98 (dd, J = 11.3, 3.2 Hz, 1H), 4.80 (d, J = 3.8 Hz, 1H), 4.56 (d, J = 9.6 Hz, 2H), 4.17 (td, J = 10.8, 3.7 Hz, 1H), 3.98 – 3.82 (m, 3H), 3.76 (s, 3H), 3.65 – 3.48 (m, 2H), 2.10 (s, 3H), 1.95 (s, 3H), 1.39 (s, 9H), 0.83 (s, 9H), −0.01 (s, 3H), −0.02 (s, 3H); ^{13}C NMR (100 MHz, $CDCl_3$) δ 170.5, 170.4, 170.0, 155.8, 155.2, 136.0, 128.5 (2C), 128.2, 128.1 (2C), 99.4, 79.8, 69.9, 69.6, 69.2, 67.2 (two peaks overlapped, 2C), 60.9, 54.2, 52.7, 48.9, 28.2 (3C), 24.9 (3C), 20.72, 20.66, 18.1, -5.6, -5.7; HRMS: m/z (ESI) calcd for $C_{33}H_{53}N_2O_{13}Si^+$, $[M + H]^+$, 713.3311, found 713.3329. $^1J_{^{13}C1-H1}$ =175.4 Hz.

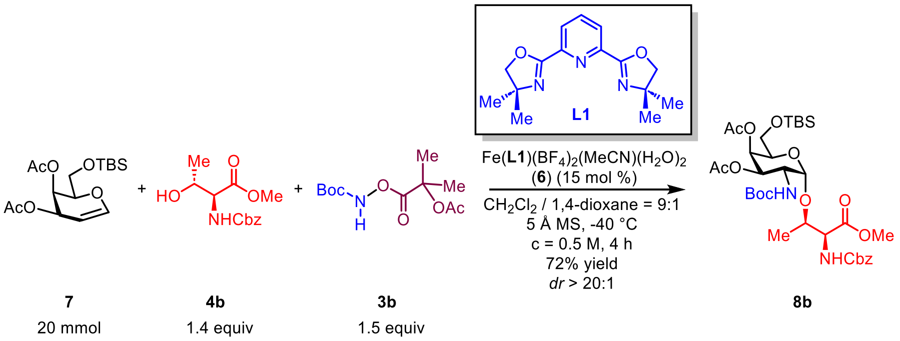

3b was synthesized according to a literature procedure.[2] **4b** was synthesized according to a literature procedure.[3]

To a flame-dried 250 mL round bottom flask (flask **A**) equipped with a stir bar were added 3,4-di-*O*-acetyl-6-*O*-TBS-D-galactal (**7**) (6.89 g, 20 mmol, 1.0 equiv), *N*-Cbz threonine methyl ester (**4b**) (7.48 g, 28 mmol, 1.4 equiv), iron catalyst Fe(**L1**)$(BF_4)_2$(MeCN)$(H_2O)_2$ (**6**) (1.74 g, 3 mmol, 15 mol %), and freshly activated 5 Å powdered molecular sieves (*ca.* 8 g). After the flask was evacuated and backfilled with N_2 twice, anhydrous CH_2Cl_2 (16 mL) and freshly distilled 1,4-dioxane (4 mL) were added and the flask was cooled to -78 °C. To a flame-dried 50 mL round bottom flask (flask **B**) was added acyloxyl carbamate **3b** (7.84 g, 30 mmol, 1.5 equiv). Flask **B** was evacuated and backfilled with N_2 twice and anhydrous CH_2Cl_2 (20 mL) was added. Then the solution in flask **B** was transferred to flask **A** via a syringe in 10 min. The reaction was kept at -78 °C for an additional 3 min before switched to -40 °C. The reaction was kept at -40 °C for 4 h and quenched by precipitating the iron catalyst with Et_2O (80 mL) at the same temperature. The mixture was stirred for two minutes and subsequently warmed up to room temperature. The solution was then filtered through a short pad of Celite® and washed with saturated aq. $NaHCO_3$ solution (30 mL). The organic phase was separated from the aqueous one, which was further extracted with CH_2Cl_2 (30 mL × 3). The combined organic phase was dried over anhydrous Na_2SO_4 and concentrated *in vacuo*. The residue was purified through a silica gel flash column (hexanes/ethyl acetate: from 20:1 to 4:1) to afford the desired product **8b** as white foam (10.47 g, 72% yield).

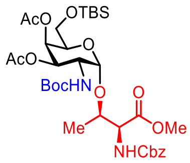

8b

***N*-Benzyloxycarbonyl-*O*-(3,4-di-*O*-acetyl-2-*tert*-butoxycarbonylamino-6-*O*-*tert*-butyldimethylsilyl-2-deoxy-α-D-galactopyranosyl)-L-threonine methyl ester (8b)**: $[\alpha]_D^{22}$ +57.4 (CH_2Cl_2, c = 1.0); IR ν_{max} (neat)/cm^{-1}: 3339 (w), 2930 (w), 2857 (w), 1749 (s), 1716 (s), 1511 (m), 1456 (w), 1367 (m), 1310 (m), 1242 (s), 1220 (s), 1171 (m), 1137 (m), 1098 (s), 1041 (s), 1006 (s), 947 (w), 921 (w), 838 (s), 777 (m); ^{1}H NMR (400 MHz, $CDCl_3$) δ 7.43 – 7.29 (m, 5H), 5.47 (d, J = 9.5 Hz, 1H), 5.43 – 5.37 (m, 1H), 5.14 (s, 2H), 4.97 (dd, J = 11.3, 3.2 Hz, 1H), 4.89 (d, J = 3.8 Hz, 1H), 4.62 (d, J = 10.3 Hz, 1H), 4.45 (dd, J = 9.5, 2.5 Hz, 1H), 4.30 – 4.21 (m, 1H), 4.17 (td, J = 10.7, 3.7 Hz, 1H), 4.01 (t, J = 6.6 Hz, 1H), 3.78 (s, 3H), 3.63 – 3.50 (m, 2H), 2.12 (s, 3H), 1.97 (s, 3H), 1.42 (s, 9H), 1.33 (d, J = 6.4 Hz, 3H), 0.85 (s, 9H), 0.01 (s, 3H), 0.00 (s, 3H); ^{13}C NMR (100 MHz, $CDCl_3$) δ 171.0, 170.6, 170.1, 156.4, 155.2, 135.9, 128.5 (2C), 128.2, 128.1 (2C), 100.2, 79.6, 77.2, 70.0, 69.4, 67.5, 67.3, 61.3, 58.3, 52.6, 49.0, 28.2 (3C), 25.7 (3C), 20.8, 20.7, 18.1, 18.0, -5.6, -5.7; HRMS: m/z (ESI) calcd for $C_{34}H_{55}N_2O_{13}Si^+$, $[M + H]^+$, 727.3468, found 727.3457. $^1J_{^{13}C1\text{–}H1}$ =171.7 Hz.

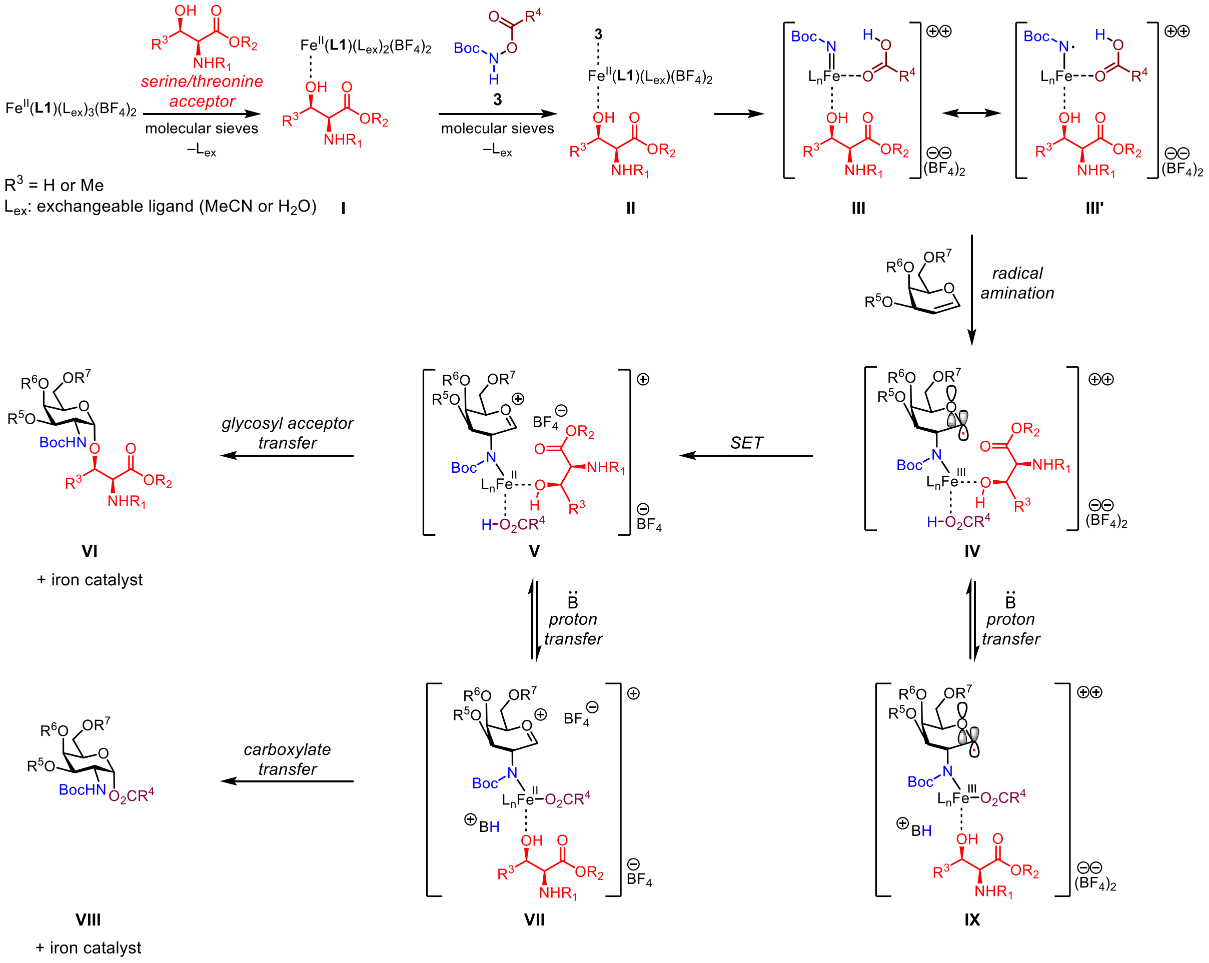

Figure S5. Mechanistic Working Hypothesis for the Iron-Catalyzed *cis*-Aminoglycosylation of Galactal Donors with Serine/Threonine Acceptors.

Our mechanistic studies suggested that the iron catalyst activates a serine or threonine glycosyl acceptor and amination reagent **3** when it facilitates the cooperative atom transfer of both moieties to a glycosyl donor in an exclusively *cis*-selective manner. Notably, both a 2-amidoglycosyl radical and a 2-amidoglycosyl oxocarbenium ion are plausible intermediates.

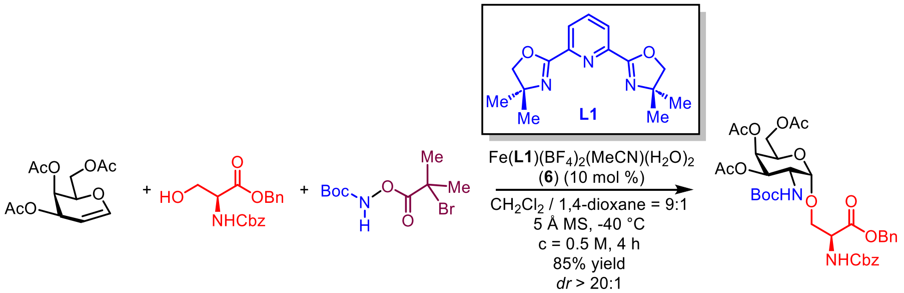

10a was synthesized according to a literature procedure.[5]

To a flame-dried 100 mL round bottom flask (flask **A**) equipped with a stir bar were added tri-*O*-acetyl-D-galactal (**9**) (2.72 g, 10 mmol, 1.0 equiv), *N*-Cbz serine benzyl ester (**10a**) (4.61 g, 14 mmol, 1.4 equiv), iron catalyst Fe(**L1**)$(BF_4)_2$(MeCN)$(H_2O)_2$ (**6**) (580 mg, 1 mmol, 10 mol %), and freshly activated 5 Å powdered molecular sieves (*ca.* 4 g). After the flask was evacuated and backfilled with N_2 twice, anhydrous CH_2Cl_2 (8 mL) and freshly distilled 1,4-dioxane (2 mL) were added and the flask was cooled to -78 °C. To a flame-dried 25 mL round bottom flask (flask **B**) was added acyloxyl carbamate **3a** (4.23 g, 15 mmol, 1.5 equiv). Flask **B** was evacuated and backfilled with N_2 twice and anhydrous CH_2Cl_2 (10 mL) was added. Then the solution in flask **B** was transferred to flask **A** via a syringe in 10 min. The reaction was kept at -78 °C for an additional 3 min before switched to -40 °C. The reaction was kept at -40 °C for 4 h and quenched by precipitating the iron catalyst with Et_2O (40 mL) at the same temperature. The mixture was stirred for two minutes and subsequently warmed up to room temperature. The solution was then filtered through a short pad of Celite® and washed with saturated aq. $NaHCO_3$ solution (15 mL). The organic phase was separated from the aqueous one, which was further extracted with CH_2Cl_2 (15 mL × 3). The combined organic phase was dried over anhydrous Na_2SO_4 and concentrated *in vacuo*. The residue was purified through a silica gel flash column (hexanes/acetone: from 20:1 to 5:1) to afford the desired product **11a** as white foam (6.09 g, 85% yield).

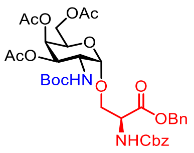

11a

***N*-Benzyloxycarbonyl-*O*-(3,4,6-tri-*O*-acetyl-2-*tert*-butoxycarbonylamino-2-deoxy-α-D-galactopyranosyl)-L-serine benzyl ester** (**11a**): $[\alpha]_D^{22}$ +61.2 (acetone, *c* = 1.0); IR ν_{max} (neat)/cm^{-1}: 3357 (w), 2972 (w), 2360 (w), 1747 (s), 1716 (s), 1521 (m), 1456 (w), 1368 (m), 1226 (s), 1168 (m), 1133 (m), 1044 (m), 951 (w), 753 (w); ^{1}H NMR (400 MHz, $CDCl_3$) δ 7.42 – 7.30 (m, 10H), 5.80 (d, *J* = 8.3 Hz, 1H), 5.29 (d, *J* = 3.2 Hz, 1H), 5.25 – 5.19 (m, 2H), 5.12 (ABq, $\Delta\nu_{AB}$ = 13.5 Hz, J_{AB} = 12.2 Hz, 2H), 4.93 (dd, *J* = 11.3, 3.2 Hz, 1H), 4.79 (d, *J* = 3.8 Hz, 1H), 4.61 (d, *J* = 7.6 Hz, 1H), 4.54 (d, *J* = 10.2 Hz, 1H), 4.21 (td, *J* = 10.7, 3.7 Hz, 1H), 4.06 (d, *J* = 5.2 Hz, 1H), 4.03 – 3.92 (m, 4H), 2.14 (s, 3H), 2.00 (s, 3H), 1.99 (s, 3H), 1.41 (s, 9H); ^{13}C NMR (100 MHz, $CDCl_3$) δ 170.4 (two peaks overlapped, 2C), 170.2, 169.7, 155.8, 155.2, 136.0, 134.8, 128.8 (two peaks overlapped, 4C), 128.6 (2C), 128.5 (2C), 128.3, 128.1, 99.7, 80.0, 70.3, 68.8, 67.8, 67.3 (two peaks overlapped, 2C), 67.2, 61.9, 54.5, 48.8, 28.2 (3C), 20.7, 20.64, 20.60; HRMS: m/z (ESI) calcd for $C_{35}H_{45}N_2O_{14}^+$, $[M + H]^+$, 717.2865, found 717.2872. $^1J_{^{13}C1-H1}$ = 170.8 Hz.

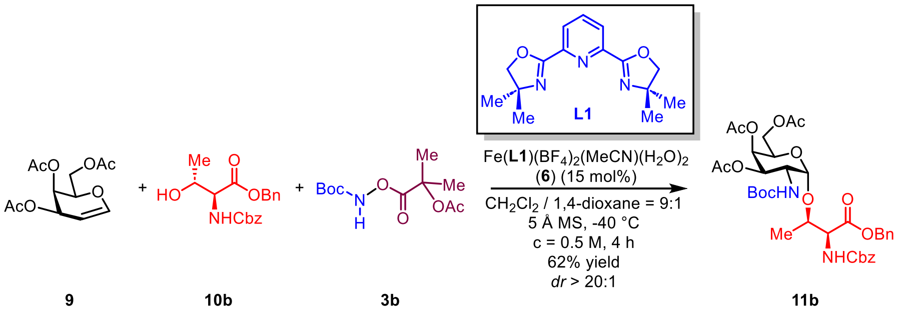

9 10 mmol | **10b** 1.4 equiv | **3b** 1.5 equiv | **11b**

10b was synthesized according to a literature procedure.[5]

To a flame-dried 100 mL round bottom flask (flask **A**) equipped with a stir bar were added tri-*O*-D-acetyl galactal (**9**) (2.72 g, 10 mmol, 1.0 equiv), *N*-Cbz threonine benzyl ester (**10b**) (4.81 g,

14 mmol, 1.4 equiv), iron catalyst Fe(**L1**)$(BF_4)_2$(MeCN)$(H_2O)_2$ (**6**) (870 mg, 1.5 mmol, 15 mol %), and freshly activated 5 Å powdered molecular sieves (*ca.* 4 g). After the flask was evacuated and backfilled with N_2 twice, anhydrous CH_2Cl_2 (8 mL) and freshly distilled 1,4-dioxane (2 mL) were added and the flask was cooled to -78 °C. To a flame-dried 25 mL round bottom flask (flask **B**) was added acyloxyl carbamate **3b** (3.92 g, 15 mmol, 1.5 equiv). Flask **B** was evacuated and backfilled with N_2 twice and anhydrous CH_2Cl_2 (10 mL) was added. Then the solution in flask **B** was transferred to flask **A** via a syringe in 10 min. The reaction was kept at -78 °C for an additional 3 min before switched to -40 °C. The reaction was kept at -40 °C for 4 h and quenched by precipitating the iron catalyst with Et_2O (40 mL) at the same temperature. The mixture was stirred for two minutes and subsequently warmed up to room temperature. The solution was then filtered through a short pad of Celite® and washed with saturated aq. $NaHCO_3$ solution (15 mL). The organic phase was separated from the aqueous one, which was further extracted with CH_2Cl_2 (15 mL × 3). The combined organic phase was dried over anhydrous Na_2SO_4 and concentrated *in vacuo*. The residue was purified through a silica gel flash column (hexanes/acetone: from 20:1 to 5:1) to afford the desired product **11b** as white foam (4.53 g, 62% yield).

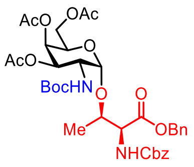

11b

***N*-Benzyloxycarbonyl-*O*-(3,4,6-tri-*O*-acetyl-2-*tert*-butoxycarbonylamino-2-deoxy-α-D-galactopyranosyl)-L-threonine benzyl ester** (**11b**): $[\alpha]_D^{22}$ +52.8 (acetone, *c* = 1.0); IR ν_{max} (neat)/cm^{-1}: 3357 (w), 2977 (w), 2360 (w), 1748 (s), 1717 (s), 1508 (m), 1456 (w), 1368 (m), 1308 (m), 1222 (s), 1168 (m), 1134 (m), 1055 (m), 1010 (m), 923 (w), 754 (w); ^{1}H NMR (400 MHz, $CDCl_3$) δ 7.43 – 7.31 (m, 10H), 5.49 (d, *J* = 9.5 Hz, 1H), 5.36 (d, *J* = 2.3 Hz, 1H), 5.20 (s, 2H), 5.14 (s, 2H), 4.95 (dd, *J* = 11.3, 3.2 Hz, 1H), 4.83 (d, *J* = 3.8 Hz, 1H), 4.69 (d, *J* = 10.4 Hz, 1H), 4.47 (dd, *J* = 9.5, 2.4 Hz, 1H), 4.28 – 4.19 (m, 2H), 4.16 (t, *J* = 6.4 Hz, 1H), 4.10 – 4.00 (m, 2H), 2.15 (s, 3H), 2.01 (s, 3H), 1.98 (s, 3H), 1.41 (s, 9H), 1.31 (d, *J* = 6.4 Hz, 3H); ^{13}C NMR (100 MHz, $CDCl_3$) δ 170.39, 170.38, 170.2, 170.1, 156.4, 155.1, 135.9, 134.5, 128.7 (2C), 128.6

(two peaks overlapped, 4C), 128.4 (2C), 128.1, 128.0, 100.4, 79.7, 77.7, 69.0, 67.6, 67.4, 67.21, 67.17, 62.0, 58.4, 48.8, 28.1 (3C), 20.6, 20.50, 20.46, 17.7; HRMS: m/z (ESI) calcd for $C_{36}H_{47}N_2O_{14}^+$, $[M + H]^+$, 731.3022, found 731.2998. $^1J_{^{13}C1-H1}$ = 170.4 Hz.

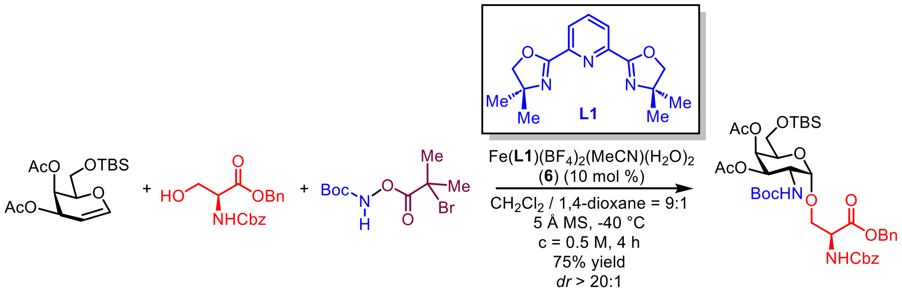

To a flame-dried 100 mL round bottom flask (flask **A**) equipped with a stir bar were added 3,4-di-*O*-acetyl-6-*O*-TBS-D-galactal (**7**) (3.44 g, 10 mmol, 1.0 equiv), *N*-Cbz serine benzyl ester (**10a**) (4.61 g, 14 mmol, 1.4 equiv), iron catalyst Fe(**L1**)$(BF_4)_2$(MeCN)$(H_2O)_2$ (**6**) (580 mg, 1 mmol, 10 mol %), and freshly activated 5 Å powdered molecular sieves (*ca.* 4 g). After the flask was evacuated and backfilled with N_2 twice, anhydrous CH_2Cl_2 (8 mL) and freshly distilled 1,4-dioxane (2 mL) were added and the flask was cooled to -78 °C. To a flame-dried 25 mL round bottom flask (flask **B**) was added acyloxyl carbamate **3a** (4.23 g, 15 mmol, 1.5 equiv). Flask **B** was evacuated and backfilled with N_2 twice and anhydrous CH_2Cl_2 (10 mL) was added. Then the solution in flask **B** was transferred to flask **A** via a syringe in 10 min. The reaction was kept at -78 °C for an additional 3 min before switched to -40 °C. The reaction was kept at -40 °C for 4 h and quenched by precipitating the iron catalyst with Et_2O (40 mL) at the same temperature. The mixture was stirred for two minutes and subsequently warmed up to room temperature. The solution was then filtered through a short pad of Celite® and washed with saturated aq. $NaHCO_3$ solution (15 mL). The organic phase was separated from the aqueous one, which was further extracted with CH_2Cl_2 (15 mL × 3). The combined organic phase was dried over anhydrous Na_2SO_4 and concentrated *in vacuo*. The residue was purified through a silica gel flash column (hexanes/ethyl acetate: from 20:1 to 5:1) to afford the desired product **12a** as white foam (5.92 g, 75% yield).

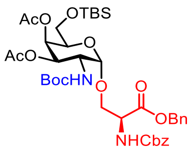

12a

***N*-Benzyloxycarbonyl-*O*-(3,4-di-*O*-acetyl-2-*tert*-butoxycarbonylamino-6-*O*-*tert*-butyldimethylsilyl-2-deoxy-α-D-galactopyranosyl)-L-serine benzyl ester** (**12a**): $[\alpha]_D^{22}$ +38.3 (acetone, *c* = 1.0); IR ν_{max} (neat)/cm^{-1}: 3350 (w), 2930 (w), 2857 (w), 1749 (s), 1719 (s), 1508 (m), 1456 (w), 1367 (m), 1242 (s), 1220 (s), 1170 (m), 1143 (m), 949 (w), 838 (m), 778 (m), 753 (w); ^{1}H NMR (400 MHz, $CDCl_3$) δ 7.41 – 7.28 (m, 10H), 5.74 (d, *J* = 8.5 Hz, 1H), 5.39 (d, *J* = 3.2 Hz, 1H), 5.20 (s, 2H), 5.12 (ABq, $\Delta\nu_{AB}$ = 17.0 Hz, J_{AB} = 12.0 Hz, 2H), 4.97 (dd, *J* = 11.2, 3.2 Hz, 1H), 4.76 (d, *J* = 3.7 Hz, 1H), 4.59 (m, 2H), 4.19 (td, *J* = 10.7, 3.7 Hz, 1H), 4.01 – 3.84 (m, 3H), 3.61 (dd, *J* = 9.8, 6.3 Hz, 1H), 3.53 (dd, *J* = 9.9, 7.2 Hz, 1H), 2.12 (s, 3H), 1.98 (s, 3H), 1.40 (s, 9H), 0.84 (s, 9H), 0.00 (s, 3H), -0.01 (s, 3H); ^{13}C NMR (100 MHz, $CDCl_3$) δ 170.5, 170.0, 169.8, 155.9, 155.2, 136.0, 134.8, 128.73 (2C), 128.70 (2C), 128.52 (2C), 128.49 (2C), 128.2, 128.1, 99.7, 79.8, 69.9, 69.8, 69.3, 67.7, 67.23, 67.21, 60.9, 54.4, 49.0, 28.2 (3C), 25.7 (3C), 20.8, 20.7, 18.1, -5.6, -5.7; HRMS: m/z (ESI) calcd for $C_{39}H_{57}N_2O_{13}Si^+$, $[M + H]^+$, 789.3624, found 789.3609. $^1J_{^{13}C1\text{–}H1}$ = 172.0 Hz.

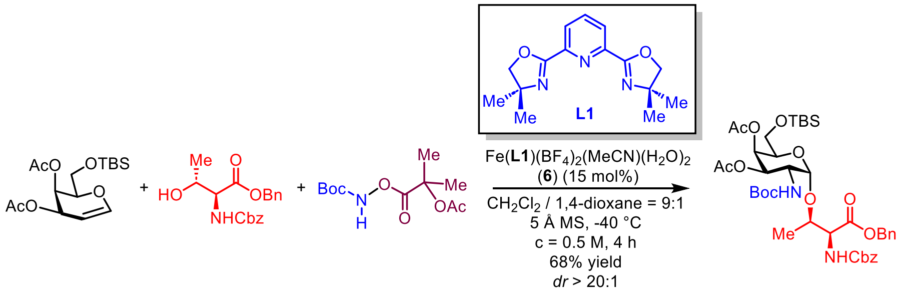

7 10 mmol; **10b** 1.4 equiv; **3b** 1.5 equiv; **12b**

To a flame-dried 100 mL round bottom flask (flask **A**) equipped with a stir bar were added 3,4-di-*O*-acetyl-6-*O*-TBS-D-galactal (**7**) (3.44 g, 10 mmol, 1.0 equiv), *N*-Cbz threonine benzyl ester (**10b**) (4.81 g, 14 mmol, 1.4 equiv), iron catalyst Fe(**L1**)$(BF_4)_2(MeCN)(H_2O)_2$ (**6**) (870 mg, 1.5 mmol, 15 mol %), and freshly activated 5 Å powdered molecular sieves (*ca.* 4 g). After the flask

was evacuated and backfilled with N_2 twice, anhydrous CH_2Cl_2 (8 mL) and freshly distilled 1,4-dioxane (2 mL) were added and the flask was cooled to -78 °C. To a flame-dried 25 mL round bottom flask (flask **B**) was added acyloxyl carbamate **3b** (3.92 g, 15 mmol, 1.5 equiv). Flask **B** was evacuated and backfilled with N_2 twice and anhydrous CH_2Cl_2 (10 mL) was added. Then the solution in flask **B** was transferred to flask **A** via a syringe in 10 min. The reaction was kept at -78 °C for an additional 3 min before switched to -40 °C. The reaction was kept at -40 °C for 4 h and quenched by precipitating the iron catalyst with Et_2O (40 mL) at the same temperature. The mixture was stirred for two minutes and subsequently warmed up to room temperature. The solution was then filtered through a short pad of Celite® and washed with saturated aq. $NaHCO_3$ solution (15 mL). The organic phase was separated from the aqueous one, which was further extracted with CH_2Cl_2 (15 mL × 3). The combined organic phase was dried over anhydrous Na_2SO_4 and concentrated *in vacuo*. The residue was purified through a silica gel flash column (hexanes/acetone: from 20:1 to 5:1) to afford the desired product **12b** as white foam (5.46 g, 68% yield).

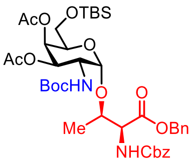

12b

***N*-Benzyloxycarbonyl-*O*-(3,4-di-*O*-acetyl-2-*tert*-butoxycarbonylamino-6-*O*-*tert*-butyldimethylsilyl-2-deoxy-α-D-galactopyranosyl)-L-threonine benzyl ester (12b)**: $[\alpha]_D^{22}$ +35.9 (acetone, *c* = 1.0); IR ν_{max} (neat)/cm^{-1}: 3349 (w), 2930 (w), 2857 (w), 2360 (w), 1967 (w), 1750 (s), 1719 (s), 1507 (m), 1456 (w), 1367 (m), 1308 (m), 1245 (s), 1221 (s), 1169 (m), 1140 (m), 1108 (m), 1042 (m), 1006 (m), 948 (w), 923 (w), 838 (m), 777 (m); ^{1}H NMR (400 MHz, $CDCl_3$) δ 7.42 – 7.29 (m, 10H), 5.50 (d, *J* = 9.5 Hz, 1H), 5.40 (d, *J* = 3.2 Hz, 1H), 5.19 (s, 2H), 5.13 (s, 2H), 4.97 (dd, *J* = 11.3, 3.2 Hz, 1H), 4.80 (d, *J* = 3.7 Hz, 1H), 4.70 (d, *J* = 10.4 Hz, 1H), 4.46 (dd, *J* = 9.5, 2.4 Hz, 1H), 4.26 – 4.13 (m, 2H), 4.00 (t, *J* = 6.6 Hz, 1H), 3.62 – 3.49 (m, 2H), 2.13 (s, 3H), 1.97 (s, 3H), 1.41 (s, 9H), 1.31 (d, J = 6.4 Hz, 3H), 0.84 (s, 9H), 0.00 (s, 3H), -0.01 (s, 3H); ^{13}C NMR (100 MHz, $CDCl_3$) 170.54, 170.51, 170.1, 156.4, 154.8, 135.9, 134.7, 128.8 (two peaks overlapped, 4C), 128.7 (2C), 128.5 (2C), 128.2, 128.1, 100.5, 79.4, 77.6, 70.0, 69.5,

67.7, 67.5, 67.3, 61.3, 58.5, 49.1, 28.2 (3C), 25.7 (3C), 20.8, 20.7, 18.1, 18.0, -5.6, -5.7; HRMS: m/z (ESI) calcd for $C_{40}H_{59}N_2O_{13}Si^+$, $[M + H]^+$, 803.3781, found 803.3776. $^1J_{^{13}C1\text{–}H1}$ = 174.0 Hz.

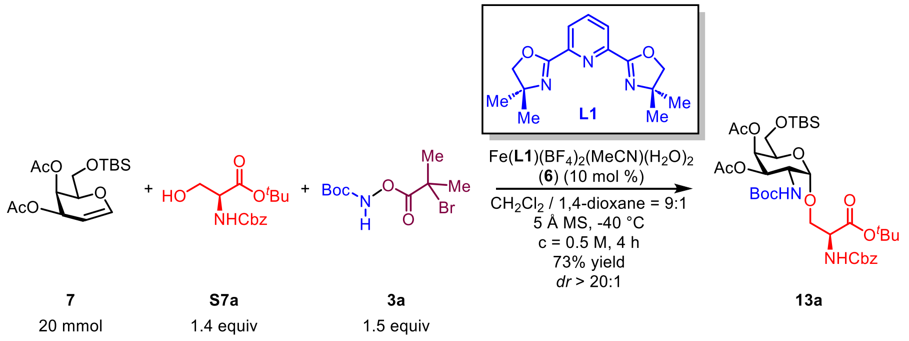

S7a was synthesized according to a literature procedure.[6]

To a flame-dried 250 mL round bottom flask (flask **A**) equipped with a stir bar were added 3,4-di-*O*-acetyl-6-*O*-TBS-D-galactal (**7**) (6.89 g, 20 mmol, 1.0 equiv), *N*-Cbz serine *tert*-butyl ester (**S7a**) (8.27 g, 28 mmol, 1.4 equiv), iron catalyst Fe(**L1**)$(BF_4)_2$(MeCN)$(H_2O)_2$ (**6**) (1.16 g, 2 mmol, 10 mol %), and freshly activated 5 Å powdered molecular sieves (*ca*. 8 g). After the flask was evacuated and backfilled with N_2 twice, anhydrous CH_2Cl_2 (16 mL) and freshly distilled 1,4-dioxane (4 mL) were added and the flask was cooled to -78 °C. To a flame-dried 50 mL round bottom flask (flask **B**) was added acyloxyl carbamate **3a** (8.46 g, 30 mmol, 1.5 equiv). Flask **B** was evacuated and backfilled with N_2 twice and anhydrous CH_2Cl_2 (20 mL) was added. Then the solution in flask **B** was transferred to flask **A** via a syringe in 10 min. The reaction was kept at -78 °C for an additional 3 min before switched to -40 °C. The reaction was kept at -40 °C for 4 h and quenched by precipitating the iron catalyst with Et_2O (80 mL) at the same temperature. The mixture was stirred for two minutes and subsequently warmed up to room temperature. The solution was then filtered through a short pad of Celite® and washed with saturated aq. $NaHCO_3$ solution (30 mL). The organic phase was separated from the aqueous one, which was further extracted with CH_2Cl_2 (30 mL × 3). The combined organic phase was dried over anhydrous Na_2SO_4 and concentrated *in vacuo*. The residue was purified through a silica gel flash column (hexanes/ethyl acetate: from 20:1 to 6:1) to afford the desired product **13a** as white foam (11.02 g, 73% yield).

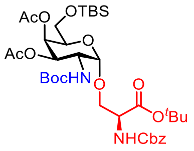

13a

***N*-Benzyloxycarbonyl-*O*-(3,4-di-*O*-acetyl-2-*tert*-butoxycarbonylamino-6-*O*-*tert*-butyldimethylsilyl-2-deoxy-α-D-galactopyranosyl)-L-serine *tert*-butyl ester (13a)**: $[\alpha]_D^{22}$ +56.8 (CH_2Cl_2, c = 1.0); IR ν_{max} (neat)/cm^{-1}: 3352 (w), 2970 (w), 2360 (w), 1739 (s), 1726 (s), 1506 (w), 1455 (w), 1366 (s), 1227 (s), 1217 (s), 1156 (m), 1110 (m), 1042 (m), 1005 (m), 928 (w), 837 (m), 776 (m); ^{1}H NMR (400 MHz, $CDCl_3$) δ 7.43 – 7.27 (m, 5H), 5.61 (d, J = 8.6 Hz, 1H), 5.42 (s, 1H), 5.13 (ABq, $\Delta\nu_{AB}$ = 26 Hz, J_{AB} = 12 Hz, 2H), 5.02 (d, J = 11.2 Hz, 1H), 4.83 (s, 1H), 4.60 (d, J = 10.3 Hz, 1H), 4.43 (d, J = 9.3 Hz, 1H), 4.21 (t, J = 10.9 Hz, 1H), 3.99-3.88 (m, 2H), 3.83 (d, J = 10.3 Hz, 1H), 3.66 – 3.51 (m, 2H), 2.12 (s, 3H), 1.97 (s, 3H), 1.47 (s, 9H), 1.40 (s, 9H), 0.84 (s, 9H), 0.01 (s, 3H), 0.00 (s, 3H); ^{13}C NMR (100 MHz, $CDCl_3$) δ 170.6, 170.2, 169.0, 156.0, 155.3, 136.3, 128.6 (2C), 128.3(two peaks overlapped, 3C), 99.3, 83.0, 79.8, 69.9, 69.7, 69.4, 67.4, 67.3, 61.1, 54.7, 49.1, 28.3 (3C), 28.2 (3C), 25.9 (3C), 20.9, 20.8, 18.3, -5.5, -5.6; HRMS: m/z (ESI) calcd for $C_{36}H_{59}N_2O_{13}Si^+$, $[M + H]^+$, 755.3781, found 755.3796. $^1J^{13}{}_{C1-H1}$ = 171.9Hz.

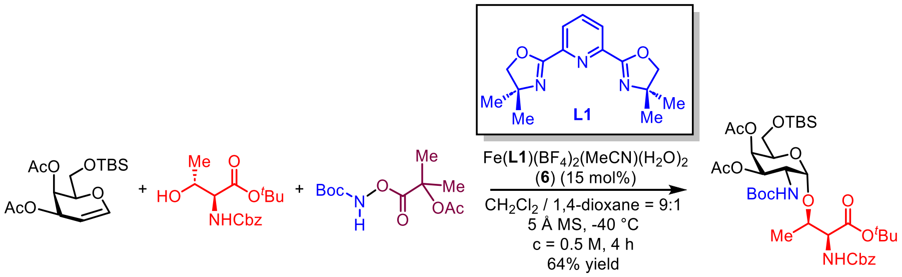

7 20 mmol; **S7b** 1.4 equiv; **3b** 1.5 equiv; **13b**

S7b was synthesized according to a literature procedure.[6]

To a flame-dried 250 mL round bottom flask (flask **A**) equipped with a stir bar were added 3,4-di-*O*-acetyl-6-*O*-TBS-D-galactal (**7**) (6.89 g, 20 mmol, 1.0 equiv), *N*-Cbz threonine *tert*-butyl

ester (**S7b**) (8.66 g, 28 mmol, 1.4 equiv), iron catalyst Fe(**L1**)$(BF_4)_2$(MeCN)$(H_2O)_2$ (**6**) (1.74 g, 3 mmol, 15 mol %), and freshly activated 5 Å powdered molecular sieves (*ca*. 8 g). After the flask was evacuated and backfilled with N_2 twice, anhydrous CH_2Cl_2 (16 mL) and freshly distilled 1,4-dioxane (4 mL) were added and the flask was cooled to -78 °C. To a flame-dried 50 mL round bottom flask (flask **B**) was added acyloxyl carbamate **3b** (7.84 g, 30 mmol, 1.5 equiv). Flask **B** was evacuated and backfilled with N_2 twice and anhydrous CH_2Cl_2 (20 mL) was added. Then the solution in flask **B** was transferred to flask **A** via a syringe in 10 min. The reaction was kept at -78 °C for an additional 3 min before switched to -40 °C. The reaction was kept at -40 °C for 4 h and quenched by precipitating the iron catalyst with Et_2O (80 mL) at the same temperature. The mixture was stirred for two minutes and subsequently warmed up to room temperature. The solution was then filtered through a short pad of Celite® and washed with saturated aq. $NaHCO_3$ solution (30 mL). The organic phase was separated from the aqueous one, which was further extracted with CH_2Cl_2 (30 mL × 3). The combined organic phase was dried over anhydrous Na_2SO_4 and concentrated *in vacuo*. The residue was purified through a silica gel flash column (hexanes/ethyl acetate: from 20:1 to 6:1) to afford the desired product **13b** as white foam (9.84 g, 64% yield).

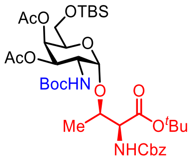

13b

***N*-Benzyloxycarbonyl-*O*-(3,4-di-*O*-acetyl-2-*tert*-butoxycarbonylamino-6-*O*-*tert*-butyldimethylsilyl-2-deoxy-α-D-galactopyranosyl)-L-threonine *tert*-butyl ester (13b)**: $[\alpha]_D^{22}$ +59.2 (CH_2Cl_2, c = 1.0); IR ν_{max} (neat)/cm^{-1}: 3343 (m), 2930 (w), 2360 (w), 1748 (m), 1705 (s), 1517 (m), 1456 (w), 1366 (m), 1310 (m), 1240 (s), 1220 (s), 1157 (s), 1094 (m), 1040 (s), 1005 (m), 947 (m), 922 (m), 837 (s), 776 (m), 756 (m); ^{1}H NMR (400 MHz, $CDCl_3$) 7.42 – 7.27 (m, 5H), 5.48 (d, J = 9.2 Hz, 1H), 5.40 (d, J = 3.5 Hz, 1H), 5.13 (s, 2H), 5.01 (dd, J = 11.2, 3.1 Hz, 1H), 4.94 (d, J = 3.7 Hz, 1H), 4.87 (d, J = 10.2 Hz, 1H), 4.30 – 4.07 (m, 3H), 4.03 (t, J = 6.7 Hz, 1H), 3.64 – 3.50 (m, 2H), 2.12 (s, 3H), 1.96 (s, 3H), 1.48 (s, 9H), 1.40 (s, 9H), 1.31 (d, J = 6.4 Hz, 3H), 0.85 (s, 9H), 0.01 (s, 3H), 0.00 (s, 3H); ^{13}C NMR (100 MHz, $CDCl_3$ δ 170.6, 170.2,

169.7, 156.4, 155.3, 136.1, 128.6 (2C), 128.2 (two peaks overlapped, 3C), 100.0, 83.1, 79.5, 76.8, 70.1, 69.8, 67.7, 67.3, 61.4, 58.7, 49.1, 28.3 (3C), 28.1 (3C), 25.8 (3C), 20.8, 20.7, 18.3, 18.2, -5.6, -5.7; HRMS: m/z (ESI) calcd for $C_{37}H_{61}N_2O_{13}Si^+$, $[M + H]^+$, 769.3937, found 769.3951. $^{1}J^{13}{}_{C1-H1}$ = 171.3Hz.

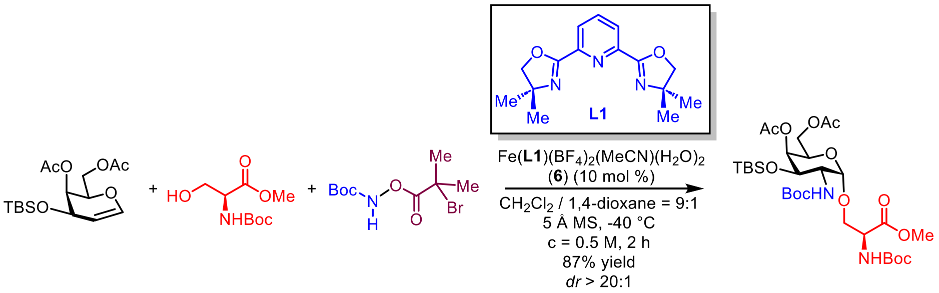

S8a was synthesized according to a literature procedure.[7]

To a flame-dried sealable 2-dram vial (vial **A**) equipped with a stir bar were added iron catalyst Fe(**L1**)$(BF_4)_2(MeCN)(H_2O)_2$ (**6**) (29.0 mg, 0.05 mmol, 10 mol %) and freshly activated 5 Å powdered molecular sieves (*ca.* 200 mg). After the vial was evacuated and backfilled with N_2 three times, the vial was cooled to -78 °C. To a second flame-dried sealable 2-dram vial (vial **B**) was added 4,6-di-*O*-acetyl-3-*O*-TBS-D-galactal (**2**) (172 mg, 0.50 mmol, 1.0 equiv) and *N*-Boc serine methyl ester (**S8a**) (154 mg, 0.70 mmol, 1.4 equiv). Vial **B** was evacuated and backfilled with N_2 three times, and then anhydrous CH_2Cl_2 (0.4 mL) and freshly distilled dioxane (0.1 ml) were added, then vial **B** solution was quickly transferred into vial **A** via a syringe drop wise within 1 min. To a third flame-dried sealable 2-dram vial (vial **C**) was added acyloxyl carbamate **3a** (212 mg, 0.75 mmol, 1.5 equiv). Vial **C** was evacuated and backfilled with N_2 three times, and then anhydrous CH_2Cl_2 (0.5 mL) was added. The solution in vial **C** was added to vial **A** via a syringe drop wise within 1 min. The reaction mixture was kept at -78 °C for 5 min and transferred to -40 °C for an additional 2 h, then quenched by imidazole (20.4 mg in 1 mL CH_2Cl_2) and diluted with Et_2O (4 mL) subsequently at the same temperature. The mixture was

stirred for 2 min at -40 °C and warmed up to room temperature. The solution was then filtered through a piece of cotton and washed with saturated $NaHCO_3$ solution (2 mL). The organic layer was separated from the aqueous one. The aqueous phase was further extracted with EtOAc (2 mL × 3). The combined organic layers were dried over anhydrous Na_2SO_4 and concentrated *in vacuo*. The residue was purified through a silica gel flash column (hexanes/ethyl acetate: from 20:1 to 3:1) to afford the desired product **14a** as white foam (295 mg, 87% yield).

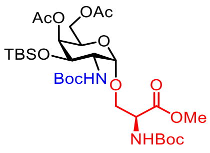

14a

***N*-*tert*-Butoxycarbonyl-*O*-(4,6-di-*O*-acetyl-2-*tert*-butoxycarbonylamino-3-*O*-*tert*-butyldimethylsilyl-2-deoxy-α-D-galactopyranosyl)-L-serine methyl ester** (**14a**): $[\alpha]_D^{22}$ +72.5 (acetone, *c* = 1.0); IR ν_{max} (neat)/cm^{-1}: 3378 (w), 2931 (w), 2857 (w), 2360 (w), 1747 (s), 1718 (s), 1505 (m), 1473 (w), 1366 (m), 1226 (s), 1165 (s), 1117 (m), 1060 (m), 1009 (w), 948 (w), 870 (w), 839 (m), 778 (m); ^{1}H NMR (400 MHz, $CDCl_3$) δ 5.43 (d, *J* = 8.5 Hz, 1H), 5.23 (d, *J* = 3.2 Hz, 1H), 4.78 (d, *J* = 3.4 Hz, 1H), 4.53 (d, *J* = 8.2 Hz, 1H), 4.38 (d, *J* = 10.3 Hz, 1H), 4.15 (dd, *J* = 11.1, 4.2 Hz, 1H), 4.10 – 3.94 (m, 3H), 3.93 – 3.82 (m, 2H), 3.79 (s, 3H), 3.71 (dd, *J* = 10.4, 3.1 Hz, 1H), 2.10 (s, 3H), 2.06 (s, 3H), 1.46 (s, 9H), 1.42 (s, 9H), 0.83 (s, 9H), 0.07 (s, 3H), 0.05 (s, 3H); ^{13}C NMR (100 MHz, $CDCl_3$) δ 170.8, 170.5, 170.3, 155.2, 155.0, 99.9, 80.3, 79.6, 69.83, 69.79, 68.2, 67.8, 62.7, 53.9, 52.6, 51.2, 28.4 (3C), 28.3 (3C), 25.5 (3C), 20.8, 20.7, 17.7, -4.8, -5.0; HRMS: m/z (ESI) calcd for $C_{30}H_{55}N_2O_{13}Si^+$, $[M + H]^+$, 679.3468, found 679.3483. $^1J_{^{13}C1\text{–}H1}$ = 170.4 Hz.

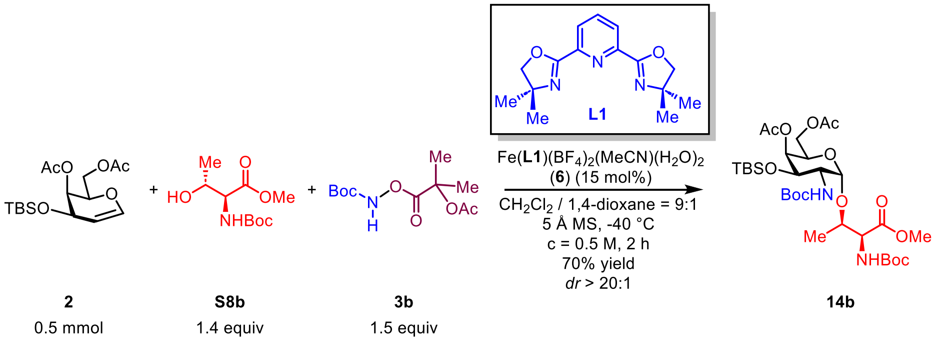

S8b was synthesized according to a literature procedure.[7]

To a flame-dried sealable 2-dram vial (vial **A**) equipped with a stir bar were added iron catalyst Fe(**L1**)$(BF_4)_2$(MeCN)$(H_2O)_2$ (**6**) (43.5 mg, 0.075 mmol, 15 mol %) and freshly activated 5 Å powdered molecular sieves (*ca.* 200 mg). After the vial was evacuated and backfilled with N_2 three times, the vial was cooled to -78 °C. To a second flame-dried sealable 2-dram vial (vial **B**) was added 4,6-di-*O*-acetyl-3-*O*-TBS-D-galactal (**2**) (172 mg, 0.50 mmol, 1.0 equiv) and *N*-Boc threonine methyl ester (**S8b**) (163 mg, 0.70 mmol, 1.4 equiv). Vial **B** was evacuated and backfilled with N_2 three times, and then anhydrous CH_2Cl_2 (0.4 mL) and freshly distilled dioxane (0.1 ml) were added, then vial **B** solution was quickly transferred into vial **A** via a syringe drop wise within 1 min. To a third flame-dried sealable 2-dram vial (vial **C**) was added acyloxyl carbamate **3b** (196 mg, 0.75 mmol, 1.5 equiv). Vial **C** was evacuated and backfilled with N_2 three times, and then anhydrous CH_2Cl_2 (0.5 mL) was added. The solution in vial **C** was added to vial **A** via a syringe drop wise within 1 min. The reaction mixture was kept at -78 °C for 5 min and transferred to -40 °C for an additional 2 h, then quenched by imidazole (20.4 mg in 1 mL CH_2Cl_2) and diluted with Et_2O (4 mL) subsequently at the same temperature. The mixture was stirred for 2 min at -40 °C and warmed up to room temperature. The solution was then filtered through a piece of cotton and washed with saturated $NaHCO_3$ solution (2 mL). The organic layer was separated from the aqueous one. The aqueous phase was further extracted with EtOAc (2 mL × 3). The combined organic layers were dried over anhydrous Na_2SO_4 and concentrated *in vacuo*. The residue was purified through a silica gel flash column (hexanes/ethyl acetate: from 20:1 to 3:1) to afford the desired product **14b** as white foam (243 mg, 70% yield).

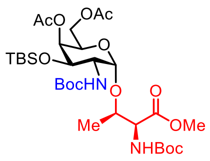

14b

***N*-*tert*-Butoxycarbonyl-*O*-(4,6-di-*O*-acetyl-2-*tert*-butoxycarbonylamino-3-*O*-*tert*-butyldimethylsilyl-2-deoxy-α-D-galactopyranosyl)-L-threonine methyl ester (14b)**: $[\alpha]_D^{22}$ +73.3 (acetone, *c* = 1.0); IR ν_{max} (neat)/cm^{-1}: 3419 (w), 2930 (w), 2857 (w), 2360 (w), 1747 (s), 1718 (s), 1506 (m), 1473 (w), 1457 (w), 1366 (m), 1306 (w), 1227 (s), 1166 (s), 1135 (m), 1093 (w), 1056 (m), 1008 (m), 946 (w), 838 (w), 778 (w); ^{1}H NMR (400 MHz, $CDCl_3$) δ 5.23 (d, *J* = 3.4 Hz, 1H), 5.15 (d, *J* = 9.5 Hz, 1H), 4.84 (d, *J* = 3.7 Hz, 1H), 4.46 (d, *J* = 10.4 Hz, 1H), 4.39 (dd, *J* = 9.4, 2.6 Hz, 1H), 4.24 – 4.09 (m, 3H), 4.09 – 3.93 (m, 2H), 3.80 (s, 3H), 3.70 (dd, *J* = 10.5, 3.4 Hz, 1H), 2.10 (s, 3H), 2.03 (s, 3H), 1.48 (s, 9H), 1.43 (s, 9H), 1.31 (d, *J* = 6.4 Hz, 3H), 0.83 (s, 9H), 0.09 (s, 3H), 0.07 (s, 3H); ^{13}C NMR (100 MHz, $CDCl_3$) δ 171.4, 170.5, 170.3, 155.8, 155.1, 100.9, 80.4, 79.5, 77.6, 70.0, 68.2, 67.8, 63.0, 57.9, 52.5, 51.4, 28.5 (3C), 28.3 (3C), 25.6 (3C), 20.8, 20.7, 17.7, 17.6, -4.8, -4.9; HRMS: m/z (ESI) calcd for $C_{31}H_{57}N_2O_{13}N^+$, [M + H]$^+$, 693.3624, found 693.3642. $^1J_{^{13}C1-H1}$ = 170.8 Hz.

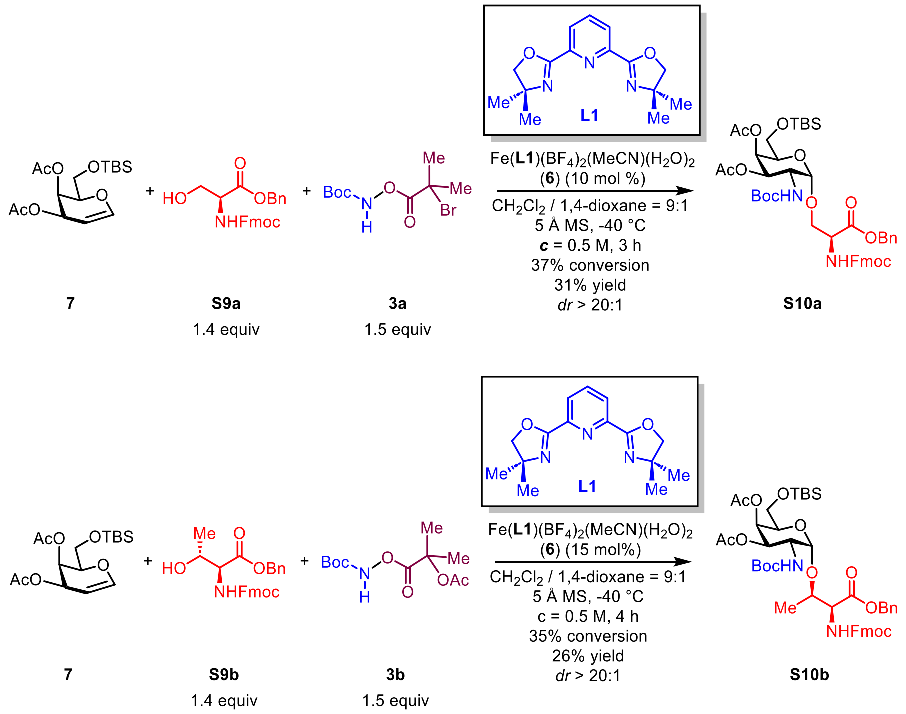

Figure S6. Iron-Catalyzed Galactal 1,2-*cis*-Aminoglycosylation with *N*-Fmoc Serine and Threonine Benzyl Esters.

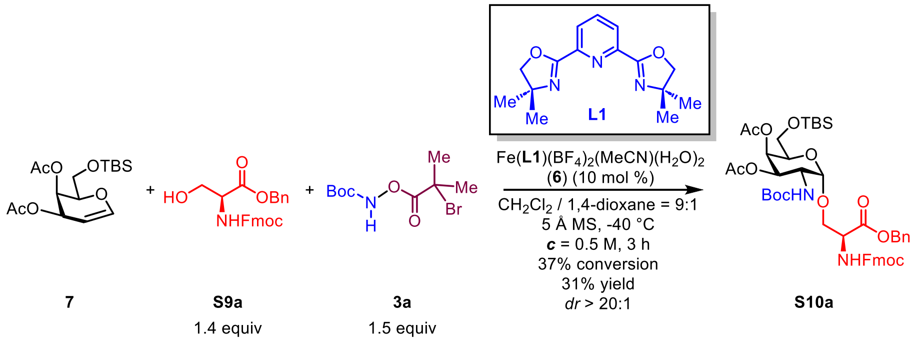

S9a was synthesized according to a literature procedure.[8]

To a flame-dried sealable 2-dram vial (vial **A**) equipped with a stir bar were added iron catalyst Fe(**L1**)$(BF_4)_2$(MeCN)$(H_2O)_2$ (**6**) (29.0 mg, 0.05 mmol, 10 mol %) and freshly activated 5 Å powdered molecular sieves (*ca.* 200 mg). After the vial was evacuated and backfilled with N_2 three times, the vial was cooled to -78 °C. To a second flame-dried sealable 2-dram vial (vial **B**)

was added 3,4-di-*O*-acetyl-6-*O*-TBS-D-galactal (**7**) (172 mg, 0.50 mmol, 1.0 equiv) and *N*-Fmoc serine benzyl ester (**S9a**) (292 mg, 0.70 mmol, 1.4 equiv). Vial **B** was evacuated and backfilled with N_2 three times, and then anhydrous CH_2Cl_2 (0.4 mL) and freshly distilled dioxane (0.1 ml) were added, then vial **B** solution was quickly transferred into vial **A** via a syringe drop wise within 1 min. To a third flame-dried sealable 2-dram vial (vial **C**) was added acyloxyl carbamate **3a** (212 mg, 0.75 mmol, 1.5 equiv). Vial **C** was evacuated and backfilled with N_2 three times, and then anhydrous CH_2Cl_2 (0.5 mL) was added. The solution in vial **C** was added to vial **A** via a syringe drop wise within 1 min. The reaction mixture was kept at -78 °C for 5 min and transferred to -40 °C for an additional 3 h, then quenched by imidazole (20.4 mg in 1 mL CH_2Cl_2) and diluted with Et_2O (4 mL) subsequently at the same temperature. The mixture was stirred for 2 min at -40 °C and warmed up to room temperature. The solution was then filtered through a piece of cotton and washed with saturated $NaHCO_3$ solution (2 mL). The organic layer was separated from the aqueous one. The aqueous phase was further extracted with EtOAc (2 mL × 3). The combined organic layers were dried over anhydrous Na_2SO_4 and concentrated *in vacuo*. The residue was purified through a silica gel flash column (hexanes/diethyl ether: from 20:1 to 3:2) to afford the desired product **S10a** as white foam (136 mg, 31% yield).

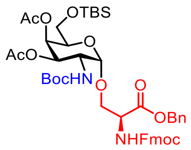

S10a

***N*-Fluorenylmethyloxycarbonyl-*O*-(3,4-di-*O*-acetyl-2-*tert*-butoxycarbonylamino-6-*O*-*tert*-butyldimethylsilyl-2-deoxy-α-D-galactopyranosyl)-L-serine benzyl ester** (**S10a**): $[\alpha]_D^{22}$ +32.7 (acetone, c = 1.0); IR ν_{max} (neat)/cm^{-1}: 3348 (w), 2929 (w), 2857 (w), 2360 (w), 2341 (w), 2157 (w), 2008 (w), 1749 (s), 1718 (s), 1507 (m), 1472 (w), 1456 (m), 1367 (m), 1242 (s), 1219 (s), 1169 (m), 1143 (m), 1109 (m), 1044 (m), 1006 (m), 949 (w), 838 (m), 759 (w), 740 (m); ^{1}H NMR (400 MHz, $CDCl_3$) δ 7.77 (d, J = 7.5 Hz, 2H), 7.62 (d, J = 7.5 Hz, 2H), 7.43 – 7.29 (m, 9H), 5.81 (d, J = 8.3 Hz, 1H), 5.42 (s, 1H), 5.21 (ABq, $\Delta\nu_{AB}$ = 11.8 Hz, J_{AB} = 12.0 Hz, 2H), 5.01 (d, J = 12.0 Hz, 1H), 4.78 (d, J = 2.7 Hz, 1H), 4.66 – 4.54 (m, 2H), 4.47 – 4.33 (m, 2H), 4.29 – 4.17 (m, 2H), 4.02 – 3.84 (m, 3H), 3.63 (dd, J = 10.0, 6.4 Hz, 1H), 3.54 (dd, J = 9.9, 7.2 Hz, 1H),

2.14 (s, 3H), 2.00 (s, 3H), 1.41 (s, 9H), 0.84 (s, 9H), 0.01 (s, 3H), -0.01 (s, 3H); ^{13}C NMR (100 MHz, $CDCl_3$) δ 170.5, 170.0, 169.8, 155.8, 155.2, 143.8, 143.7, 141.3 (2C), 134.3, 128.8 (two peaks overlapped, 3C), 128.5 (2C), 127.7 (2C), 127.1 (2C), 125.2 (2C), 120.0 (2C), 99.8, 79.9, 69.90, 69.86, 69.3, 67.7, 67.4, 67.3, 61.0, 54.4, 49.0, 47.1, 28.3 (3C), 25.7 (3C), 20.8, 20.7, 18.2, -5.6, -5.7; HRMS: m/z (ESI) calcd for $C_{46}H_{61}N_2O_{13}Si^+$, $[M + H]^+$, 877.3937, found 877.3951. $^1J_{^{13}C1\text{–}H1}$ = 173.2Hz.

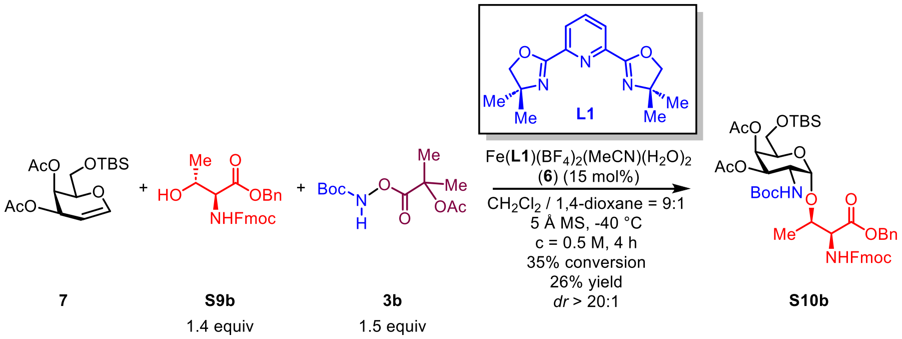

S9b was synthesized according to a literature procedure.[8]

To a flame-dried sealable 2-dram vial (vial **A**) equipped with a stir bar were added iron catalyst Fe(**L1**)$(BF_4)_2(MeCN)(H_2O)_2$ **6** (43.5 mg, 0.075 mmol, 15 mol %) and freshly activated 5 Å powdered molecular sieves (*ca.* 200 mg). After the vial was evacuated and backfilled with N_2 three times, the vial was cooled to -78 °C. To a second flame-dried sealable 2-dram vial (vial **B**) was added 3,4-di-*O*-acetyl-6-*O*-TBS-D-galactal (**7**) (172 mg, 0.50 mmol, 1.0 equiv) and *N*-Fmoc threonine benzyl ester (**S9b**) (302 mg, 0.70 mmol, 1.4 equiv). Vial **B** was evacuated and backfilled with N_2 three times, and then anhydrous CH_2Cl_2 (0.4 mL) and freshly distilled dioxane (0.1 ml) were added, then vial **B** solution was quickly transferred into vial **A** via a syringe drop wise within 1 min. To a third flame-dried sealable 2-dram vial (vial **C**) was added acyloxyl carbamate **3b** (196 mg, 0.75 mmol, 1.5 equiv). Vial **C** was evacuated and backfilled with N_2 three times, and then anhydrous CH_2Cl_2 (0.5 mL) was added. The solution in vial **C** was added to vial **A** via a syringe drop wise within 1 min. The reaction mixture was kept at -78 °C for 5 min and transferred to -40 °C for an additional 4 h, then quenched by imidazole (20.4 mg in 1 mL CH_2Cl_2) and diluted with Et_2O (4 mL) subsequently at the same temperature. The mixture

was stirred for 2 min at -40 °C and warmed up to room temperature. The solution was then filtered through a piece of cotton and washed with saturated $NaHCO_3$ solution (2 mL). The organic layer was separated from the aqueous one. The aqueous phase was further extracted with EtOAc (2 mL × 3). The combined organic layers were dried over anhydrous Na_2SO_4 and concentrated *in vacuo*. The residue was purified through a silica gel flash column (hexanes/diethyl ether: from 20:1 to 3:2) to afford the desired product **S10b** as white foam (116 mg, 26% yield).

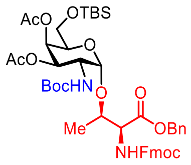

S10b

***N*-Fluorenylmethyloxycarbonyl-*O*-(3,4-di-*O*-acetyl-2-*tert*-butoxycarbonylamino-6-*O*-*tert*-butyldimethylsilyl-2-deoxy-α-D-galactopyranosyl)-L-threonine benzyl ester (S10b)**: $[\alpha]_D^{22}$ +24.6 (acetone, *c* = 1.0); IR ν_{max} (neat)/cm^{-1}: 3347 (w), 2930 (w), 2857 (w), 2360 (w), 2341 (w), 2160 (w), 2007 (w), 1750 (s), 1719 (s), 1507 (m), 1472 (w), 1452 (w), 1247 (s), 1220 (s), 1169 (m), 1136 (m), 1107 (m), 1042 (m), 1007 (m), 947 (w), 839 (m), 778 (m), 759 (m), 741 (m); ^{1}H NMR (400 MHz, $CDCl_3$) δ 7.78 (d, *J* = 7.5 Hz, 2H), 7.64 (d, *J* = 7.5 Hz, 2H), 7.45 – 7.30 (m, 9H), 5.55 (d, *J* = 9.5 Hz, 1H), 5.45 (d, *J* = 3.1 Hz, 1H), 5.27 – 5.16 (ABq, $\Delta\nu_{AB}$ = 8.7 Hz, J_{AB} = 12.4 Hz, 2H), 5.02 (dd, *J* = 11.5, 3.0 Hz, 1H), 4.84 (d, *J* = 3.8 Hz, 1H), 4.73 (d, *J* = 10.3 Hz, 1H), 4.50 (dd, *J* = 9.5, 2.5 Hz, 1H), 4.45 – 4.39 (m, 2H), 4.28 – 4.12 (m, 3H), 4.04 (t, *J* = 6.7 Hz, 1H), 3.65 – 3.52 (m, 2H), 2.15 (s, 3H), 2.00 (s, 3H), 1.43 (s, 9H), 1.31 (d, *J* = 6.3 Hz, 3H), 0.86 (s, 9H), 0.02 (s, 3H), 0.01 (s, 3H); ^{13}C NMR (100 MHz, $CDCl_3$) δ 170.6, 170.5, 170.1, 156.5, 155.3, 143.8, 143.7, 141.3 (2C), 134.8, 128.8 (two peaks overlapped, 3C), 128.7 (2C), 127.7 (2C), 127.1(2C), 125.21, 125.17, 120.0, 119.97, 100.6, 79.8, 77.6, 70.0, 69.6, 67.8, 67.57, 67.56, 61.3, 58.5, 49.1, 47.1, 28.3 (3C), 25.8 (3C), 20.8, 20.7, 18.2, 18.0, -5.6, -5.7; HRMS: m/z (ESI) calcd for $C_{47}H_{63}N_2O_{13}Si^+$, [M + H]$^+$, 891.4094, found 891.4071. $^1J_{^{13}C1-H1}$ = 178.0 Hz.

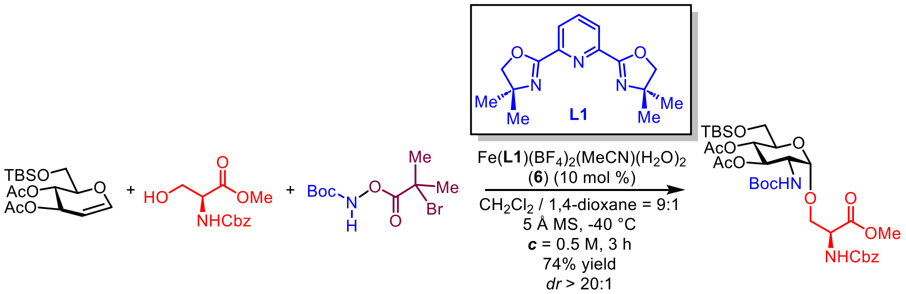

S11 **4a** 1.4 equiv **3a** 1.5 equiv **S12a**

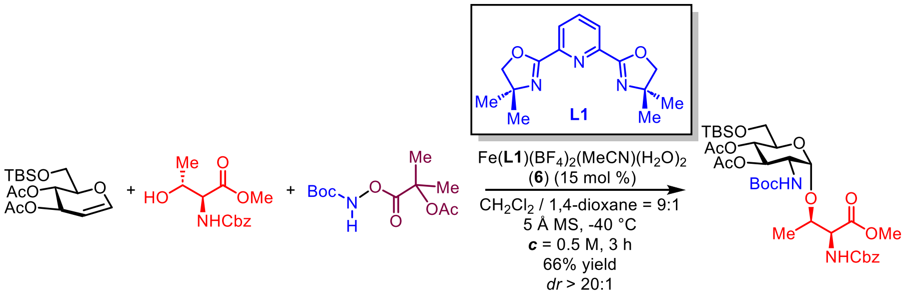

S11 **4b** 1.4 equiv **3b** 1.5 equiv **S12b**

Figure S7. Iron-Catalyzed1,2-*cis*-Aminoglycosylation of 3,4-di-*O*-Acetyl-6-*O*-TBS-D-Glucal **S11** with *N*-Cbz Serine and Threonine Methyl Esters.

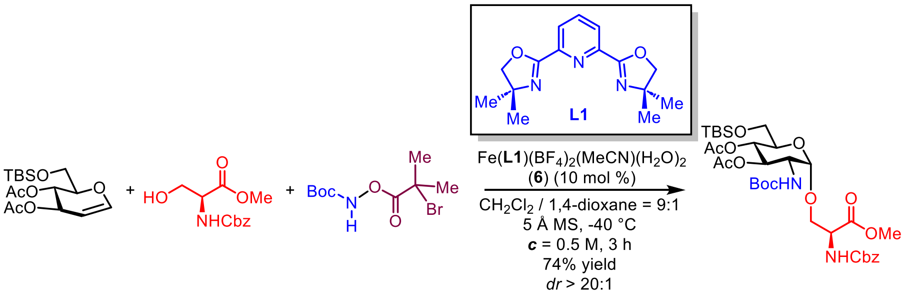

S11 **4a** 1.4 equiv **3a** 1.5 equiv **S12a**

S11 was synthesized according to a literature procedure.[9]

To a flame-dried sealable 2-dram vial (vial **A**) equipped with a stir bar were added iron catalyst Fe(**L1**)$(BF_4)_2$(MeCN)$(H_2O)_2$ (**6**) (29.0 mg, 0.05 mmol, 10 mol %) and freshly activated 5 Å powdered molecular sieves (*ca.* 200 mg). After the vial was evacuated and backfilled with N_2 three times, the vial was cooled to -78 °C. To a second flame-dried sealable 2-dram vial (vial **B**)

was added 3,4-di-*O*-acetyl-6-*O*-TBS-D-glucal (**S11**) (172 mg, 0.50 mmol, 1.0 equiv) and *N*-Cbz serine methyl ester (**4a**) (177 mg, 0.70 mmol, 1.4 equiv). Vial **B** was evacuated and backfilled with N_2 three times, and then anhydrous CH_2Cl_2 (0.4 mL) and freshly distilled dioxane (0.1 ml) were added, then vial **B** solution was quickly transferred into vial **A** via a syringe drop wise within 1 min. To a third flame-dried sealable 2-dram vial (vial **C**) was added acyloxyl carbamate **3a** (212 mg, 0.75 mmol, 1.5 equiv). Vial **C** was evacuated and backfilled with N_2 three times, and then anhydrous CH_2Cl_2 (0.5 mL) was added. The solution in vial **C** was added to vial **A** via a syringe drop wise within 1 min. The reaction mixture was kept at -78 °C for 5 min and transferred to -40 °C for an additional 3 h, then quenched by imidazole (20.4 mg in 1 mL CH_2Cl_2) and diluted with Et_2O (4 mL) subsequently at the same temperature. The mixture was stirred for 2 min at -40 °C and warmed up to room temperature. The solution was then filtered through a piece of cotton and washed with saturated $NaHCO_3$ solution (2 mL). The organic layer was separated from the aqueous one. The aqueous phase was further extracted with EtOAc (2 mL × 3). The combined organic layers were dried over anhydrous Na_2SO_4 and concentrated *in vacuo*. The residue was purified through a silica gel flash column (hexanes/ethyl acetate: from 20:1 to 4:1) to afford the desired product **S12a** as white foam (264 mg, 74% yield).

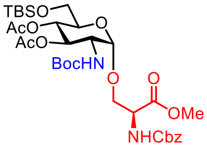

S12a

***N*-Benzyloxycarbonyl-*O*-(3,4-di-*O*-acetyl-2-*tert*-butoxycarbonylamino-6-*O*-*tert*-butyldimethylsilyl-2-deoxy-α-D-glucopyranosyl)-L-serine methyl ester** (**S12a**): $[\alpha]_D^{22}$ +80.6 (CH_2Cl_2, c = 1.0); IR ν_{max} (neat)/cm^{-1}: 3446 (m), 3016 (m), 2970 (m), 2359 (w), 1738 (s), 1728 (s), 1506 (w), 1435 (m), 1366 (s), 1229 (s), 1217 (s), 1092 (w), 1038 (m), 1011 (m), 834 (m), 776 (m); ^{1}H NMR (400 MHz, $CDCl_3$) δ 7.45 – 7.28 (m, 5H), 5.73 (d, J = 8.7 Hz, 1H), 5.14 (s, 2H), 5.11 –4.99 (m, 2H), 4.78 (d, J = 3.7 Hz, 1H), 4.63 (d, J = 10.1 Hz, 1H), 4.60 – 4.53 (m, 1H), 3.97 – 3.88 (m, 3H), 3.78 (s, 3H), 3.75 – 3.69 (m, 1H), 3.68 – 3.58 (m, 2H), 2.00 (s, 3H), 1.99 (s, 3H), 1.40 (s, 9H), 0.86 (s, 9H), 0.01 (s, 3H), 0.00 (s, 3H); ^{13}C NMR (100 MHz, $CDCl_3$) δ 171.0, 170.4, 169.2, 155.8, 155.0, 136.0, 128.5 (2C), 128.2, 128.1 (2C), 98.9, 79.9, 71.8, 70.9,

69.5, 68.5, 67.2, 61.9, 54.2, 52.9, 52.7, 28.2 (3C), 25.8 (3C), 20.69, 20.65, 18.2, -5.48, -5.52; HRMS: m/z (ESI) calcd for $C_{33}H_{53}N_2O_{13}Si^+$, $[M + H]^+$, 713.3311, found 713.3315. $^1J_{^{13}C1\text{–}H1}$ = 173.8 Hz.

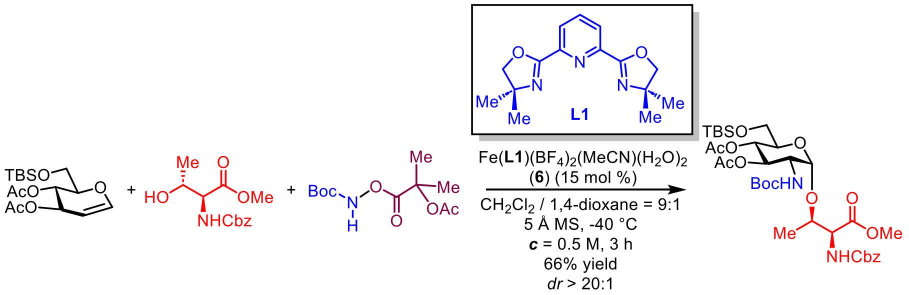

S11 4b 1.4 equiv 3b 1.5 equiv S12b

To a flame-dried sealable 2-dram vial (vial **A**) equipped with a stir bar were added iron catalyst Fe(**L1**)$(BF_4)_2(MeCN)(H_2O)_2$ **6** (43.5 mg, 0.075 mmol, 15 mol %) and freshly activated 5 Å powdered molecular sieves (*ca.* 200 mg). After the vial was evacuated and backfilled with N_2 three times, the vial was cooled to -78 °C. To a second flame-dried sealable 2-dram vial (vial **B**) was added 3,4-di-*O*-acetyl-6-*O*-TBS-D-glucal (**S11**) (172 mg, 0.50 mmol, 1.0 equiv) and *N*-Cbz threonine methyl ester (**4b**) (187 mg, 0.70 mmol, 1.4 equiv). Vial **B** was evacuated and backfilled with N_2 three times, and then anhydrous CH_2Cl_2 (0.4 mL) and freshly distilled dioxane (0.1 ml) were added, then vial **B** solution was quickly transferred into vial **A** via a syringe drop wise within 1 min. To a third flame-dried sealable 2-dram vial (vial **C**) was added acyloxyl carbamate **3b** (196 mg, 0.75 mmol, 1.5 equiv). Vial **C** was evacuated and backfilled with N_2 three times, and then anhydrous CH_2Cl_2 (0.5 mL) was added. The solution in vial **C** was added to vial **A** via a syringe drop wise within 1 min. The reaction mixture was kept at -78 °C for 5 min and transferred to -40 °C for an additional 4 h, then quenched by imidazole (20.4 mg in 1 mL CH_2Cl_2) and diluted with Et_2O (4 mL) subsequently at the same temperature. The mixture was stirred for 2 min at -40 °C and warmed up to room temperature. The solution was then filtered through a piece of cotton and washed with saturated $NaHCO_3$ solution (2 mL). The organic layer was separated from the aqueous one. The aqueous phase was further extracted with EtOAc (2 mL × 3). The combined organic layers were dried over anhydrous Na_2SO_4 and

concentrated *in vacuo*. The residue was purified through a silica gel flash column (hexanes/ethyl acetate: from 20:1 to 4:1) to afford the desired product **S12b** as white foam (240 mg, 66% yield).

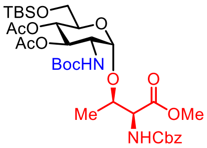

S12b

***N*-Benzyloxycarbonyl-*O*-(3,4-di-*O*-acetyl-2-*tert*-butoxycarbonylamino-6-*O*-*tert*-butyldimethylsilyl-2-deoxy-α-D-glucopyranosyl)-L-threonine methyl ester (S12b)**: $[\alpha]_D^{22}$ +70.7 (CH_2Cl_2, *c* = 1.0); IR ν_{max} (neat)/cm^{-1}: 3343 (w), 2956 (w), 2933 (w), 2863 (w), 2362 (w), 2343 (w), 1751 (s), 1718 (s), 1509 (m), 1366 (m), 1238 (s), 1171 (m), 1125 (m), 1039 (s), 1011 (s), 837 (m); ^{1}H NMR (400 MHz, $CDCl_3$) δ 7.43 – 7.29 (m, 5H), 5.51 (d, *J* = 9.8 Hz, 1H), 5.15 (s, 2H), 5.06 (t, *J* = 10.1 Hz, 1H), 4.96 (t, *J* = 9.8 Hz, 1H), 4.84 (d, *J* = 3.9 Hz, 1H), 4.66 (d, *J* = 10.4 Hz, 1H), 4.45 (dd, *J* = 9.6, 2.6 Hz, 1H), 4.31 – 4.21 (m, 1H), 3. 97 – 3.80 (m, 2 H), 3.77 (s, 3H), 3.67 – 3.57 (m, 2H), 2.00 (s, 3H), 1.99 (s, 3H), 1.40 (s, 9H), 1.34 (d, *J* = 6.5 Hz, 3H), 0.86 (s, 9H), 0.02 (s, 6H). ^{13}C NMR (100 MHz, $CDCl_3$) δ 170.9 (two peaks overlapped, 2C), 169.3, 156.4, 155.1, 136.0, 128.5 (2C), 128.24, 128.17 (2C), 99.7, 79.8, 77.6, 71.9, 71.1, 68.9, 67.3, 62.4, 58.4, 53.0, 52.6, 28.2 (3C), 25.8 (3C), 20.68, 20.66, 18.3, 18.0, -5.5 (two peaks overlapped, 2C); HRMS: m/z (ESI) calcd for $C_{34}H_{55}N_2O_{13}Si^+$, $[M + H]^+$, 727.3468, found 727.3479. $^1J_{^{13}C1-H1}$ = 171.6 Hz.

D. Procedures for Post-glycosylation Transformations to Afford Tn Antigens and *O*-Galactosyl Amino Acids

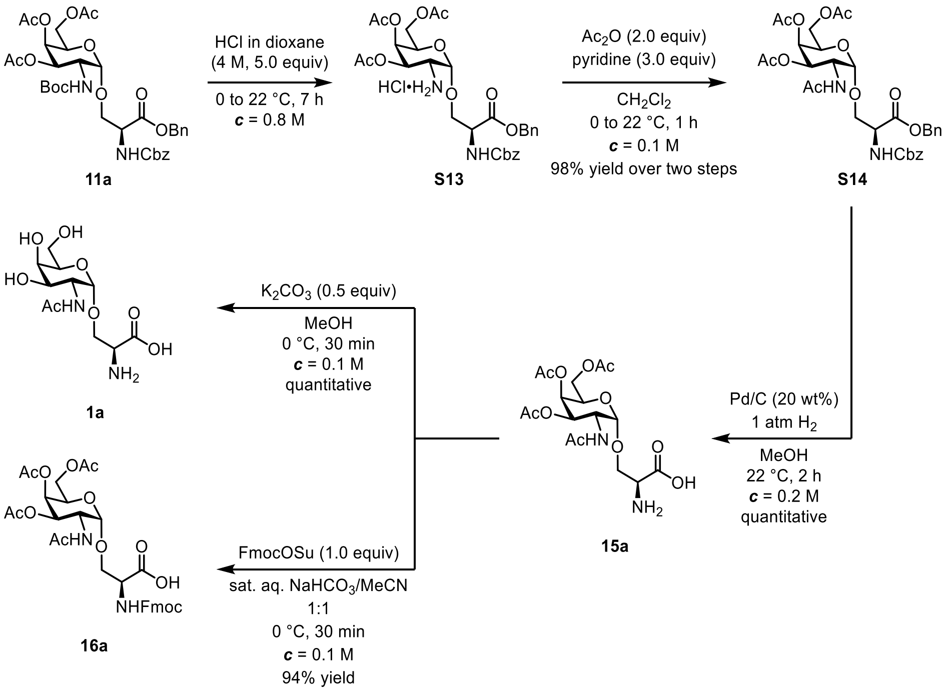

Figure S8. Post-glycosylation Transformations to Afford Tn Antigen **1a** and *O*-Galactosyl Amino Acid **16a**.

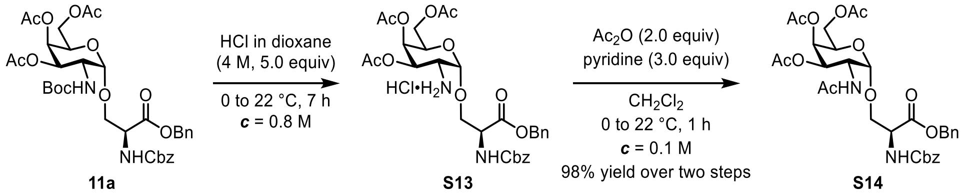

To a 100 mL oven-dried round-bottom flask equipped with a magnetic stirring bar was added compound **11a** (4 g, 5.58 mmol, 1.0 equiv). The vial was cooled to 0 °C, followed by addition of HCl (4 M solution in 1,4-dioxane, 6.98 mL, 27.90 mmol, 5.0 equiv). The reaction mixture was

stirred for 10 min at 0 °C before being warmed to room temperature. The reaction mixture was stirred for another 7 h with the progress monitored by TLC until completion. The reaction mixture was concentrated *in vacuo* with a base tube to afford the desired product **S13** as white foam which was directly used in the next step without further purification.

To the same flask containing the crude product (5.58 mmol, 1.0 equiv) from the previous step were added anhydrous CH_2Cl_2 (56 mL) and pyridine (1.35 mL, 16.74 mmol, 3.0 equiv). After the mixture was stirred for 3 min at 0 °C, Ac_2O (1.06 mL, 11.16 mmol, 2.0 equiv) was added dropwise. The reaction mixture was slowly warmed to room temperature and stirred for 1 h with the progress monitored by TLC until completion. The reaction mixture was then quenched with saturated aqueous NH_4Cl solution (32 mL). The organic phase was separated from the aqueous one, which was further extracted with CH_2Cl_2 (40 mL × 3). The combined organic layer was washed with brine, dried over anhydrous Na_2SO_4, and concentrated *in vacuo.* The desired product **S14** was obtained through column chromatography (hexanes/acetone: from 20:1 to 3:2) as white foam (3.6 g, 98% yield over two steps).

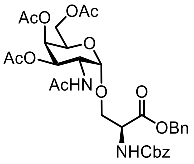

S14

***N*-Benzyloxycarbonyl-*O*-(2-acetamido-3,4,6-tri-*O*-acetyl-2-deoxy-α-D-galactopyranosyl)-L-serine benzyl ester** (**S14**): $[\alpha]_D^{22}$ +77.2 (acetone, *c* = 1.0); IR ν_{max} (neat)/cm^{-1}: 3355 (w), 2970 (w), 2360 (w), 1746 (s), 1522 (w), 1372 (w), 1230 (s), 1050 (m); ^{1}H NMR (400 MHz, $CDCl_3$) δ 7.34 (m, 10H), 5.82 (d, *J* = 8.3 Hz, 1H), 5.60 (d, *J* = 9.5 Hz, 1H), 5.30 (d, *J* = 3.2 Hz, 1H), 5.18 (ABq, $\Delta\nu_{AB}$ = 12.0 Hz, J_{AB} = 11.9 Hz, 2H), 5.12 (s, 2H), 5.02 (dd, *J* = 11.4, 3.2 Hz, 1H), 4.78 (d, *J* = 3.7 Hz, 1H), 4.60 (d, *J* = 8.3 Hz, 1H), 4.51 (ddd, *J* = 11.3, 9.5, 3.7 Hz, 1H), 4.04 (m, 3H), 3.97 – 3.86 (m, 2H), 2.14 (s, 3H), 1.99 (s, 3H), 1.98 (s, 3H), 1.89 (s, 3H); ^{13}C NMR (100 MHz, $CDCl_3$) δ 170.8, 170.4, 170.2, 170.1, 169.9, 155.8, 135.9, 134.7, 128.82 (2C), 128.80 (2C), 128.6 (2C), 128.4 (2C), 128.3, 128.2, 99.0, 69.7, 68.2, 67.7, 67.3, 67.2, 67.1, 61.9, 54.5, 47.6, 23.1,

20.70, 20.67, 20.6; HRMS: m/z (ESI) calcd for $C_{32}H_{39}N_2O_{13}^+$, $[M + H]^+$, 659.2447, found 659.2429. $^1J^{13}{}_{C1–H1}$ = 175.5 Hz.

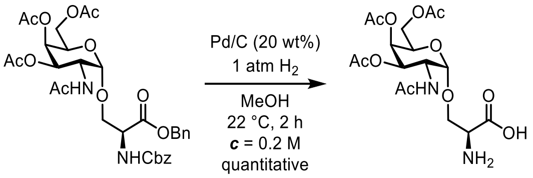

S14 **15a**

To a 100 mL round-bottom flask equipped with a magnetic stirring bar was added compound **S14** (3.6 g, 5.46 mmol, 1.0 equiv). After the flask was evacuated and backfilled with N_2 three times, Pd/C (720 mg, 20 wt %) was added. The flask was once again evacuated and backfilled with N_2 three times before MeOH (27.4 mL) was added. The flask was then evacuated and backfilled with H_2 three times and a H_2 atmosphere (1 atm) was maintained with a hydrogen balloon. The solution was stirred for 2 h at room temperature, with progress monitored by TLC until completion. The reaction mixture was then filtered through a short pad of Celite® (washed with MeOH), and the filtrate was concentrated *in vacuo* to afford the desired product **15a** (2.38 g, quantitative yield) as a white solid.

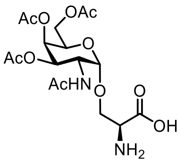

15a

***O*-(2-Acetamido-3,4,6-tri-*O*-acetyl-2-deoxy-α-D-galactopyranosyl)-L-serine** (**15a**): $[\alpha]_D^{22}$ +89.9 (methanol, *c* = 1.0); IR ν_{max} (neat)/cm^{-1}: 2970 (w), 2583 (w), 2360 (s), 2342 (m), 1792 (s), 1559 (s), 1374 (m), 1229 (s), 1053 (m); ^{1}H NMR (400 MHz, CD_3OD) δ 5.42 (d, *J* = 2.5 Hz, 1H), 5.20 (dd, *J* = 11.5, 3.2 Hz, 1H), 4.93 (d, *J* = 3.6 Hz, 1H), 4.50 (dd, *J* = 11.5, 3.6 Hz, 1H), 4.38 – 4.25 (m, 1H), 4.18 (dd, *J* = 11.1, 6.5 Hz, 1H), 4.15 – 4.04 (m, 2H), 3.92 – 3.82 (m, 2H), 2.15 (s, 3H), 2.04 (s, 3H), 1.98 (s, 3H), 1.95 (s, 3H); ^{13}C NMR (100 MHz, CD_3OD) δ 173.6, 172.2, 172.1,

172.0, 171.2, 100.1, 69.7, 68.6, 68.4, 68.3, 62.8, 55.9, 48.7, 22.8, 20.63, 20.59, 20.5; HRMS: m/z (ESI) calcd for $C_{17}H_{27}N_2O_{11}^+$, $[M + H]^+$, 435.1609, found 435.1601. $^1J^{13}{}_{C1–H1}$ = 172.2 Hz.

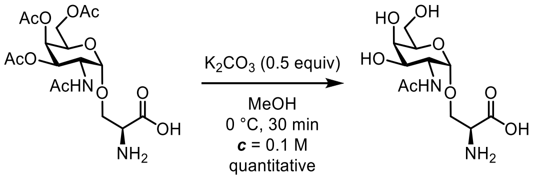

15a **1a**

To a 250 mL round-bottom flask equipped with a magnetic stirring bar were added compound **15a** (2.38 g, 5.46 mmol, 1.0 equiv) and MeOH (54.6 mL). The solution was cooled to 0 °C, and K_2CO_3 (powder, 378 mg, 2.73 mmol, 0.5 equiv) was then added. The reaction mixture was stirred for 30 min at 0 °C, with progress monitored by TLC until completion. The reaction mixture was then diluted with MeOH (40 mL) and neutralized with Amberlite® IRC 120 H. The reaction mixture was then filtered through a piece of cotton (washed with MeOH), and the filtrate was concentrated *in vacuo* to afford the desired product **1a** (1.68 g, quantitative yield) as a white solid (m.p. 199–201 °C).

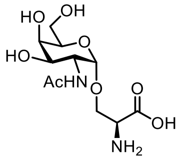

1a

***O*-(2-Acetamido-2-deoxy-α-D-galactopyranosyl)-L-serine** (**1a**): $[\alpha]_D^{22}$ +194.8 (methanol, *c* = 0.36); IR ν_{max} (neat)/cm^{-1}: 3283 (m), 2926 (m), 2569 (w), 2360 (s), 2342 (m), 1734 (m), 1700 (s), 1617 (m), 1374 (m), 1229 (m), 1053 (m); ^{1}H NMR (400 MHz, CD_3OD) δ 4.82 (d, *J* = 3.7 Hz, 1H), 4.32 (dd, *J* = 11.0, 3.7 Hz, 1H), 4.07 (dd, *J* = 10.9, 3.1 Hz, 1H), 3.92 – 3.81 (m, 3H), 3.78 (m, 3H), 3.70 (dd, *J* = 11.3, 4.6 Hz, 1H), 2.02 (s, 3H); ^{13}C NMR (100 MHz, CD_3OD) δ 174.1, 172.1, 100.2, 73.1, 70.4, 69.7, 68.6, 63.0, 56.2, 51.2, 22.9; HRMS: m/z (ESI) calcd for $C_{11}H_{21}N_2O_8^+$, $[M + H]^+$, 309.1292, found 309.1289. $^1J^{13}{}_{C1–H1}$ = 170.7 Hz.

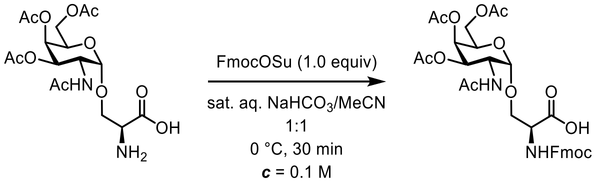

15a **16a**

To a 250 mL round-bottom flask equipped with a magnetic stirring bar were added compound **15a** (2.38 g, 5.46 mmol, 1.0 equiv) and saturated aqueous $NaHCO_3$ (27.4 mL). The solution was cooled to 0 °C, and a precooled solution of FmocOSu (1.84 g, 5.46 mmol, 1.0 equiv) in MeCN (27.4 mL) was then added dropwise. The reaction mixture was stirred for 30 min at 0 °C, with progress monitored by TLC until completion. MeCN was then removed *in vacuo* and the remaining solution was extracted with Et_2O (30 mL × 2). The aqueous phase was acidified with aqueous HCl solution (1 M) at 0 °C until pH reaches 2. EtOAc (60 mL × 3) was then used to extract the aqueous phase. The combined organic layer was washed with brine, dried over anhydrous Na_2SO_4, and concentrated *in vacuo* to afford the desired product **16a** (3.38 g, 94% yield) as a white solid (m.p. 130–132 °C).

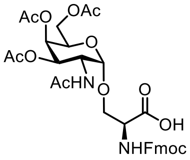

16a

***N*-Fluorenylmethyloxycarbonyl-*O*-(2-acetamido-3,4,6-tri-*O*-acetyl-2-deoxy-α-D-galactopyranosyl)-L-serine** (**16a**): $[\alpha]_D^{22}$ +92.3 (acetone, *c* = 1.0); IR ν_{max} (neat)/cm^{-1}: 3356 (w), 2970 (w), 2360 (w), 2341 (w), 1748 (s), 1540(w), 1372 (w), 1229 (s), 1052 (m); ^1H NMR (400 MHz, acetone-d_6) δ 7.87 (d, *J* = 7.5 Hz, 2H), 7.70 (dd, *J* = 7.5, 3.8 Hz, 2H), 7.42 (t, *J* = 7.5 Hz, 2H), 7.34 (td, *J* = 7.5, 3.8 Hz, 2H), 5.39 (dd, *J* = 3.3, 1.4 Hz, 1H), 5.12 (dd, *J* = 11.6, 3.3 Hz, 1H), 4.98 (d, *J* = 3.3 Hz, 1H), 4.52 (t, *J* = 3.5 Hz, 1H), 4.44 (dd, *J* = 11.6, 3.5 Hz, 1H), 4.41 – 4.33 (m, 3H), 4.26 (t, *J* = 7.0 Hz, 1H), 4.13 (dd, *J* = 11.2, 6.0 Hz, 1H), 4.10 – 3.99 (m, 3H), 2.12 (s, 3H),

1.95 (s, 3H), 1.90 (s, 3H), 1.88 (s, 3H); ^{13}C NMR (100 MHz, acetone-d$_6$) δ 171.9, 170.8, 170.7, 170.6, 170.4, 157.0, 145.0 (2C), 142.1 (2C), 128.6 (2C), 128.0 (2C), 126.1 (2C), 120.8 (2C), 99.8, 69.7, 68.9, 68.2, 67.9, 67.2, 62.6, 55.2, 48.3, 48.0, 22.9, 20.7, 20.62, 20.60; HRMS: m/z (ESI) calcd for $C_{32}H_{37}N_2O_{13}^+$, $[M + H]^+$, 657.2290, found 657.2280. $^{1}J_{^{13}C1\text{–}H1}$ = 175.4 Hz.

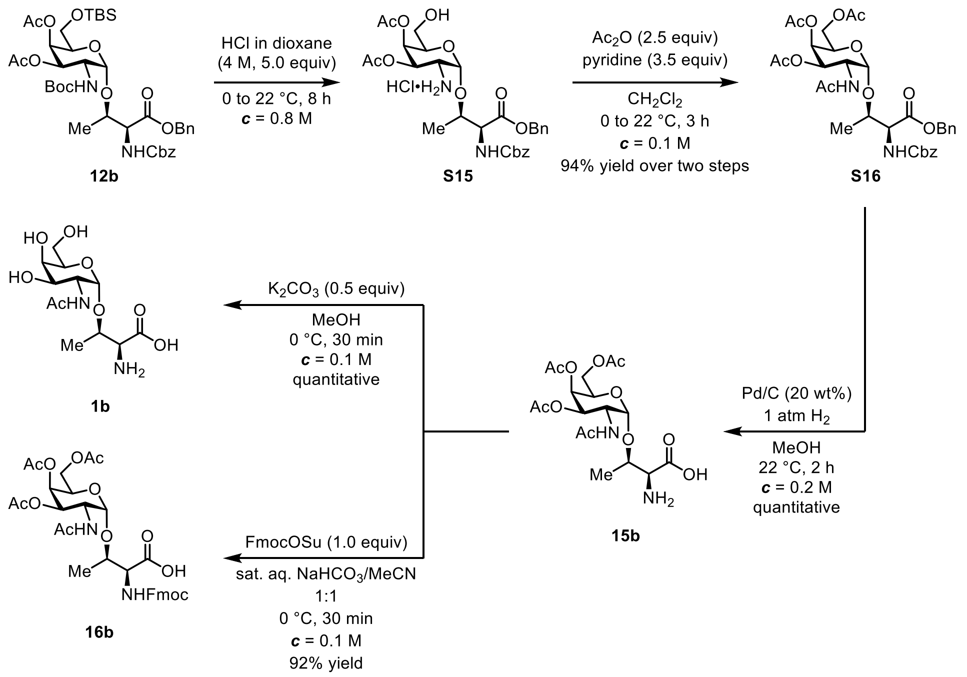

Figure S9. Post-glycosylation Transformations to Afford Tn Antigen **1b** and *O*-Galactosyl Amino Acid **16b**.

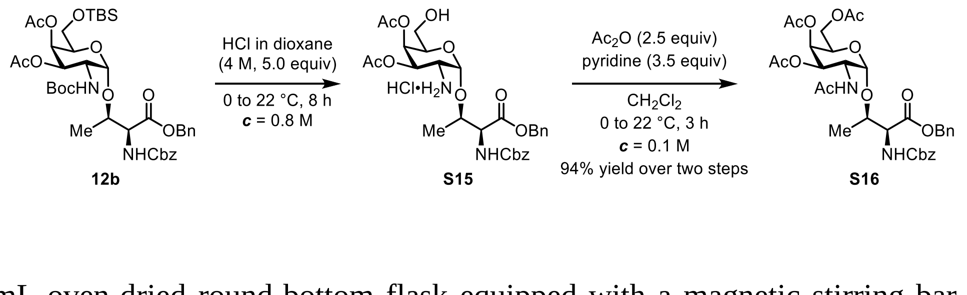

To a 100 mL oven-dried round-bottom flask equipped with a magnetic stirring bar was added compound **12b** (4 g, 4.98 mmol, 1.0 equiv). The vial was cooled to 0 °C, followed by addition

of HCl (4 M solution in 1,4-dioxane, 6.22 mL, 24.90 mmol, 5.0 equiv). The reaction mixture was stirred for 10 min at 0 °C before being warmed to room temperature. The reaction mixture was stirred for another 8 h with the progress monitored by TLC until completion. The reaction mixture was concentrated *in vacuo* with a base tube to afford the desired product **S15** as white foam which was directly used in the next step without further purification.

To the same flask containing the crude product (4.98 mmol, 1.0 equiv) from the previous step were added anhydrous CH_2Cl_2 (49.8 mL) and pyridine (1.4 mL, 17.44 mmol, 3.5 equiv). After the mixture was stirred for 3 min at 0 °C, Ac_2O (1.18 mL, 12.46 mmol, 2.5 equiv) was added dropwise. The reaction mixture was slowly warmed to room temperature and stirred for another 3 h with the progress monitored by TLC until completion. The reaction mixture was then quenched with saturated aqueous NH_4Cl solution (32 mL). The organic phase was separated from the aqueous one, which was further extracted with CH_2Cl_2 (40 mL × 3). The combined organic layer was washed with brine, dried over anhydrous Na_2SO_4, and concentrated *in vacuo.* The desired product **S16** was obtained through column chromatography (hexanes/acetone: from 20:1 to 3:2) as white foam (3.14 g, 94% yield over two steps).

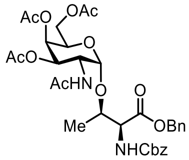

S16

***N*-Benzyloxycarbonyl-*O*-(2-acetamido-3,4,6-tri-*O*-acetyl-2-deoxy-α-D-galactopyranosyl)-L-threonine benzyl ester** (**S16**): $[\alpha]_D^{22}$ +64.5 (acetone, *c* = 1.0); IR ν_{max} (neat)/cm^{-1}: 3333 (w), 2970 (w), 2360 (m), 2342 (w), 1748 (s), 1540 (w), 1373 (w), 1218 (s), 1047 (m); ^{1}H NMR (400 MHz, $CDCl_3$) δ 7.52 – 7.28 (m, 10H), 5.72 (d, *J* = 9.6 Hz, 1H), 5.52 (d, *J* = 9.7 Hz, 1H), 5.35 (d, *J* = 3.2 Hz, 1H), 5.135 (ABq, $\Delta\nu_{AB}$ = 44.5 Hz, J_{AB} = 12.0 Hz, 2H), 5.127 (ABq, $\Delta\nu_{AB}$ = 8.0 Hz, J_{AB} = 12.2 Hz, 2H), 5.03 (dd, *J* = 11.5, 3.0 Hz, 1H), 4.78 (d, *J* = 3.8 Hz, 1H), 4.51 (ddd, *J* = 11.4, 9.6, 3.7 Hz, 1H), 4.45 (dd, *J* = 9.4, 2.3 Hz, 1H), 4.26 – 4.19 (m, 1H), 4.17 (t, *J* = 6.5 Hz, 1H), 4.12 – 3.99 (m, 2H), 2.15 (s, 3H), 2.01 (s, 3H), 1.98 (s, 3H), 1.96 (s, 3H), 1.31 (d, *J* = 6.4 Hz, 3H); ^{13}C NMR (100 MHz, $CDCl_3$) δ 170.9, 170.7, 170.30, 170.26, 170.2, 156.3, 135.8, 134.4,

128.9 (2C), 128.8 (2C), 128.6 (two peaks overlapped, 4C), 128.33, 128.25, 99.9, 76.7, 68.4, 67.8, 67.5, 67.3, 67.2, 62.1, 58.5, 47.5, 23.2, 20.7 (two peaks overlapped, 2C), 20.6, 18.2; HRMS: m/z (ESI) calcd for $C_{33}H_{41}N_2O_{13}^+$, $[M + H]^+$, 673.2603, found 673.2611. $^1J_{^{13}C1\text{–}H1}$ = 171.7 Hz.

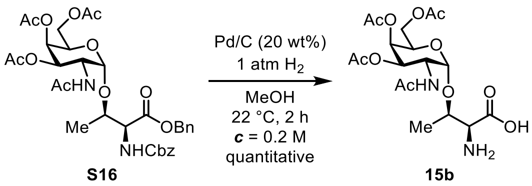

To a 100 mL round-bottom flask equipped with a magnetic stirring bar was added compound **S16** (3.14 g, 4.68 mmol, 1.0 equiv). After the flask was evacuated and backfilled with N_2 three times, Pd/C (628 mg, 20 wt %) was added. The flask was once again evacuated and backfilled with N_2 three times before MeOH (23.4 mL) was added. The flask was then evacuated and backfilled with H_2 three times and a H_2 atmosphere (1 atm) was maintained with a hydrogen balloon. The solution was stirred for 2 h at room temperature, with progress monitored by TLC until completion. The reaction mixture was then filtered through a short pad of Celite® (washed with MeOH), and the filtrate was concentrated *in vacuo* to afford the desired product **15b** (2.08 g, quantitative yield) as a white solid.

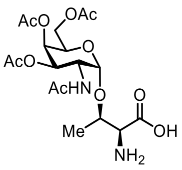

15b

***O*-(2-Acetamido-3,4,6-tri-*O*-acetyl-2-deoxy-α-D-galactopyranosyl)-L-threonine** (**15b**): $[\alpha]_D^{22}$ +67.3 (methanol, *c* = 1.0); IR ν_{max} (neat)/cm^{-1}: 2971 (w), 2360 (w), 2342 (w), 1746 (s), 1653 (m), 1540 (w), 1374 (m), 1229 (s), 1033 (m); ^{1}H NMR (400 MHz, CD_3OD) δ 5.40 (d, *J* = 3.1 Hz, 1H), 5.14 (dd, *J* = 11.5, 3.2 Hz, 1H), 5.02 (d, *J* = 3.8 Hz, 1H), 4.52 – 4.39 (m, 2H), 4.36 (t, *J* = 6.5 Hz, 1H), 4.19 – 4.04 (m, 2H), 3.60 (d, *J* = 2.1 Hz, 1H), 2.15 (s, 3H), 2.02 (s, 6H), 1.94 (s, 3H), 1.43 (d, *J* = 6.7 Hz, 3H); ^{13}C NMR (100 MHz, CD_3OD) δ 173.8, 172.12, 172.09, 172.0, 171.9, 101.4,

76.7, 70.0, 68.8, 68.5, 63.3, 60.3, 48.8, 23.1, 20.64, 20.56, 20.5, 19.0; HRMS: m/z (ESI) calcd for $C_{18}H_{29}N_2O_{11}^+$, $[M + H]^+$, 449.1766, found 449.1775. ${}^1J_{{}^{13}C1\text{–}H1}$ = 174.4 Hz.

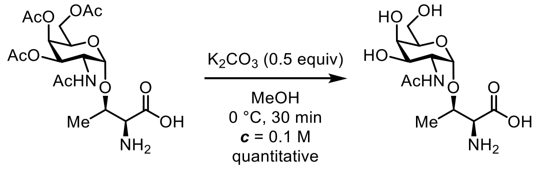

15b **1b**

To a 250 mL round-bottom flask equipped with a magnetic stirring bar were added compound **15b** (2.08 g, 4.68 mmol, 1.0 equiv) and MeOH (46.8 mL). The solution was cooled to 0 °C, and K_2CO_3 (powder, 328 mg, 2.34 mmol, 0.5 equiv) was then added. The reaction mixture was stirred for 30 min at 0 °C, with progress monitored by TLC until completion. The reaction mixture was then diluted with MeOH (40 mL) and neutralized with Amberlite® IRC 120 H. The reaction mixture was then filtered through a piece of cotton (washed with MeOH), and the filtrate was concentrated *in vacuo* to afford the desired product **1b** (1.50 g, quantitative yield) as a white solid (m.p. 208–210 °C).

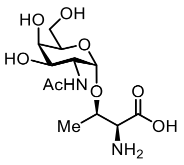

1b

***O*-(2-Acetamido-2-deoxy-α-D-galactopyranosyl)-L-threonine** (**1b**): $[\alpha]_D^{22}$ +191.6 (methanol, *c* = 0.47); IR ν_{max} (neat)/cm^{-1}: 3307 (m), 2970 (w), 2360 (s), 2342 (s), 1734 (w), 1684 (s), 1394 (w), 1229 (w), 1054 (s), 1033 (s); ^{1}H NMR (400 MHz, CD_3OD) δ 4.91 (d, *J* = 3.9 Hz, 1H), 4.37 (dt, *J* = 6.7, 2.3 Hz, 1H), 4.27 (dd, *J* = 10.9, 3.8 Hz, 1H), 3.91 (t, *J* = 6.1 Hz, 1H), 3.87 (d, *J* = 3.4 Hz, 1H), 3.79 – 3.64 (m, 3H), 3.52 (t, *J* = 2.3 Hz, 1H), 2.07 (s, 3H), 1.41 (d, *J* = 6.7 Hz, 3H); ^{13}C NMR (100 MHz, CD_3OD) δ 174.6, 172.5, 101.3, 76.1, 73.2, 70.4, 70.2, 62.8, 60.5, 51.4, 23.2, 19.2; HRMS: m/z (ESI) calcd for $C_{12}H_{23}N_2O_8^+$, $[M + H]^+$, 323.1449, found 323.1447. ${}^1J_{{}^{13}C1\text{–}H1}$ = 172.3 Hz.

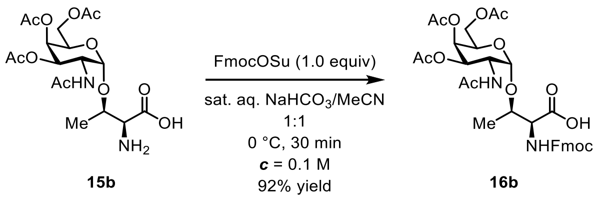

To a 250 mL round-bottom flask equipped with a magnetic stirring bar were added compound **15b** (2.08 g, 4.68 mmol, 1.0 equiv) and saturated aqueous $NaHCO_3$ (23.4 mL). The solution was cooled to 0 °C, and a precooled solution of FmocOSu (1.58 g, 4.68 mmol, 1.0 equiv) in MeCN (23.4 mL) was then added dropwise. The reaction mixture was stirred for 30 min at 0 °C, with progress monitored by TLC until completion. MeCN was then removed *in vacuo* and the remaining solution was washed with Et_2O (40 mL × 2). The aqueous phase was acidified with aqueous HCl solution (1 M) at 0 °C until pH reaches 2. EtOAc (60 mL × 3) was then used to extract the aqueous phase. The combined organic layer was washed with brine, dried over anhydrous Na_2SO_4, and concentrated *in vacuo* to afford the desired product **16b** (2.88 g, 92% yield) as a white solid (m.p. 148–150 °C).

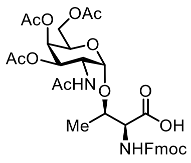

16b

***N*-Fluorenylmethyloxycarbonyl-*O*-(2-acetamido-3,4,6-tri-*O*-acetyl-2-deoxy-α-D-galactopyranosyl)-L-threonine** (**16b**): $[\alpha]^{22}_{D}$ +71.9 (acetone, *c* = 0.13); IR ν_{max} (neat)/cm^{-1}: 2970 (w), 2570 (w), 2360 (s), 2342 (m), 2159 (w), 1792 (s), 1653 (m), 1374 (w), 1229 (s), 1054 (m); ^{1}H NMR (400 MHz, CD_3OD, 328 K) δ 7.80 (d, *J* = 7.6 Hz, 2H), 7.67 (t, *J* = 7.6 Hz, 2H), 7.38 (t, *J* = 7.6 Hz, 2H), 7.31 (t, *J* = 7.6 Hz, 2H), 5.40 (d, *J* = 3.2 Hz, 1H), 5.08 (dd, *J* = 11.5, 3.2 Hz, 1H), 4.65 – 4.52 (m, 2H), 4.51 – 4.44 (m, 1H), 4.41 (dd, *J* = 11.4, 3.8 Hz, 1H), 4.38 – 4.33 (m, 1H), 4.32 – 4.19 (m, 3H), 4.18 – 4.00 (m, 2H), 2.13 (s, 3H), 2.02 (s, 3H), 1.94 (s, 6H), 1.25 (d, *J* = 5.0 Hz, 3H); ^{13}C NMR (100 MHz, CD_3OD) δ 173.68, 173.67, 172.13, 172.11, 172.0, 159.1, 145.4, 145.2, 142.74, 142.71, 128.8, 128.2, 126.2 (2C), 126.1 (2C), 121.00, 120.96, 100.8, 77.8, 69.8,

68.8, 68.2, 67.7, 63.3, 60.0, 48.7, 48.6, 22.9, 20.64, 20.58, 20.5, 19.2; HRMS: m/z (ESI) calcd for $C_{33}H_{39}N_2O_{13}^+$, $[M + H]^+$, 671.2447, found 671.2429. $^1J_{^{13}C1\text{–}H1}$ = 174.9 Hz.

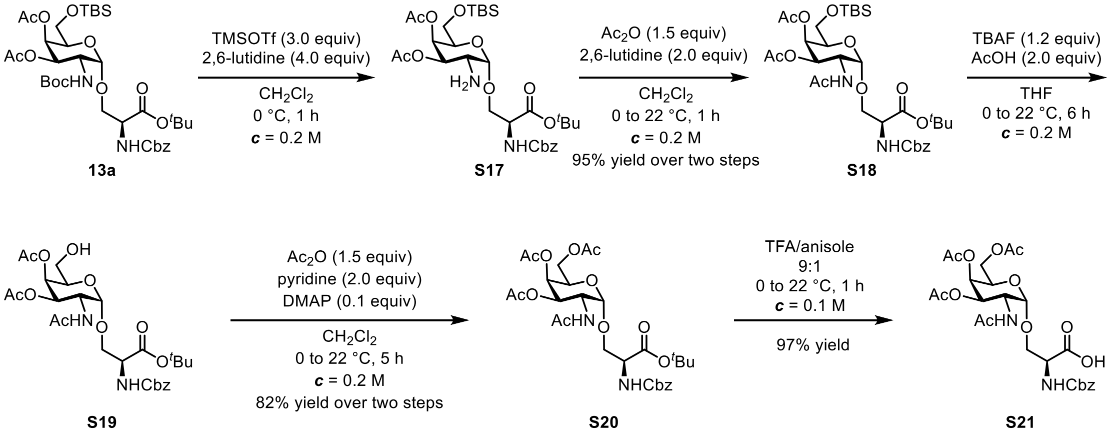

Figure S10. Post-glycosylation Transformations to Afford *O*-Galactosyl Amino Acid **S21**.

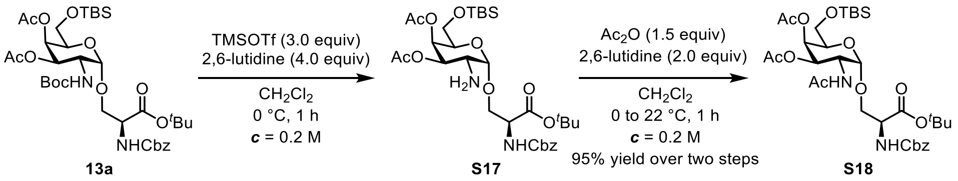

To a flame-dried sealable 2-dram vial equipped with a stir bar was added compound **13a** (200 mg, 0.265 mmol, 1.0 equiv) and anhydrous CH_2Cl_2 (1.3 mL). The vial was cooled to 0 °C, followed by sequential addition of 2,6-lutidine (123 μL, 1.06 mmol, 4.0 equiv) and TMSOTf (144 μL, 0.795 mmol, 3.0 equiv). The reaction mixture was stirred for 1 h at 0 °C with the progress monitored by TLC until completion. The reaction was diluted with CH_2Cl_2 (2 mL) and quenched with saturated aqueous NH_4Cl (2 mL). The mixture was then stirred for 20 min at room temperature before the organic phase was separated from the aqueous one, which was further extracted with CH_2Cl_2 (3 mL × 3). The combined organic layer was washed with brine, dried over anhydrous Na_2SO_4, and concentrated *in vacuo* to afford the desired product **S17** as white foam which was directly used in the next step without further purification.

To a sealable 2-dram vial equipped with a stir bar containing the crude product (0.265 mmol, 1.0 equiv) from the previous step were added anhydrous CH_2Cl_2 (1.33 mL) and 2,6-lutidine (61 μL, 0.53 mmol, 2.0 equiv). After the mixture was stirred for 3 min at 0 °C, Ac_2O (38 μL, 0.398 mmol, 1.5 equiv) was added dropwise. The reaction mixture was warmed to room temperature and stirred for 1 h with the progress monitored by TLC until completion. The reaction mixture was then quenched with saturated aqueous NH_4Cl solution (1 mL). The organic phase was separated from the aqueous one, which was further extracted with CH_2Cl_2 (2 mL × 3). The combined organic layer was washed with brine, dried over anhydrous Na_2SO_4, and concentrated *in vacuo*. The desired product **S18** was obtained through column chromatography (hexanes/ EtOAc: from 20:1 to 1:2) as white foam (175 mg, 95% yield over two steps).

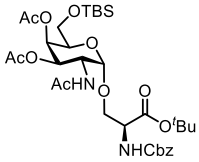

S18

***N*-Benzyloxycarbonyl-*O*-(2-acetamido-3,4-di-*O*-acetyl-6-*O*-*tert*-butyldimethylsilyl-2-deoxy-α-D-galactopyranosyl)-L-serine *tert*-butyl ester** (**S18**): $[\alpha]_D^{22}$ +56.1 (acetone, *c* = 1.0); IR ν_{max} (neat)/cm^{-1}: 3332 (w), 2931 (w), 2360 (w), 1747 (s), 1522 (w), 1369 (m), 1241 (s), 1219 (s), 1155 (m), 1049 (m); ^1H NMR (400 MHz, $CDCl_3$) δ 7.42 – 7.28 (m, 5H), 5.69 (d, *J* = 5.8 Hz, 1H), 5.61 (d, *J* = 7.3 Hz, 1H), 5.41 (d, *J* = 3.2 Hz, 1H), 5.21 – 5.09 (m, 2H), 5.08 (d, *J* = 3.2 Hz, 1H), 4.80 (d, *J* = 3.6 Hz, 1H), 4.56 (ddd, *J* = 11.5, 9.7, 3.6 Hz, 1H), 4.41 (d, *J* = 8.2 Hz, 1H), 4.02 – 3.89 (m, 2H), 3.79 (dd, *J* = 10.7, 3.7 Hz, 1H), 3.62 (dd, *J* = 9.9, 6.2 Hz, 1H), 3.55 (dd, *J* = 9.9, 7.3 Hz, 1H), 2.12 (s, 3H), 1.97 (s, 3H), 1.92 (s, 3H), 1.45 (s, 9H), 0.85 (s, 9H), 0.01 (s, 3H), 0.00 (s, 3H); ^{13}C NMR (100 MHz, $CDCl_3$) δ 170.9, 170.0 (two peaks overlapped, 2C), 169.1, 155.8, 136.0, 128.5 (2C), 128.24 (2C), 128.19, 98.9, 82.9, 69.8, 69.1, 68.8, 67.2, 67.1, 60.9, 54.7, 47.7, 28.0 (3C), 25.7 (3C), 23.2, 20.7 (two peaks overlapped, 2C), 18.1, -5.6, -5.7; HRMS: m/z (ESI) calcd for $C_{33}H_{53}N_2O_{12}Si^+$, $[M + H]^+$, 697.3362, found 697.3380. $^1J_{^{13}C1-H1}$ = 172.4 Hz.

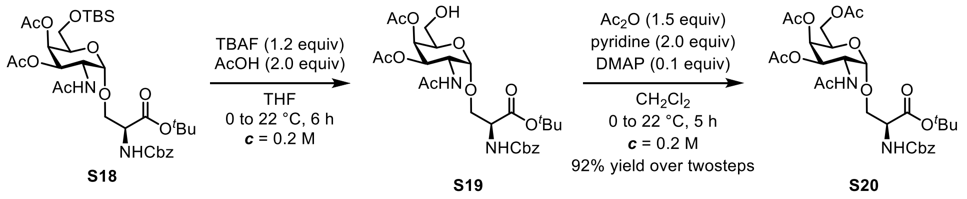

To a sealable 2-dram vial equipped with a stir bar was added compound **S18** (150 mg, 0.215 mmol, 1.0 equiv) and THF (0.82 mL). The vial was cooled to 0 °C, followed by addition of a premixed solution of TBAF (1 M in THF, 0.26 mL, 0.258 mmol, 1.2 equiv) and AcOH (25 μL, 0.43 mmol, 2.0 equiv). The reaction mixture was then warmed to room temperature and stirred for 6 h with the progress monitored by TLC until completion. The reaction mixture was concentrated *in vacuo* and the residue was further azeotropically dried with anhydrous toluene (3 mL × 2). The crude product **S19** was obtained as white foam and directly used in the next step without further purification.

To sealable 2-dram vial equipped with a stir bar containing the crude product (0.215 mmol, 1.0 equiv) from the previous step were added anhydrous CH_2Cl_2 (1.08 mL), DMAP (2.6 mg, 0.022 mmol, 0.1 equiv), and pyridine (35 μL, 0.43 mmol, 2.0 equiv). After the mixture was stirred for 3 min at 0 °C, Ac_2O (31 μL, 0.323 mmol, 1.5 equiv) was added dropwise. The reaction mixture was warmed to room temperature and stirred for 5 h with the progress monitored by TLC until completion. The reaction mixture was then quenched with saturated aqueous NH_4Cl solution (1 mL). The organic phase was separated from the aqueous one, which was further extracted with CH_2Cl_2 (1.5 mL × 3). The combined organic layer was washed with brine, dried over anhydrous Na_2SO_4, and concentrated *in vacuo.* The desired product **S20** was obtained through column chromatography (hexanes/ acetone: from 20:1 to 2:1) as white foam (124 mg, 92% yield over two steps).

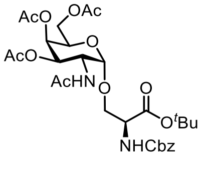

S20

***N*-Benzyloxycarbonyl-*O*-(2-acetamido-3,4,6-tri-*O*-acetyl-2-deoxy-α-D-galactopyranosyl)-L-serine *tert*-butyl ester** (**S20**): $[\alpha]_D^{22}$ +78.6 (acetone, *c* = 1.0); IR ν_{max} (neat)/cm^{-1}: 3356 (w), 2971 (w), 2360 (w), 2342 (w), 1746 (s), 1558 (w), 1370 (m), 1228 (s), 1052 (m); ^{1}H NMR (400 MHz, $CDCl_3$) δ 7.47 – 7.27 (m, 5H), 5.75 – 5.52 (m, 2H), 5.35 (d, *J* = 3.2 Hz, 1H), 5.12 (s, 2H), 5.08 (dd, *J* = 11.3, 3.2 Hz, 1H), 4.84 (d, *J* = 3.7 Hz, 1H), 4.58 (ddd, *J* = 11.5, 9.7, 3.7 Hz, 1H), 4.42 (d, *J* = 7.8 Hz, 1H), 4.16 – 4.05 (m, 2H), 4.03 (td, *J* = 9.1, 4.6 Hz, 1H), 3.96 (dd, *J* = 10.0, 1.6 Hz, 1H), 3.82 (dd, *J* = 10.6, 3.4 Hz, 1H), 2.14 (s, 3H), 2.02 (s, 3H), 1.98 (s, 3H), 1.92 (s, 3H), 1.46 (s, 9H); ^{13}C NMR (100 MHz, $CDCl_3$) δ 170.8, 170.4, 170.3, 170.0, 168.9, 155.7, 136.0, 128.6 (2C), 128.3 (2C), 128.2, 98.7, 83.0, 69.2, 68.4, 67.24, 67.22, 67.18, 61.9, 54.7, 47.5, 28.0 (3C), 23.2, 20.71, 20.70, 20.6; HRMS: m/z (ESI) calcd for $C_{29}H_{41}N_2O_{13}^+$, $[M + H]^+$, 625.2603, found 625.2614. $^{1}J_{^{13}C1–H1}$ = 174.6 Hz.

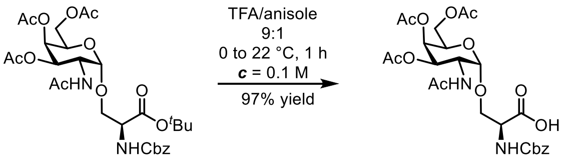

S20 **S21**

To sealable 2-dram vial equipped with a stir bar was added compound **S20** (50 mg, 0.08 mmol, 1 equiv). After the vial was cooled to 0 °C, a precooled mixture of TFA (0.72 mL) and anisole (0.08 mL) was added. The reaction mixture was slowly warmed to room temperature and stirred for 1 h with the progress monitored by TLC until completion. The reaction mixture was then concentrated *in vacuo* and the remaining TFA and anisole were removed azeotropically with toluene (3 mL × 2). The desired product **S21** was obtained as a white solid (44 mg, 97% yield).

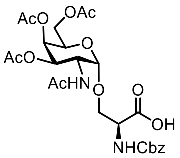

S21

***N*-Benzyloxycarbonyl-*O*-(2-acetamido-3,4,6-tri-*O*-acetyl-2-deoxy-α-D-galactopyranosyl)-L-serine ester** (**S21**): $[\alpha]_D^{22}$ +89.9 (acetone, *c* = 1.0); IR ν_{max} (neat)/cm^{-1}: 3351 (w), 2937 (w), 2360 (w), 1745 (s), 1533 (w), 1372 (w), 1220 (s), 1049 (m); ^1H NMR (400 MHz, acetone-d_6) δ 7.60 – 7.05 (m, 5H), 5.36 (d, *J* = 3.4 Hz, 1H), 5.16 – 5.02 (m, 3H), 4.97 (d, *J* = 3.6 Hz, 1H), 4.53 (t, *J* = 3.6 Hz, 1H), 4.42 (dd, *J* = 11.6, 3.6 Hz, 1H), 4.35 (t, *J* = 6.5 Hz, 1H), 4.14 (dd, *J* = 11.2, 6.0 Hz, 1H), 4.09 – 3.99 (m, 3H), 2.11 (s, 3H), 1.97 (s, 3H), 1.88 (s, 3H), 1.85 (s, 3H); ^{13}C NMR (100 MHz, acetone-d_6) δ 171.8, 170.8 (two peaks overlapped, 2C), 170.6 (two peaks overlapped, 2C), 157.0, 138.0, 129.3 (2C), 128.9 (2C), 128.8, 99.7, 69.5, 68.9, 68.2, 67.9, 67.0, 62.6, 55.1, 48.3, 22.8, 20.7, 20.6 (two peaks overlapped, 2C); HRMS: m/z (ESI) calcd for $C_{25}H_{33}N_2O_{13}^+$, [M + H]$^+$, 569.1977, found 569.1958. $^1J_{^{13}C1-H1}$ = 172.2 Hz.

E. References

1. Kozikowski, A. P.; Lee, J. A Synthetic Approach to the *cis*-Fused Marine Pyranopyrans, (3*E*)- and (3*Z*)-Dactomelyne. X-Ray Structure of a Rare Organomercurial. *J. Org. Chem.* **1990**, *55*, 863.

2. Li, H.; Zhang, D.; Li, C.; Yin, L.; Jiang, Z.; Luo, Y.; Xu, H. Stereoselective Glycosylation for 1,2-*cis*-Aminoglycoside Assembly by Cooperative Atom Transfer Catalysis. *J. Am. Chem. Soc.* **2024**, *146*, 33316.

3. Marshall, J. A.; Beaudoin, S. Stereoselective Synthesis of Differentially Protected Derivatives of the Higher Amino Sugars Destomic Acid and Lincosamine from Serine and Threonine. *J. Org. Chem.* **1996**, *61*, 581.

4. Kirschning, A. Oxidation of Fully Protected Glycals by Hypervalent Iodine Reagents. *J. Org. Chem.* **1995**, *60*, 1228.

5. Khaled, A.; Gravier-Pelletier, C.; Le Merrer, Y. Synthesis of Bis-(2,3,4,6-Tetra-*O*-Acetyl-α-D-Mannopyranosyl)-L-Serinyl Phosphate, as a Prodrug of Mannose-1-Phosphate. *Tetrahedron: Asymmetry* **2007**, *18*, 2121.

6. Milewska, K. D.; Malins, L. R. Synthesis of Amino Acid α-Thioethers and Late-Stage Incorporation into Peptides. *Org. Lett.* **2022**, *24*, 3680.

7. Sowinski, J. A.; Toogood, P. L. Synthetic Studies Towards Keramamide F. *Tetrahedron Lett.* **1995**, *36*, 67.

8. Huang, Y.; Dey, S.; Zhang, X.; Sönnichsen, F.; Garner, P. The α-Helical Peptide Nucleic Acid Concept: Merger of Peptide Secondary Structure and Codified Nucleic Acid Recognition. *J. Am. Chem. Soc.* **2004**, *126*, 4626.

9. Nicolaou, K. C.; Mitchell, H. J.; Jain, N. F.; Winssinger, N.; Hughes, R.; Bando, T. Total Synthesis of Vancomycin. *Angew. Chem. Int. Ed.* **1999**, *38*, 240.

F. NMR Spectra

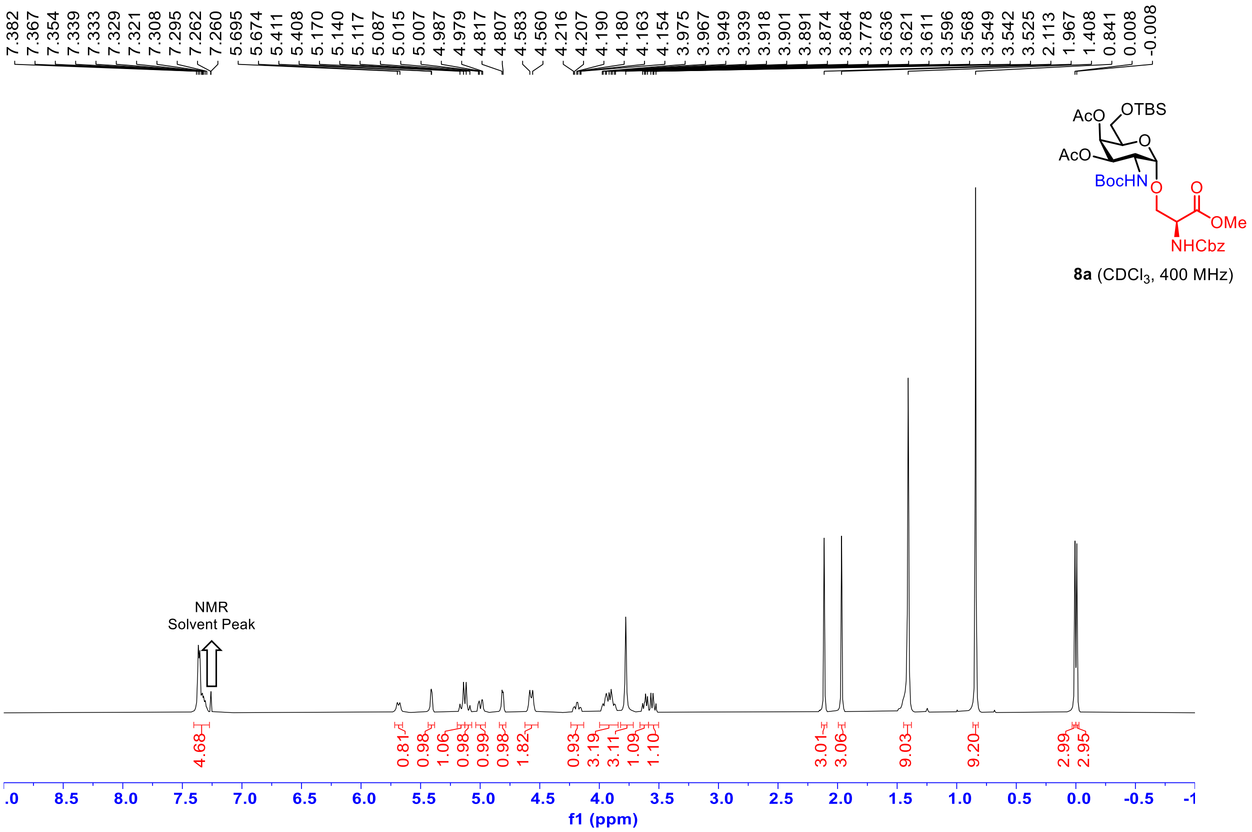

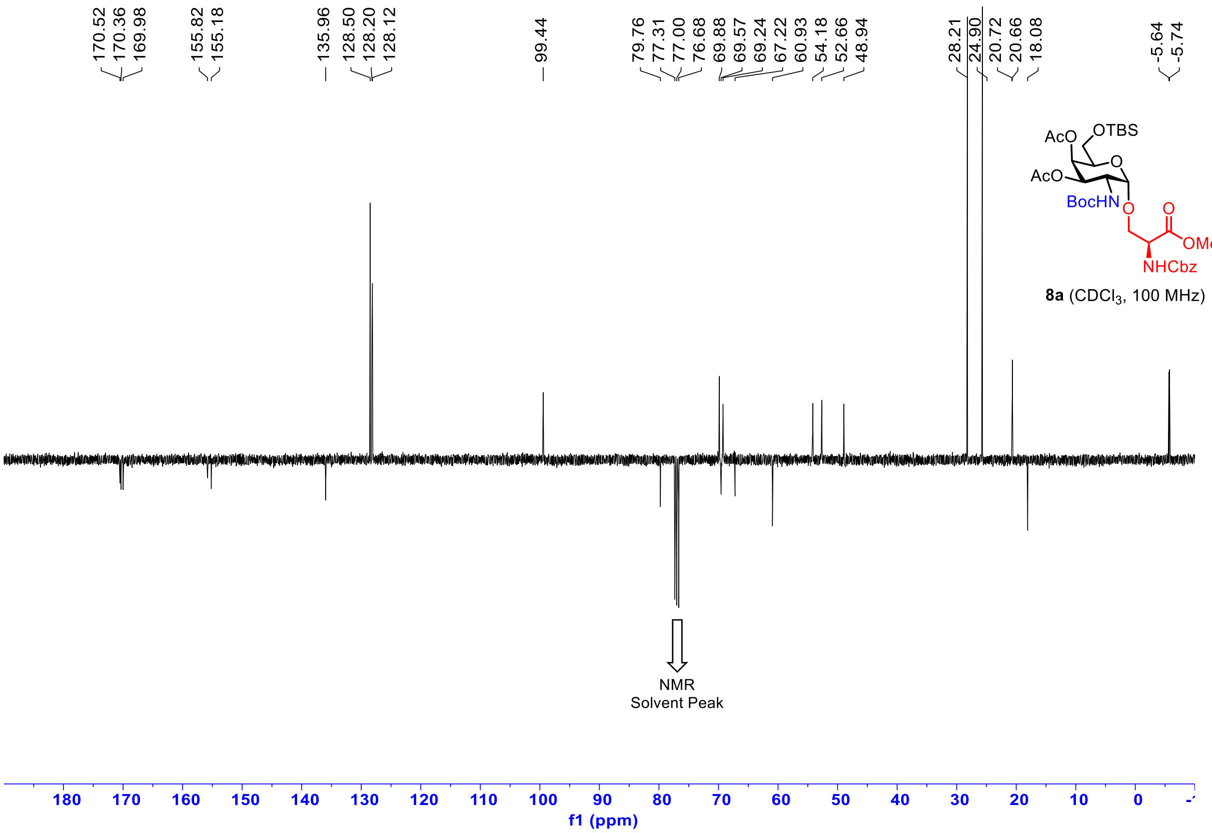

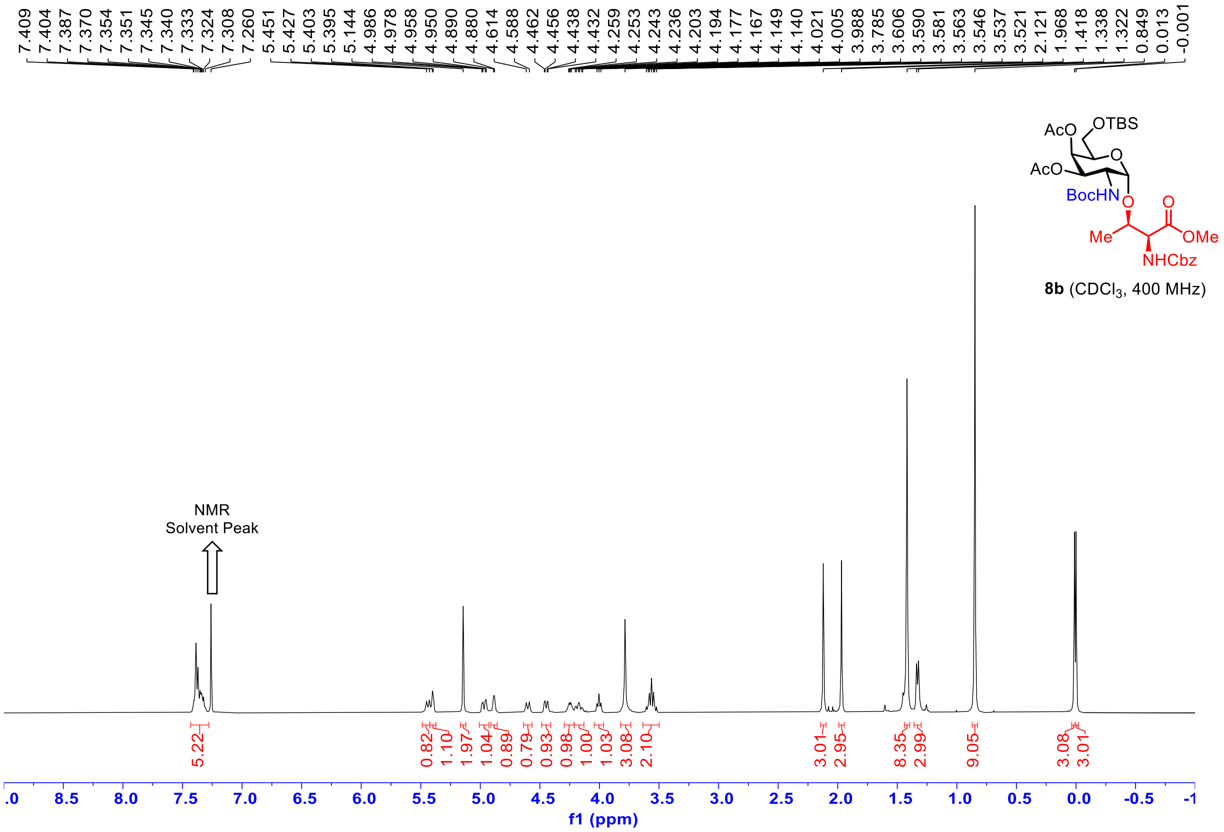
7.409 7.404 7.387 7.370 7.354 7.351 7.345 7.340 7.333 7.324 7.308 7.260 5.451 5.427 5.403 5.395 5.144 4.986 4.978 4.958 4.950 4.890 4.880 4.614 4.588 4.462 4.456 4.438 4.432 4.259 4.253 4.243 4.236 4.203 4.194 4.177 4.167 4.149 4.140 4.021 4.005 3.988 3.785 3.606 3.590 3.581 3.563 3.546 3.537 3.521 2.121 1.968 1.418 1.338 1.322 0.849 0.013 -0.001
AcO
OTBS
O
AcO
BocHN
O
O
Me
OMe
NHCbz
8b (CDCl3, 400 MHz)
NMR
Solvent Peak
5.22
0.82 1.10 1.97 1.04 0.89 0.79 0.93 0.98 1.00 1.03 3.08 2.10
3.01 2.95
8.35 2.99
9.05
3.08 3.01
f1 (ppm)

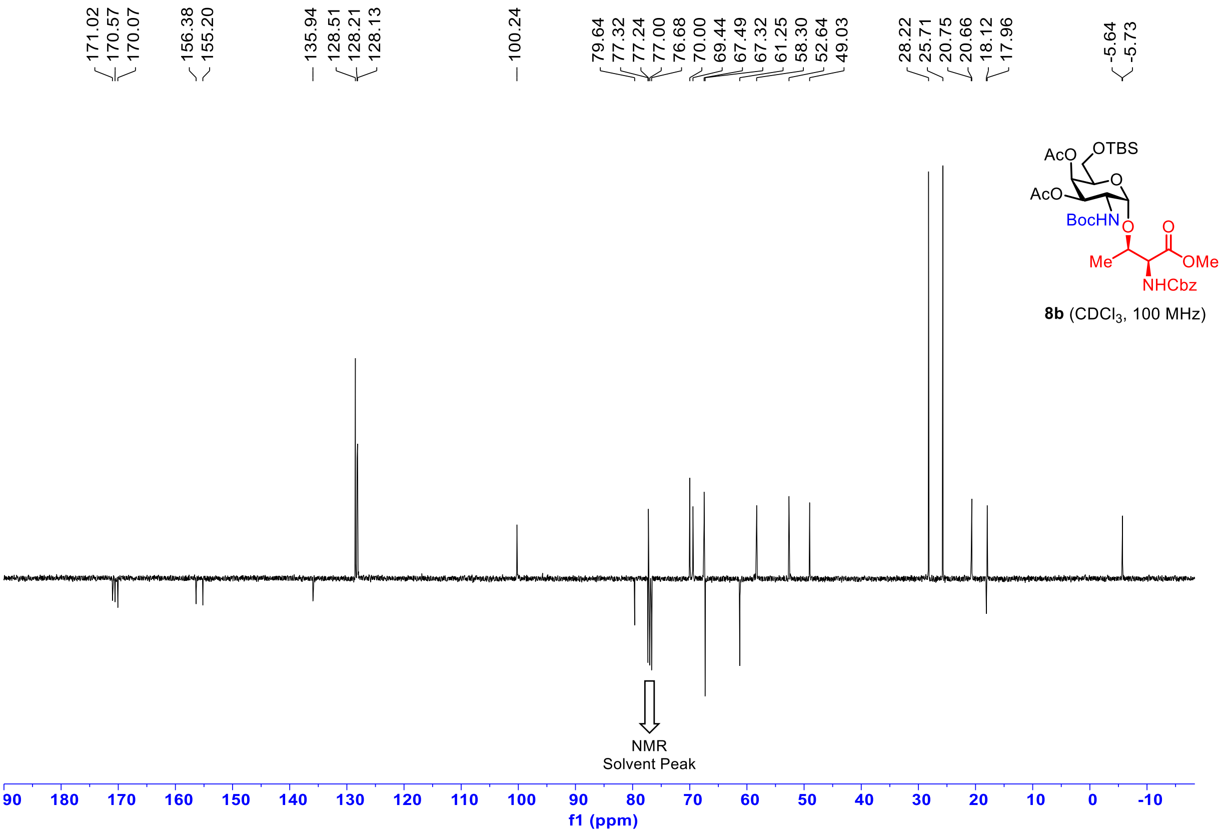
171.02 170.57 170.07
156.38 155.20
135.94 128.51 128.21 128.13
100.24
79.64 77.32 77.24 77.00 76.68 70.00 69.44 67.49 67.32 61.25 58.30 52.64 49.03
28.22 25.71 20.75 20.66 18.12 17.96
-5.64 -5.73
AcO
OTBS
O
AcO
BocHN
O
O
Me
OMe
NHCbz
8b (CDCl3, 100 MHz)
NMR
Solvent Peak
f1 (ppm)

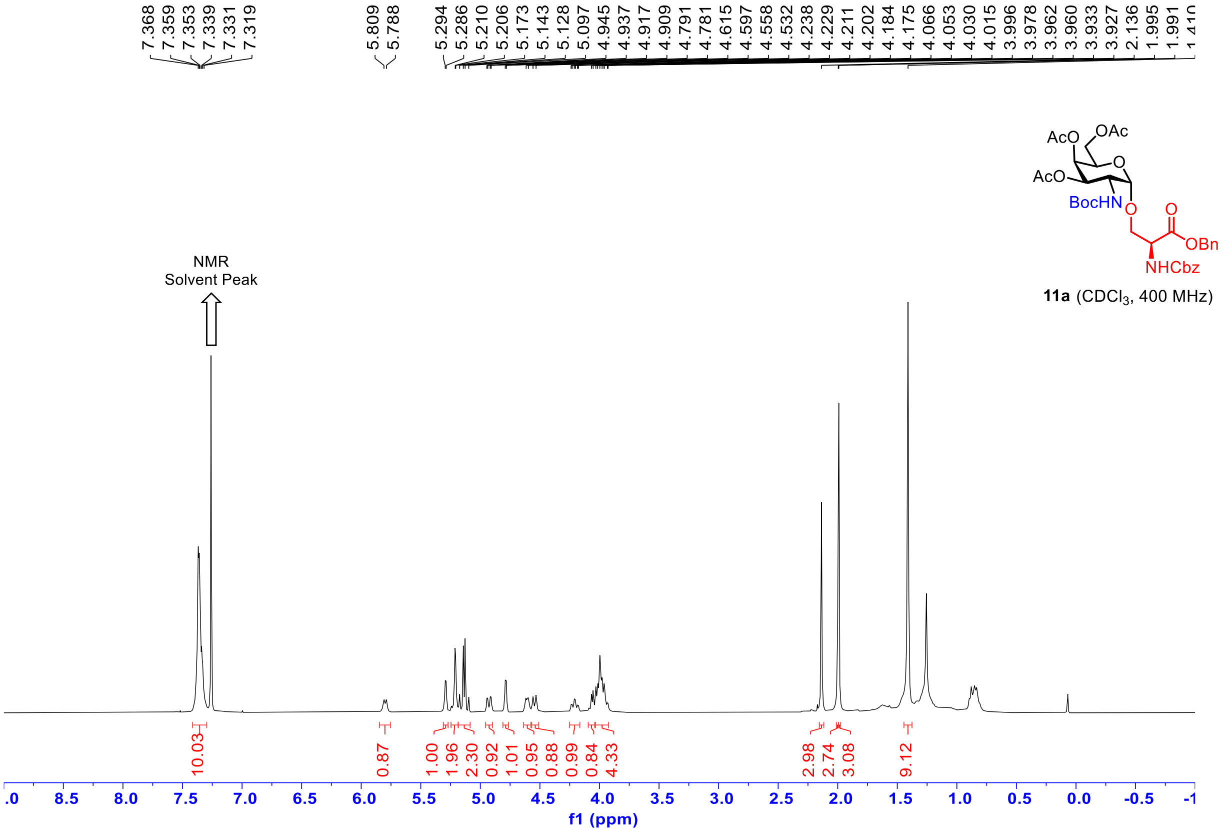
7.368
7.359
7.353
7.339
7.331
7.319
5.809
5.788
5.294
5.286
5.210
5.206
5.173
5.143
5.128
5.097
4.945
4.937
4.917
4.909
4.791
4.781
4.615
4.597
4.558
4.532
4.238
4.229
4.211
4.202
4.184
4.175
4.066
4.053
4.030
4.015
3.996
3.978
3.962
3.960
3.933
3.927
2.136
1.995
1.991
AcO
OAc
O
AcO
BocHN
O
O
OBn
NHCbz
11a (CDCl3, 400 MHz)
NMR
Solvent Peak
10.03
0.87
1.00
1.96
2.30
0.92
1.01
0.95
0.88
0.99
0.84
4.33
2.98
2.74
3.08
9.12
f1 (ppm)

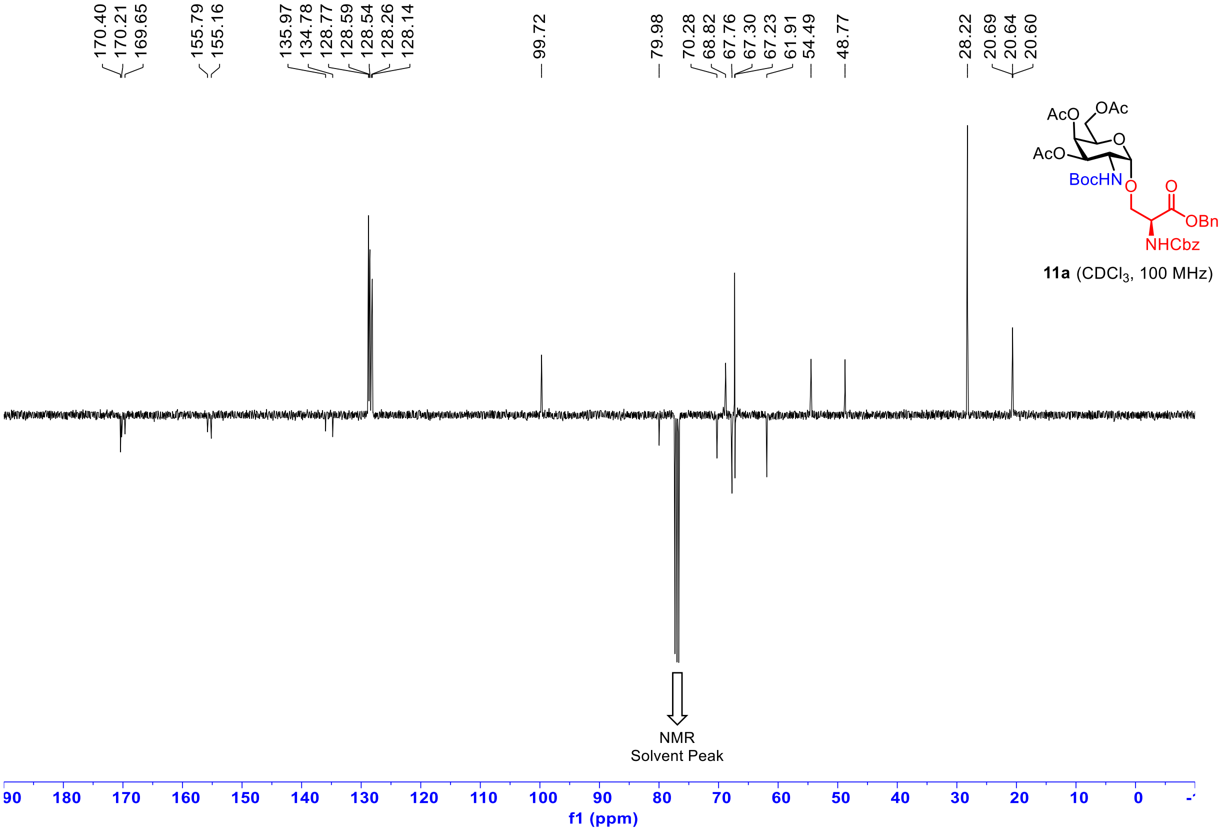
170.40
170.21
169.65
155.79
155.16
135.97
134.78
128.77
128.59
128.54
128.26
128.14
99.72
79.98
70.28
68.82
67.76
67.30
67.23
61.91
54.49
48.77
28.22
20.69
20.64
20.60
AcO
OAc
O
AcO
BocHN
O
O
OBn
NHCbz
11a (CDCl3, 100 MHz)
NMR
Solvent Peak
f1 (ppm)

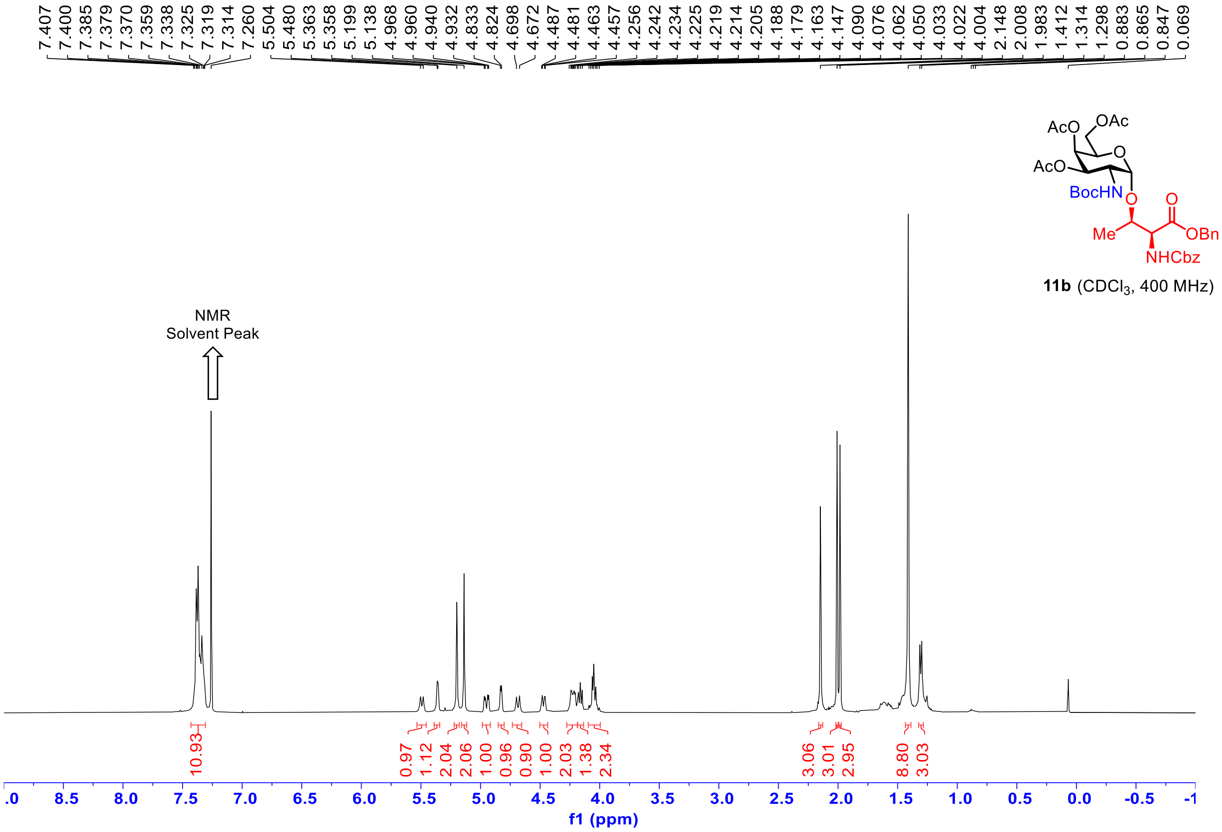
7.407
7.400
7.385
7.379
7.370
7.359
7.338
7.325
7.319
7.314
7.260
5.504
5.480
5.363
5.358
5.199
5.138
4.968
4.960
4.940
4.932
4.833
4.824
4.698
4.672
4.487
4.481
4.463
4.457
4.256
4.242
4.234
4.225
4.219
4.214
4.205
4.188
4.179
4.163
4.147
4.090
4.076
4.062
4.050
4.033
4.022
4.004
2.148
2.008
1.983
1.412
1.314
1.298
0.883
0.865
0.847
0.069
AcO
OAc
AcO
O
BocHN
O
Me
O
OBn
NHCbz
11b (CDCl3, 400 MHz)
NMR
Solvent Peak
10.93
0.97
1.12
2.04
2.06
1.00
0.96
0.90
1.00
2.03
1.38
2.34
3.06
3.01
2.95
8.80
3.03
f1 (ppm)

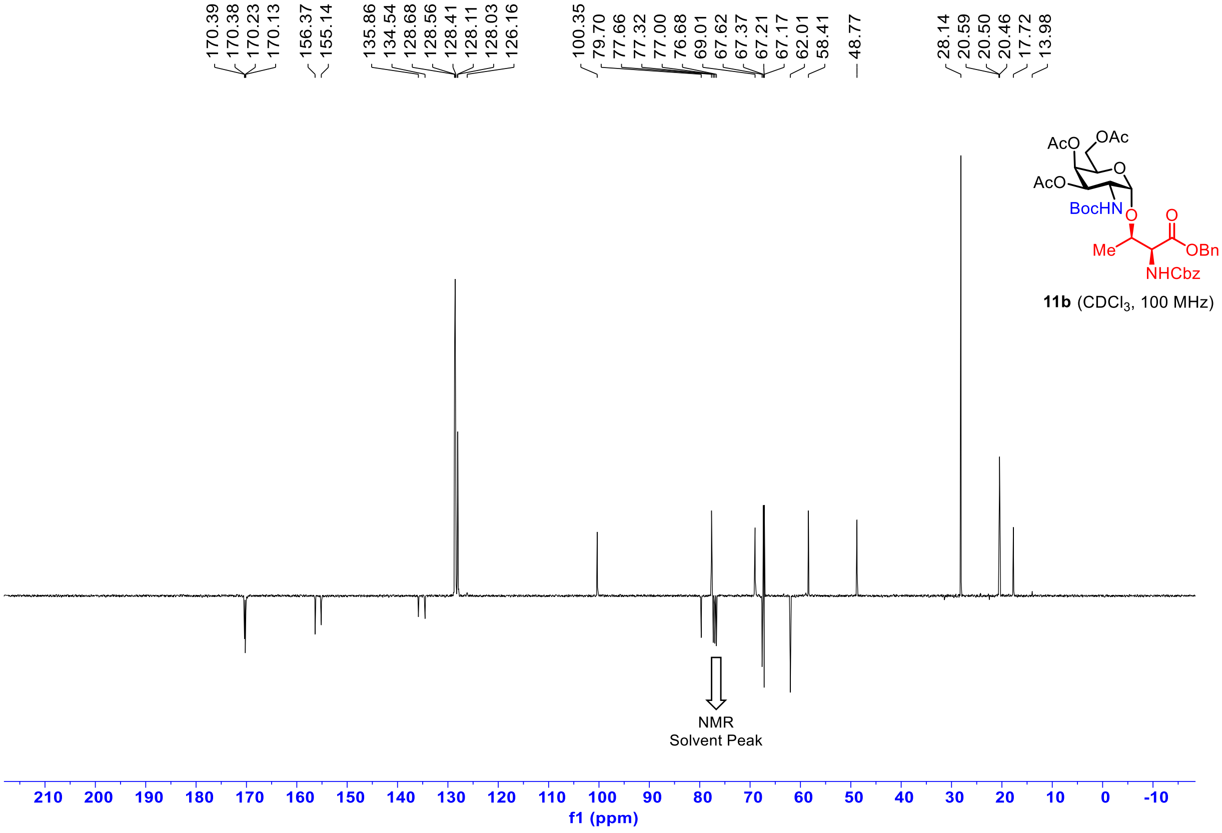
170.39
170.38
170.23
170.13
156.37
155.14
135.86
134.54
128.68
128.56
128.41
128.11
128.03
126.16
100.35
79.70
77.66
77.32
77.00
76.68
69.01
67.62
67.37
67.21
67.17
62.01
58.41
48.77
28.14
20.59
20.50
20.46
17.72
13.98
AcO
OAc
AcO
O
BocHN
O
Me
O
OBn
NHCbz
11b (CDCl3, 100 MHz)
NMR
Solvent Peak
f1 (ppm)

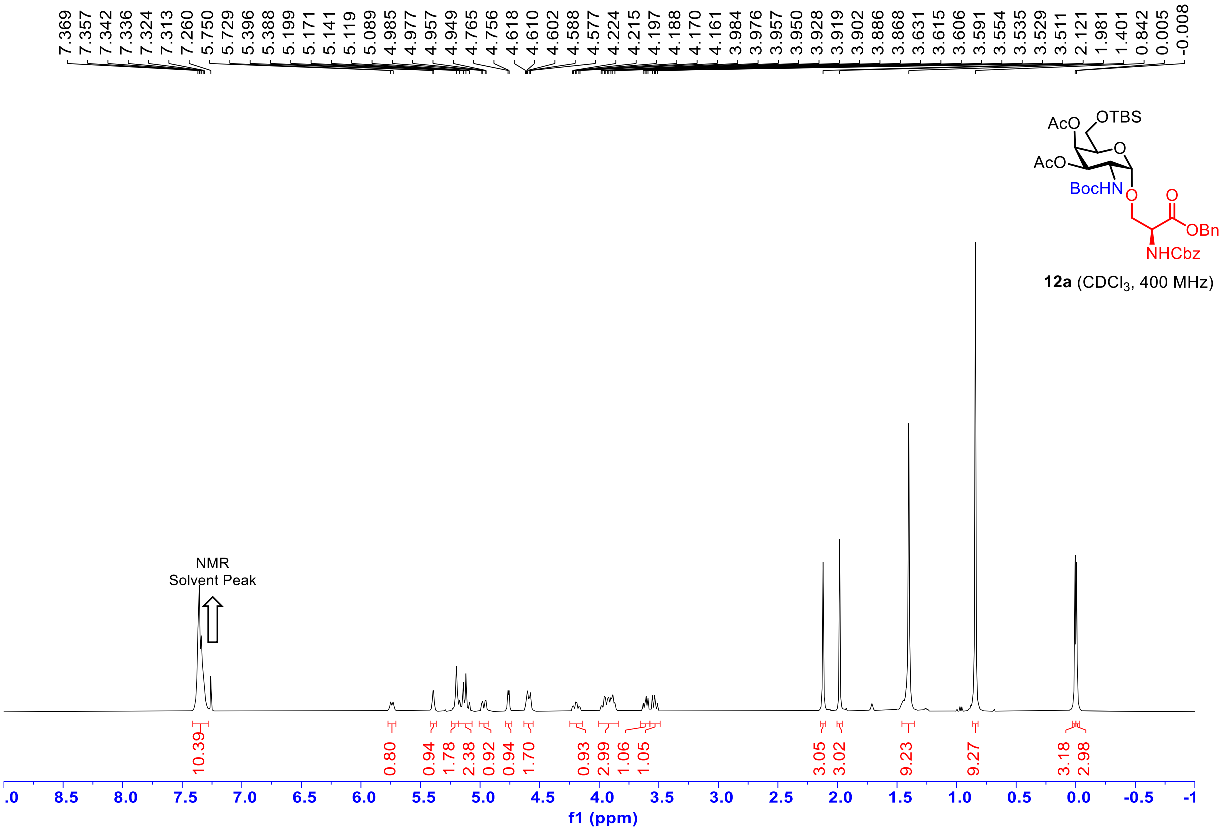
7.369 7.357 7.342 7.336 7.324 7.313 7.260 5.750 5.729 5.396 5.388 5.199 5.171 5.141 5.119 5.089 4.985 4.977 4.957 4.949 4.765 4.756 4.618 4.610 4.602 4.588 4.577 4.224 4.215 4.197 4.188 4.170 4.161 3.984 3.976 3.957 3.950 3.928 3.919 3.902 3.886 3.868 3.631 3.615 3.606 3.591 3.554 3.535 3.529 3.511 2.121 1.981 1.401 0.842 0.005 -0.008
AcO
OTBS
O
AcO
BocHN
O
O
OBn
NHCbz
12a (CDCl3, 400 MHz)
NMR
Solvent Peak
10.39
0.80
0.94
1.78
2.38
0.92
0.94
1.70
0.93
2.99
1.06
1.05
3.05
3.02
9.23
9.27
3.18
2.98
f1 (ppm)

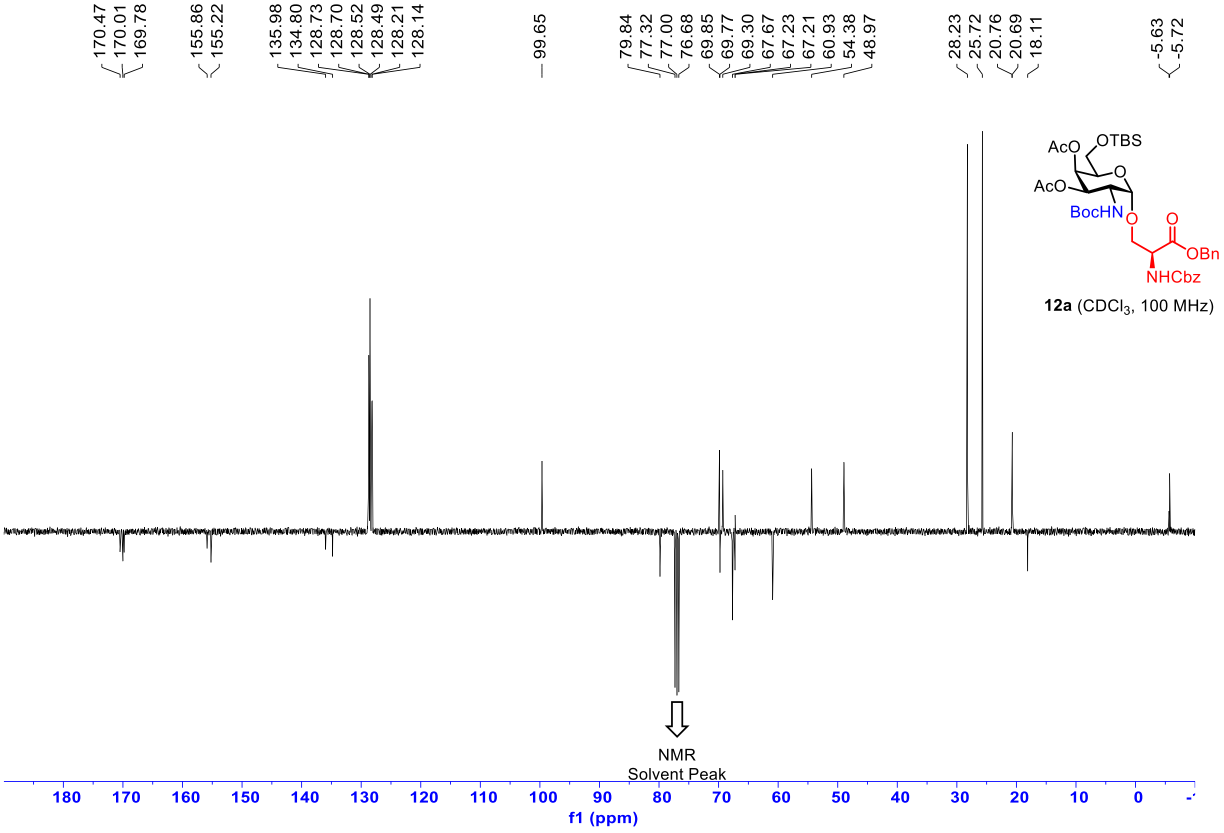
170.47 170.01 169.78 155.86 155.22 135.98 134.80 128.73 128.70 128.52 128.49 128.21 128.14 99.65 79.84 77.32 77.00 76.68 69.85 69.77 69.30 67.67 67.23 67.21 60.93 54.38 48.97 28.23 25.72 20.76 20.69 18.11 -5.63 -5.72
AcO
OTBS
O
AcO
BocHN
O
O
OBn
NHCbz
12a (CDCl3, 100 MHz)
NMR
Solvent Peak
180 170 160 150 140 130 120 110 100 90 80 70 60 50 40 30 20 10 0
f1 (ppm)

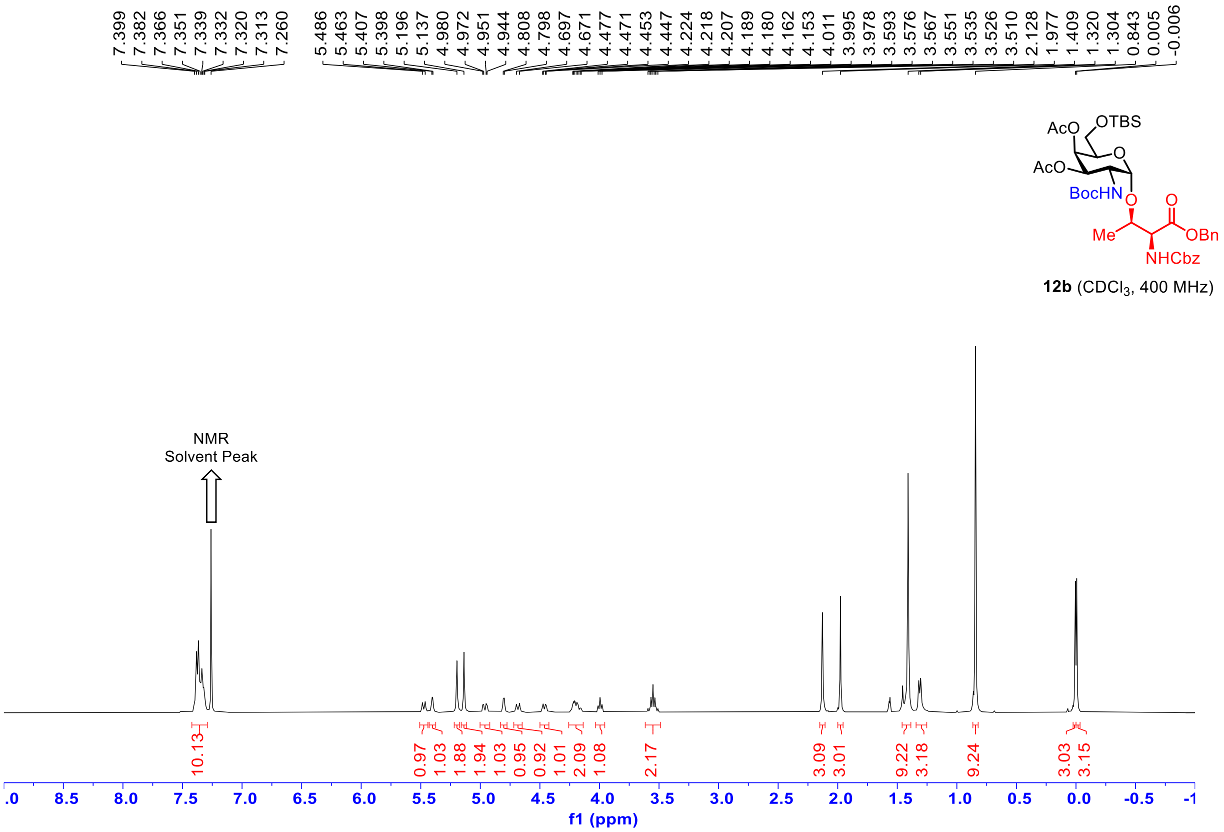

12b (CDCl3, 400 MHz)
NMR Solvent Peak
f1 (ppm)

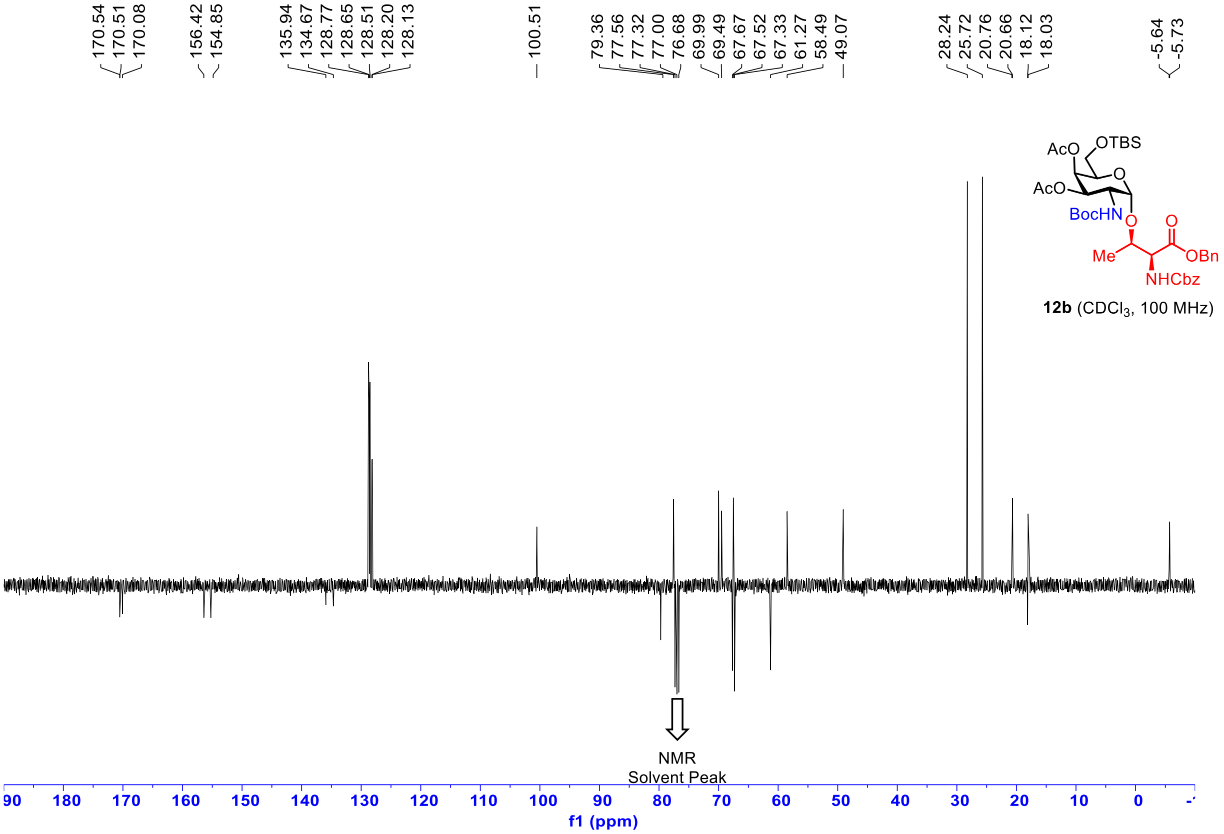

12b (CDCl3, 100 MHz)
NMR Solvent Peak
f1 (ppm)

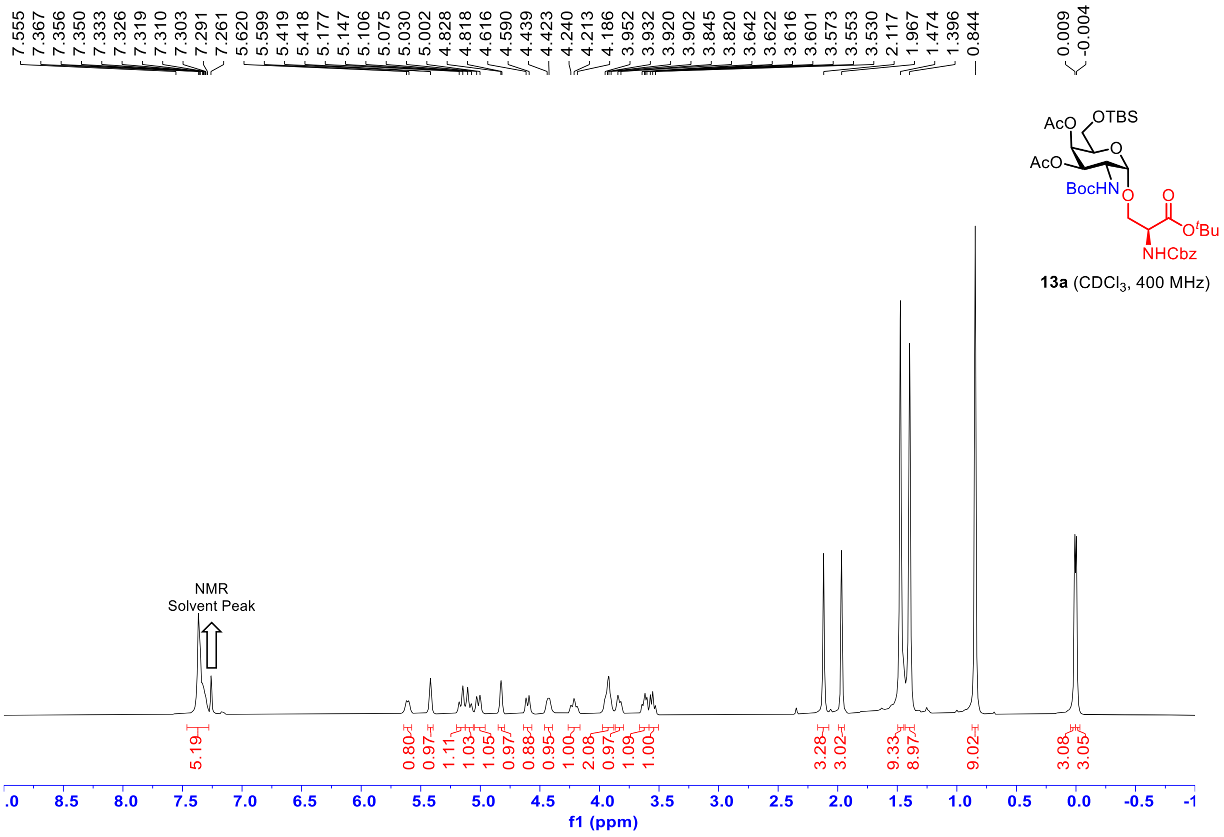
7.555 7.367 7.356 7.350 7.333 7.326 7.319 7.310 7.303 7.291 7.261 5.620 5.599 5.419 5.418 5.177 5.147 5.106 5.075 5.030 5.002 4.828 4.818 4.616 4.590 4.439 4.423 4.240 4.213 4.186 3.952 3.932 3.920 3.902 3.845 3.820 3.642 3.622 3.616 3.601 3.573 3.553 3.530 2.117 1.967 1.474 1.396 0.844 0.009 -0.004
AcO
OTBS
AcO
O
BocHN
O
O
O^{t}Bu
NHCbz
13a (CDCl$_3$, 400 MHz)
NMR
Solvent Peak
5.19 0.80 0.97 1.11 1.03 1.05 0.97 0.88 0.95 1.00 2.08 0.97 1.09 1.00 3.28 3.02 9.33 8.97 9.02 3.08 3.05
f1 (ppm)

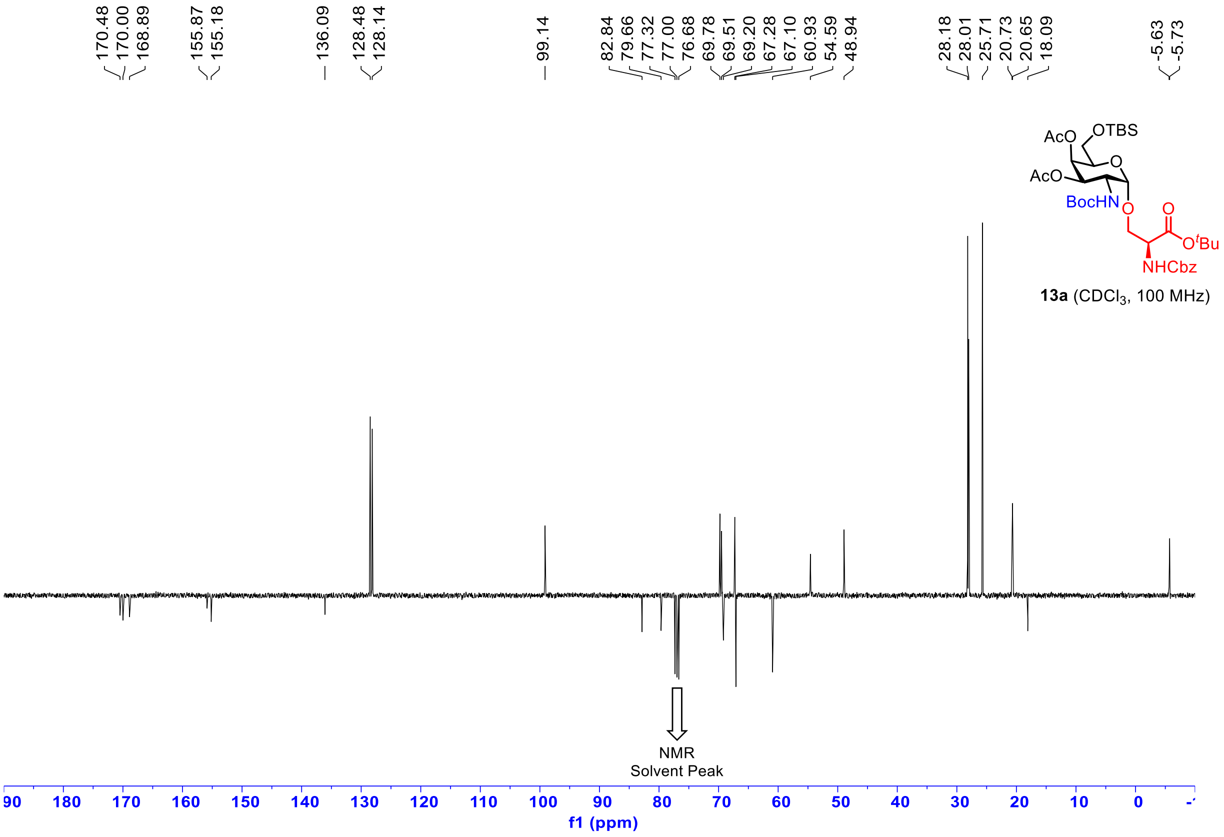
170.48 170.00 168.89 155.87 155.18 136.09 128.48 128.14 99.14 82.84 79.66 77.32 77.00 76.68 69.78 69.51 69.20 67.28 67.10 60.93 54.59 48.94 28.18 28.01 25.71 20.73 20.65 18.09 -5.63 -5.73
AcO
OTBS
AcO
O
BocHN
O
O
O^{t}Bu
NHCbz
13a (CDCl$_3$, 100 MHz)
NMR
Solvent Peak
f1 (ppm)

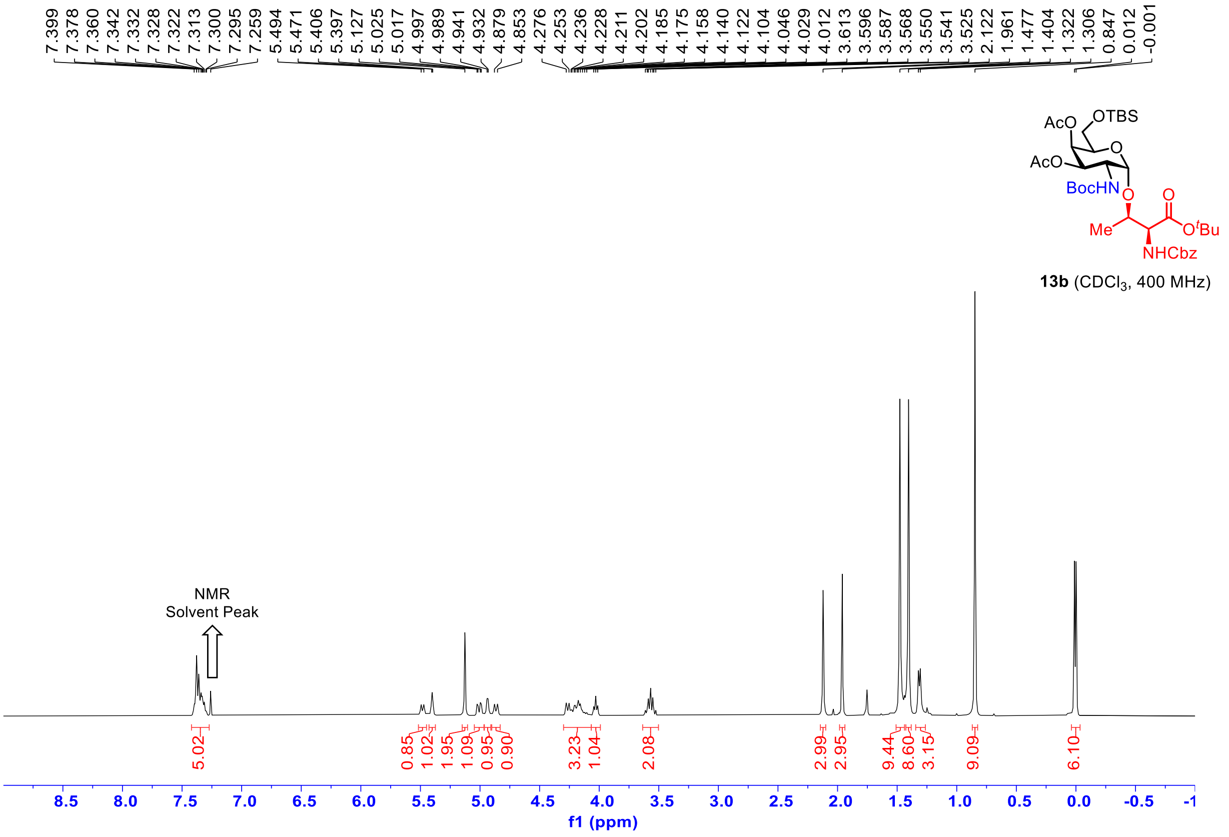

13b ($CDCl_3$, 400 MHz)

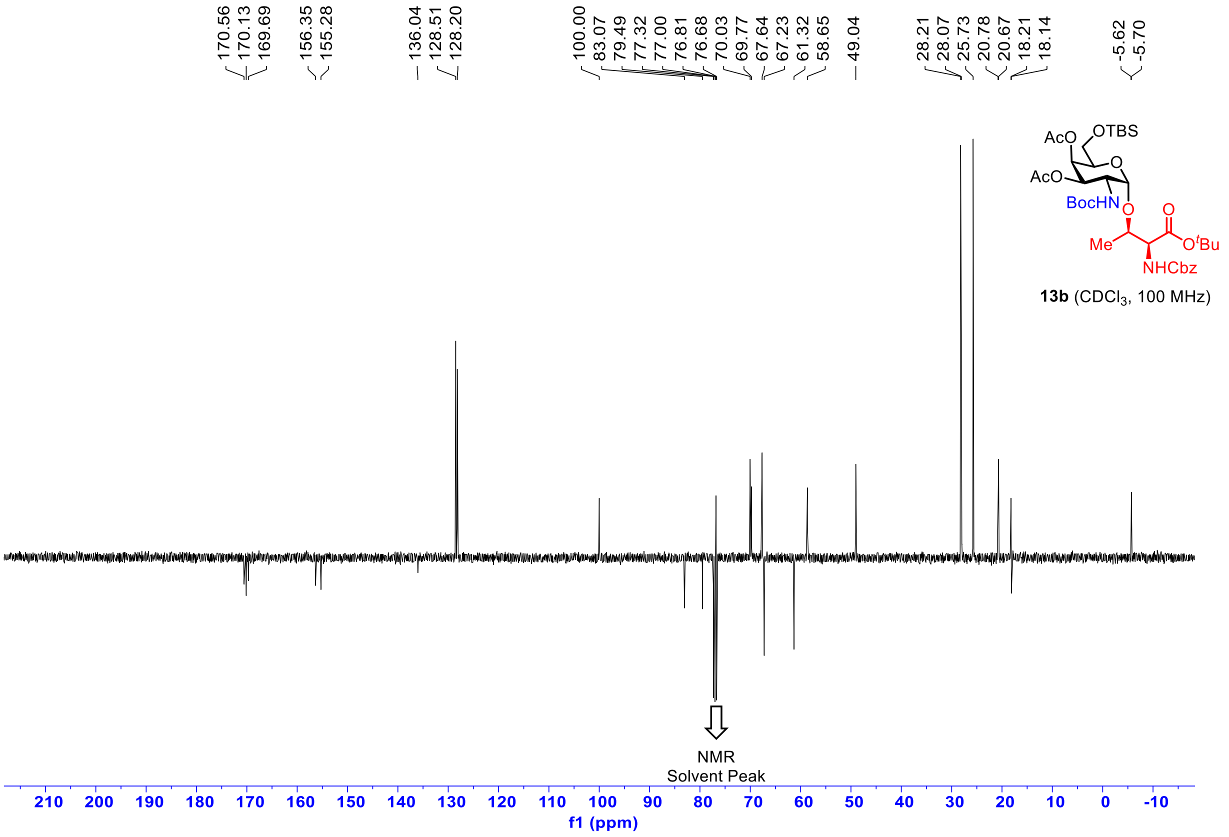

13b ($CDCl_3$, 100 MHz)

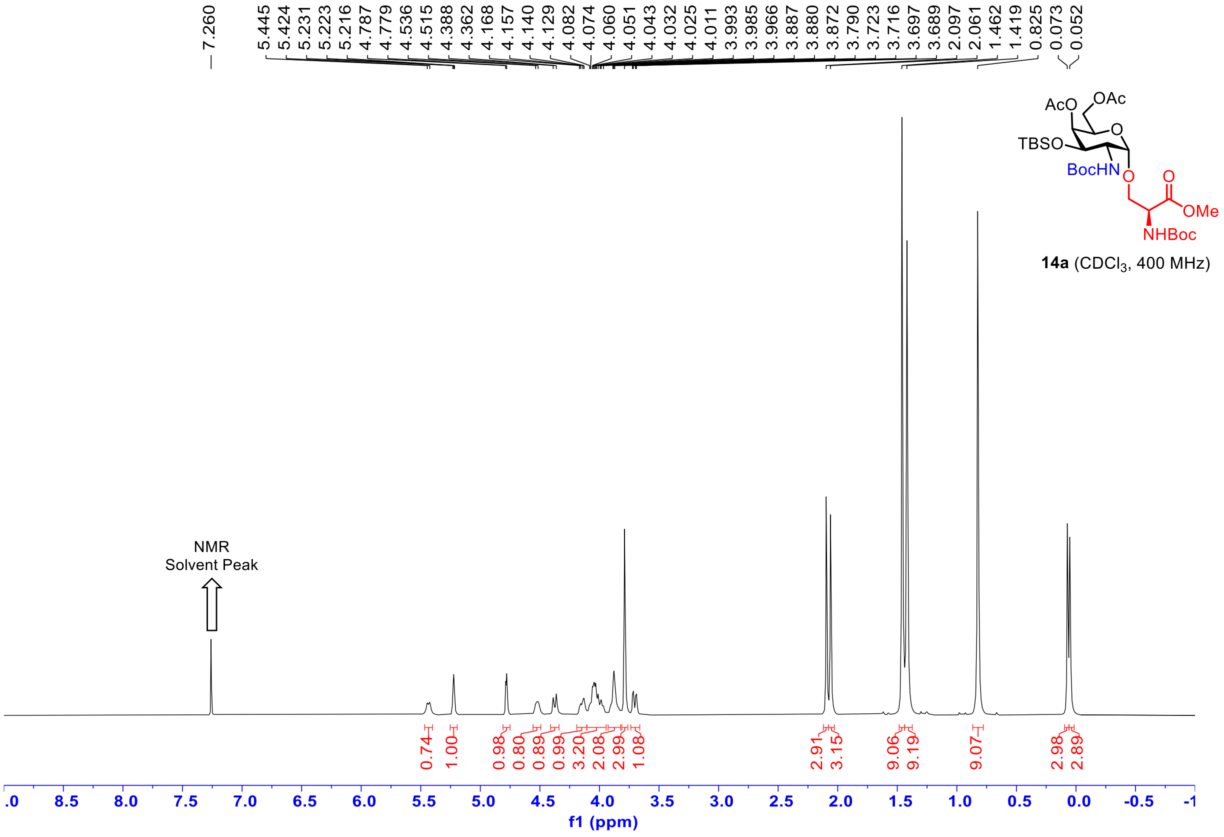
7.260
5.445
5.424
5.231
5.223
5.216
4.787
4.779
4.536
4.515
4.388
4.362
4.168
4.157
4.140
4.129
4.082
4.074
4.060
4.051
4.043
4.032
4.025
4.011
3.993
3.985
3.966
3.887
3.880
3.872
3.790
3.723
3.716
3.697
3.689
2.097
2.061
1.462
1.419
0.825
0.073
0.052
AcO
OAc
TBSO
BocHN
O
OMe
NHBoc
14a (CDCl3, 400 MHz)
NMR
Solvent Peak
0.74
1.00
0.98
0.80
0.89
0.99
3.20
2.08
2.99
1.08
2.91
3.15
9.06
9.19
9.07
2.98
2.89
f1 (ppm)

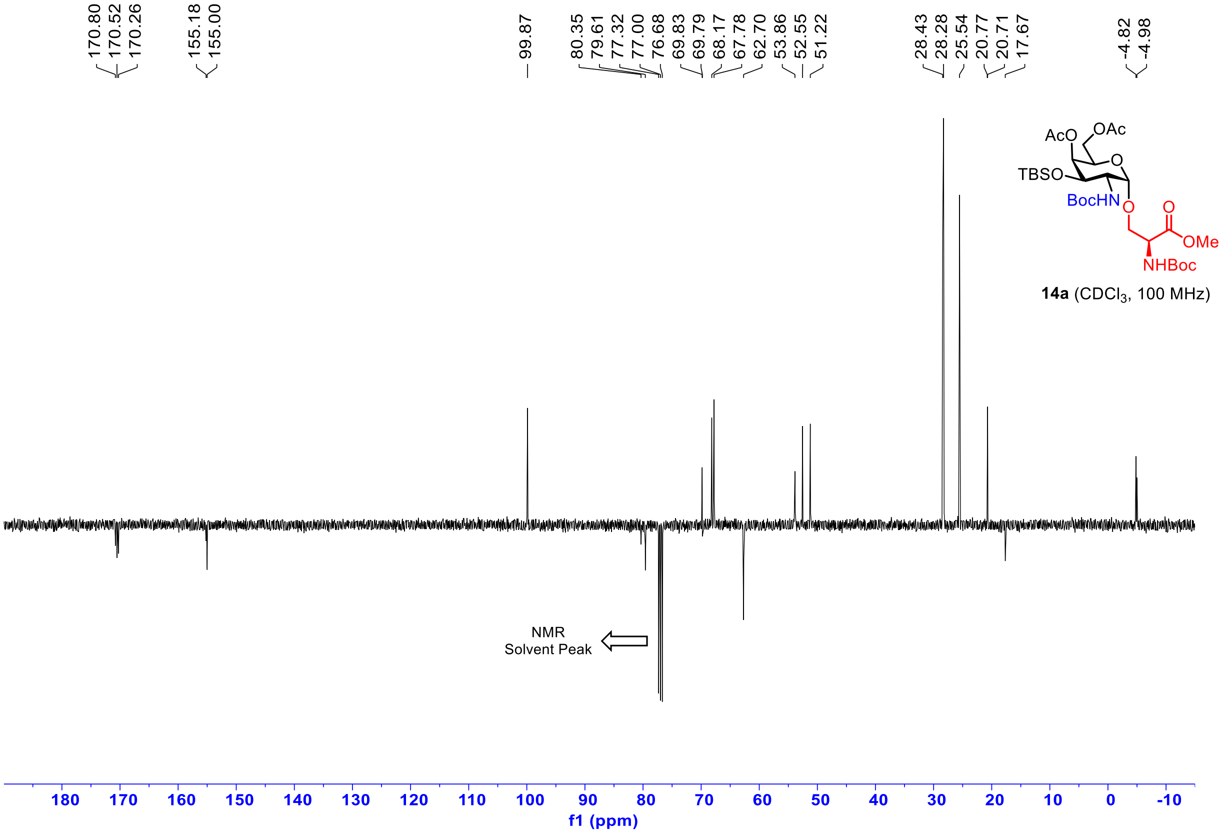
170.80
170.52
170.26
155.18
155.00
99.87
80.35
79.61
77.32
77.00
76.68
69.83
69.79
68.17
67.78
62.70
53.86
52.55
51.22
28.43
28.28
25.54
20.77
20.71
17.67
-4.82
-4.98
AcO
OAc
TBSO
BocHN
O
OMe
NHBoc
14a (CDCl3, 100 MHz)
NMR
Solvent Peak
f1 (ppm)

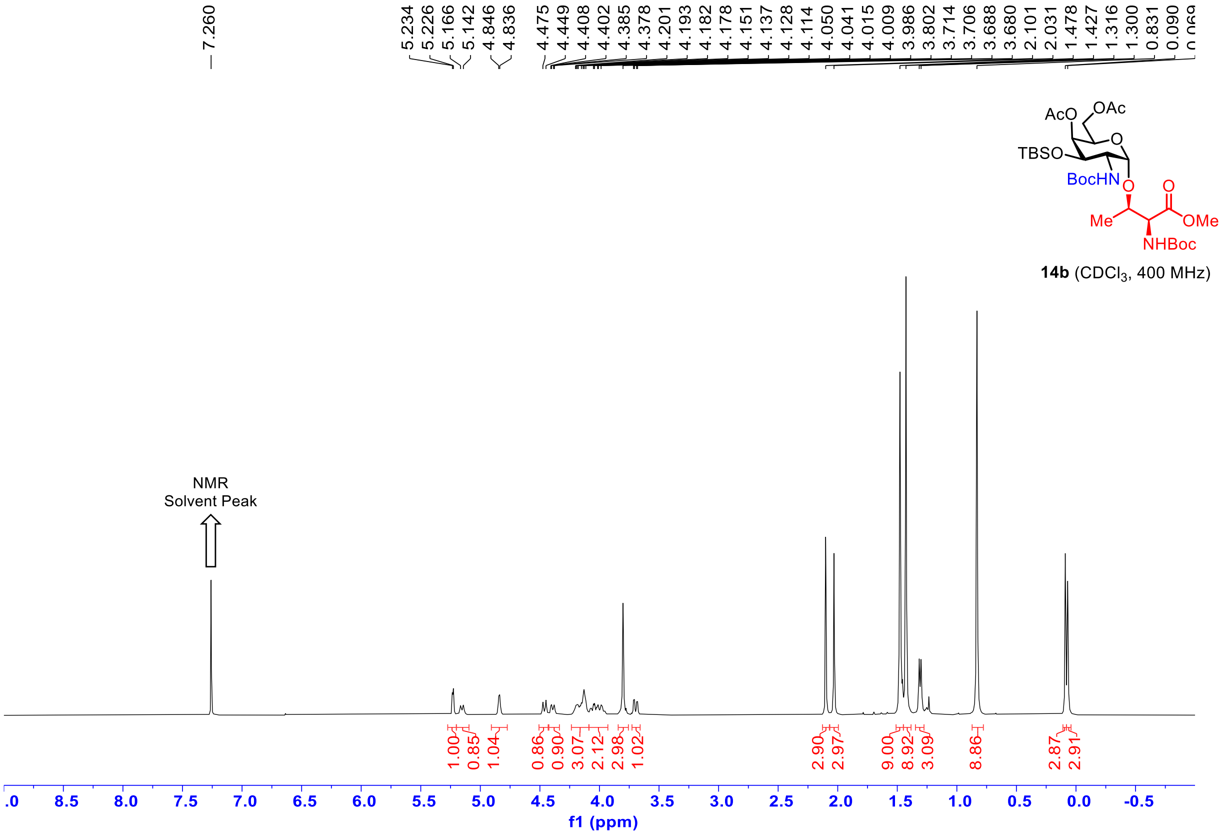
7.260
5.234
5.226
5.166
5.142
4.846
4.836
4.475
4.449
4.408
4.402
4.385
4.378
4.201
4.193
4.182
4.178
4.151
4.137
4.128
4.114
4.050
4.041
4.015
4.009
3.986
3.802
3.714
3.706
3.688
3.680
2.101
2.031
1.478
1.427
1.316
1.300
0.831
0.090
AcO
OAc
TBSO
BocHN
O
Me
OMe
NHBoc
14b (CDCl3, 400 MHz)
NMR
Solvent Peak
1.00
0.85
1.04
0.86
0.90
3.07
2.12
2.98
1.02
2.90
2.97
9.00
8.92
3.09
8.86
2.87
2.91
f1 (ppm)

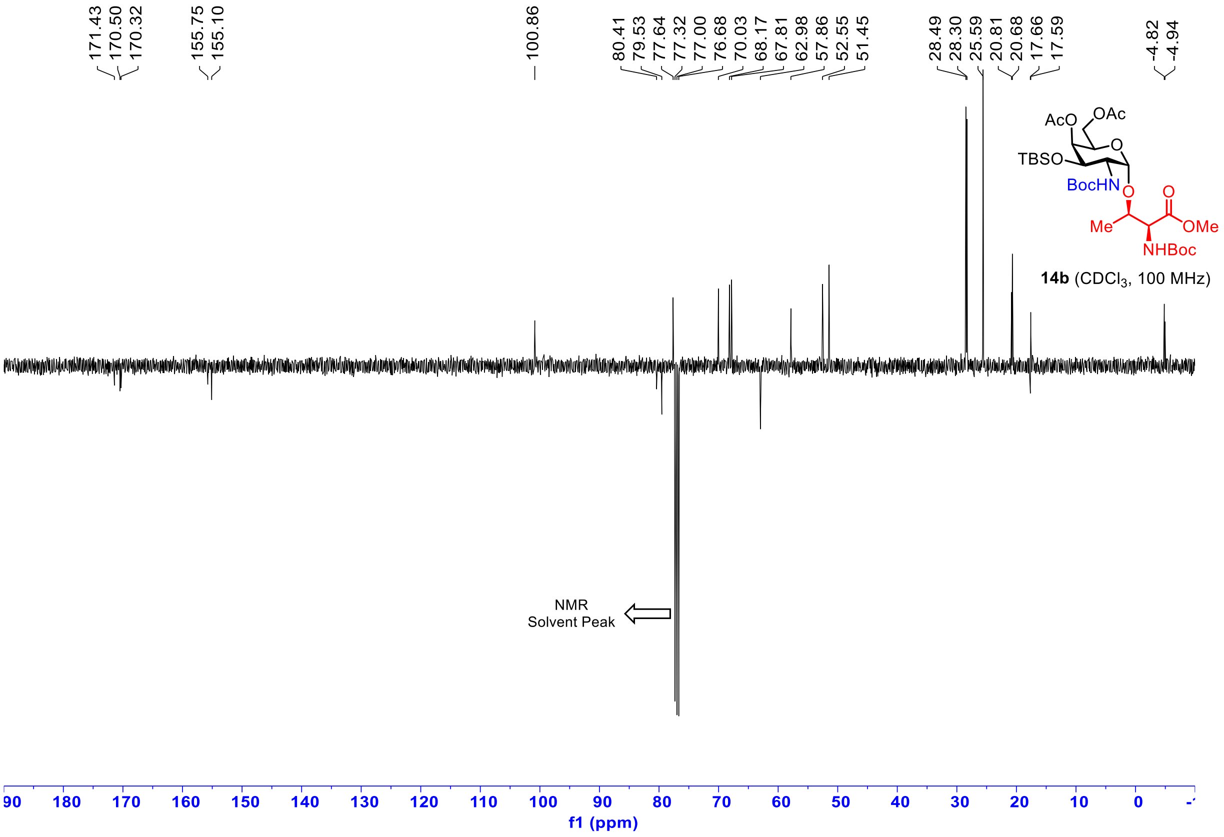
171.43
170.50
170.32
155.75
155.10
100.86
80.41
79.53
77.64
77.32
77.00
76.68
70.03
68.17
67.81
62.98
57.86
52.55
51.45
28.49
28.30
25.59
20.81
20.68
17.66
17.59
-4.82
-4.94
AcO
OAc
TBSO
BocHN
O
Me
OMe
NHBoc
14b (CDCl3, 100 MHz)
NMR
Solvent Peak
f1 (ppm)

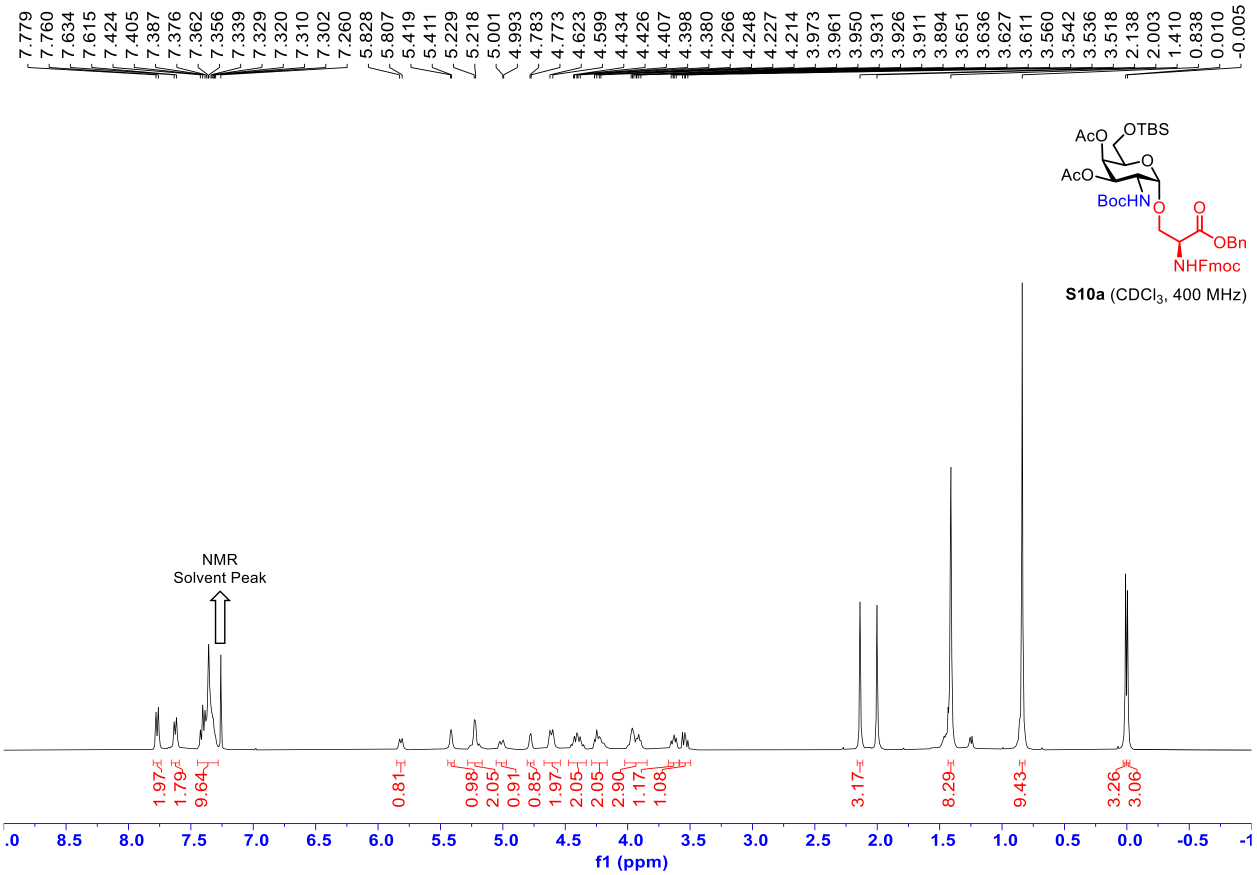
7.779 7.760 7.634 7.615 7.424 7.405 7.387 7.376 7.362 7.356 7.339 7.329 7.320 7.310 7.302 7.260 5.828 5.807 5.419 5.411 5.229 5.218 5.001 4.993 4.783 4.773 4.623 4.599 4.434 4.426 4.407 4.398 4.380 4.266 4.248 4.227 4.214 3.973 3.961 3.950 3.931 3.926 3.911 3.894 3.651 3.636 3.627 3.611 3.560 3.542 3.536 3.518 2.138 2.003 1.410 0.838 0.010 -0.005
AcO
OTBS
O
AcO
BocHN
O
O
OBn
NHFmoc
S10a (CDCl3, 400 MHz)
NMR
Solvent Peak
1.97 1.79 9.64 0.81 0.98 2.05 0.91 0.85 1.97 2.05 2.05 2.90 1.17 1.08 3.17 8.29 9.43 3.26 3.06
f1 (ppm)

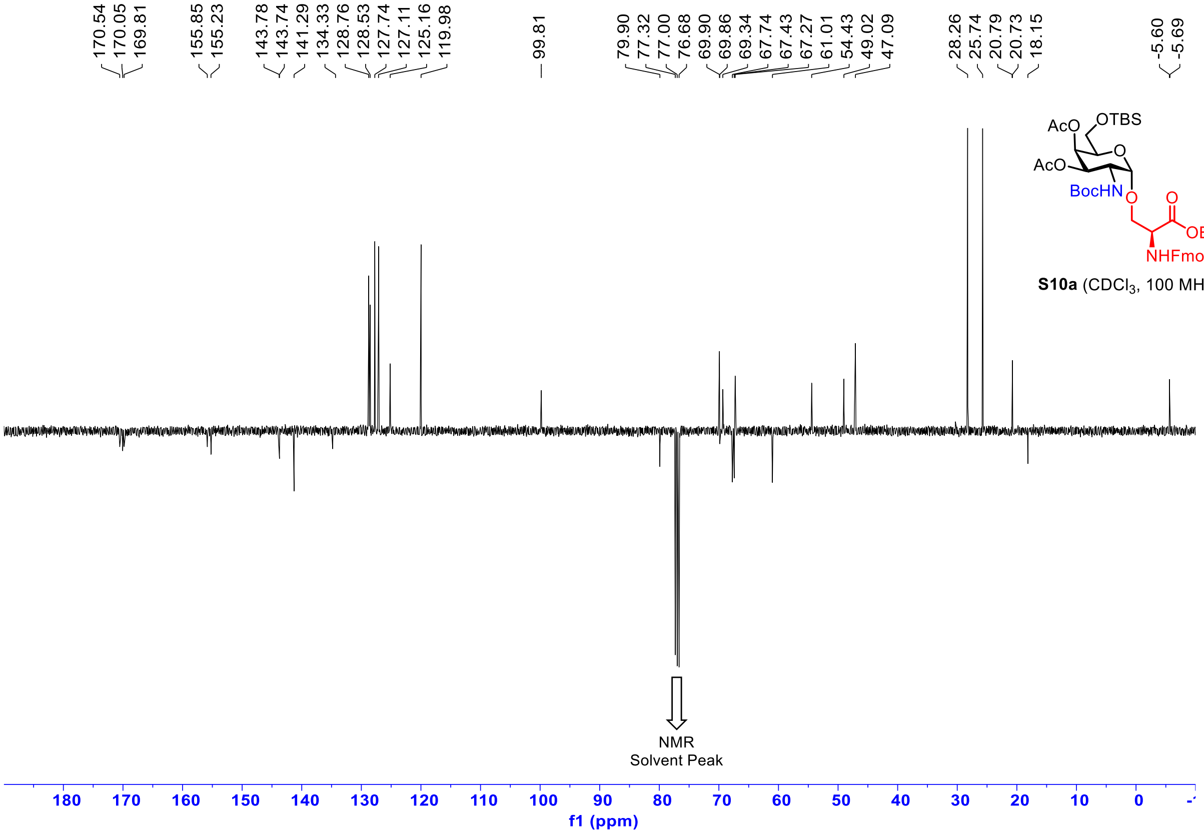
170.54 170.05 169.81 155.85 155.23 143.78 143.74 141.29 134.33 128.76 128.53 127.74 127.11 125.16 119.98 99.81 79.90 77.32 77.00 76.68 69.90 69.86 69.34 67.74 67.43 67.27 61.01 54.43 49.02 47.09 28.26 25.74 20.79 20.73 18.15 -5.60 -5.69
AcO
OTBS
O
AcO
BocHN
O
O
OBn
NHFmoc
S10a (CDCl3, 100 MHz)
NMR
Solvent Peak
f1 (ppm)

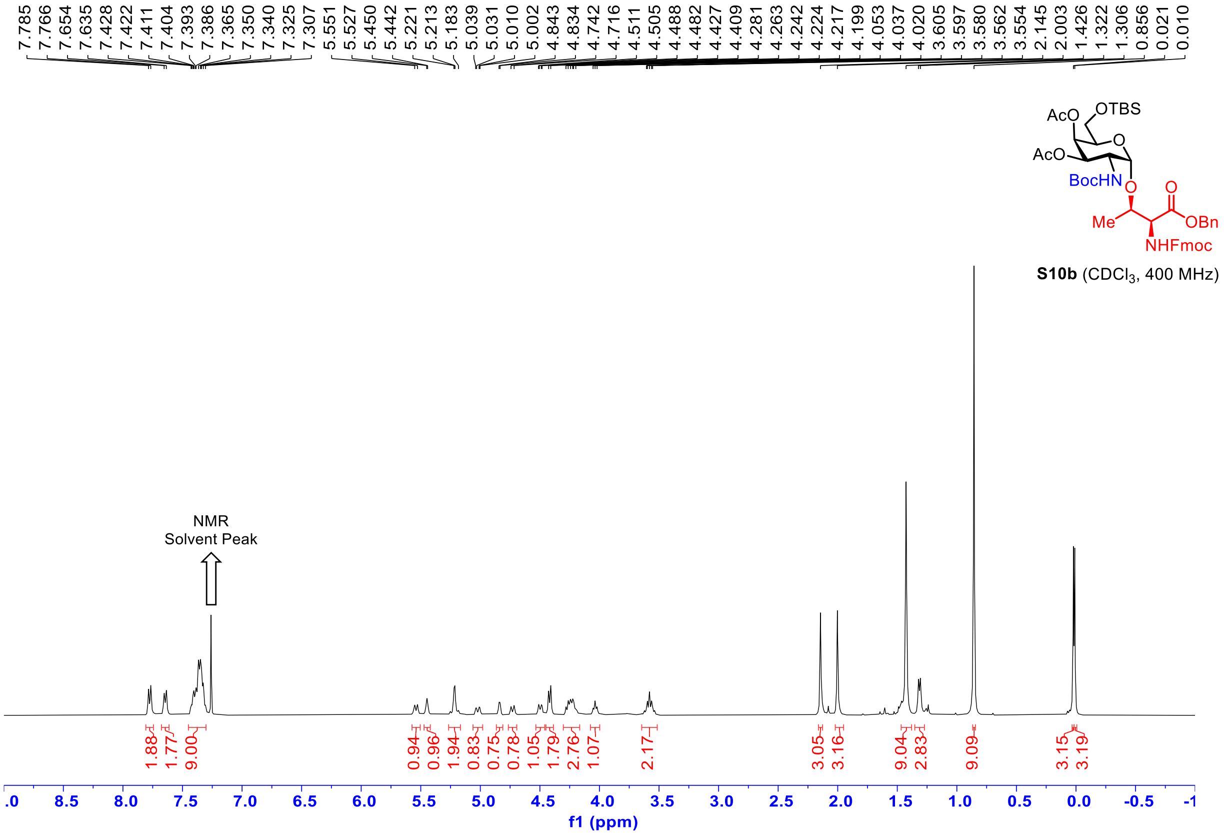
7.785 7.766 7.654 7.635 7.428 7.422 7.411 7.404 7.393 7.386 7.365 7.350 7.340 7.325 7.307 5.551 5.527 5.450 5.442 5.221 5.213 5.183 5.039 5.031 5.010 5.002 4.843 4.834 4.742 4.716 4.511 4.505 4.488 4.482 4.427 4.409 4.281 4.263 4.242 4.224 4.217 4.199 4.053 4.037 4.020 3.605 3.597 3.580 3.562 3.554 2.145 2.003 1.426 1.322 1.306 0.856 0.021 0.010
AcO
OTBS
AcO
BocHN
O
O
Me
O
OBn
NHFmoc
S10b (CDCl3, 400 MHz)
NMR
Solvent Peak
1.88 1.77 9.00 0.94 0.96 1.94 0.83 0.75 0.78 1.05 1.79 2.76 1.07 2.17 3.05 3.16 9.04 2.83 9.09 3.15 3.19
f1 (ppm)

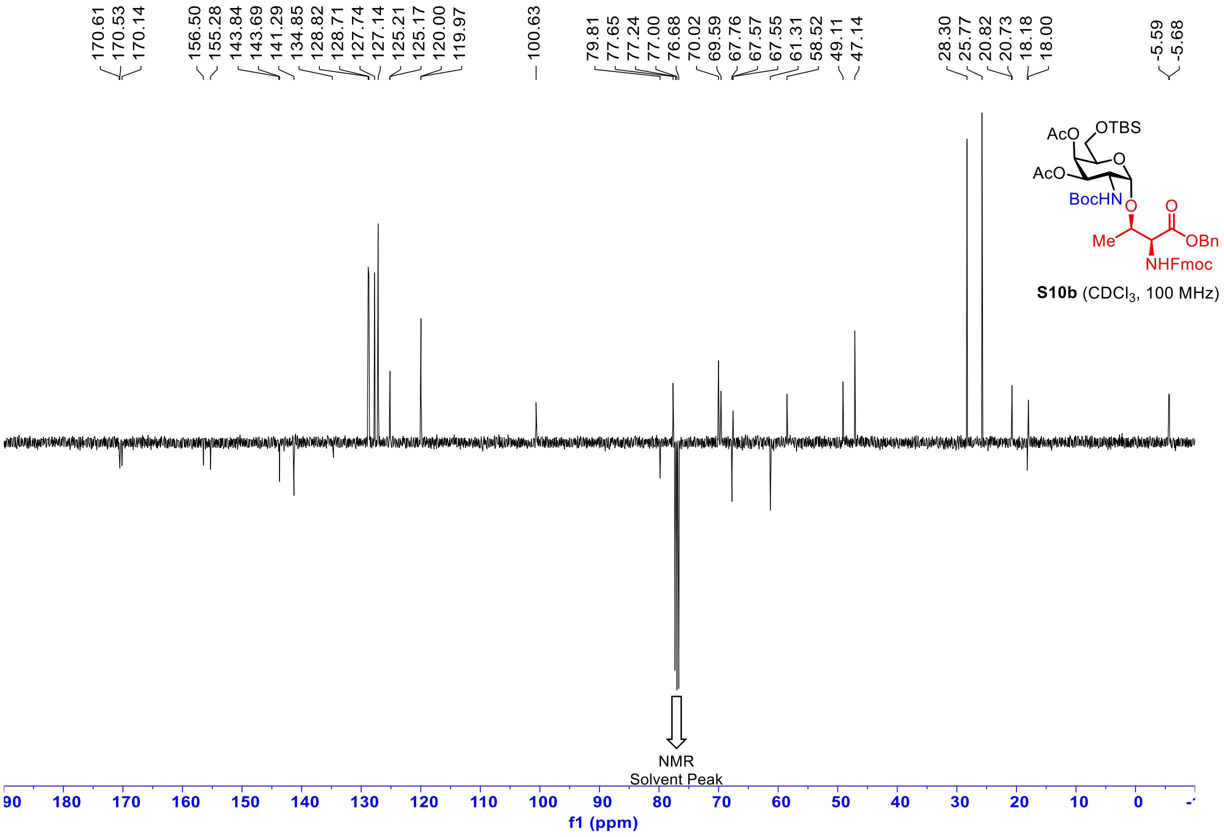
170.61 170.53 170.14 156.50 155.28 143.84 143.69 141.29 134.85 128.82 128.71 127.74 127.14 125.21 125.17 120.00 119.97 100.63 79.81 77.65 77.24 77.00 76.68 70.02 69.59 67.76 67.57 67.55 61.31 58.52 49.11 47.14 28.30 25.77 20.82 20.73 18.18 18.00 -5.59 -5.68
AcO
OTBS
AcO
BocHN
O
O
Me
O
OBn
NHFmoc
S10b (CDCl3, 100 MHz)
NMR
Solvent Peak
f1 (ppm)

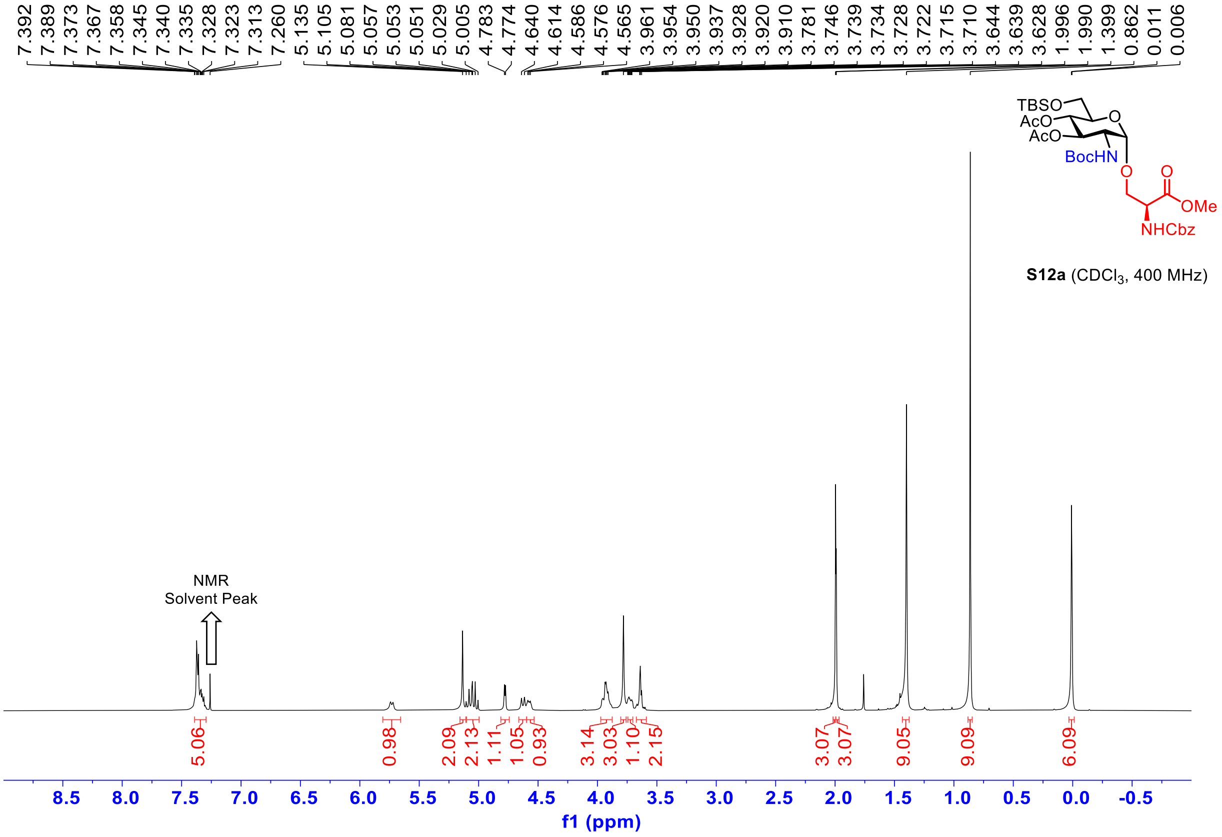
S12a (CDCl3, 400 MHz)
NMR
Solvent Peak
f1 (ppm)

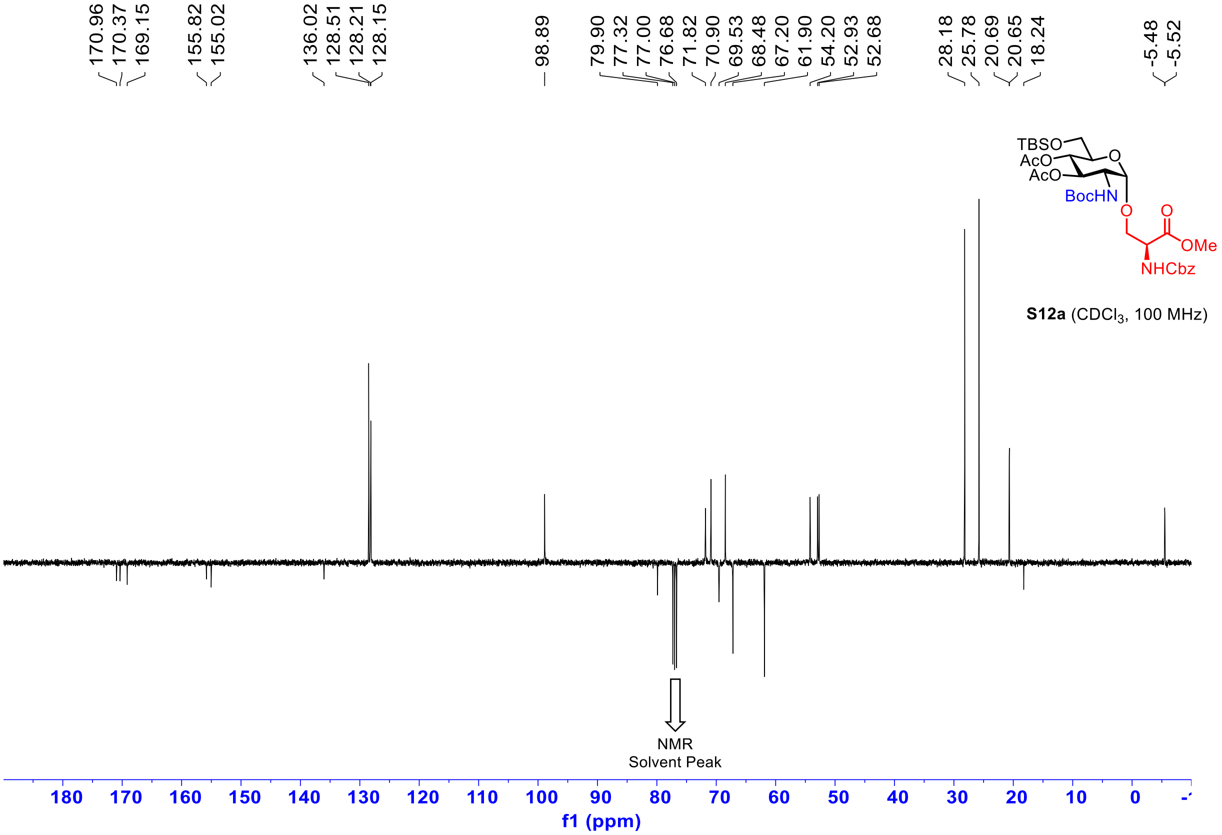
S12a (CDCl3, 100 MHz)
NMR
Solvent Peak
f1 (ppm)

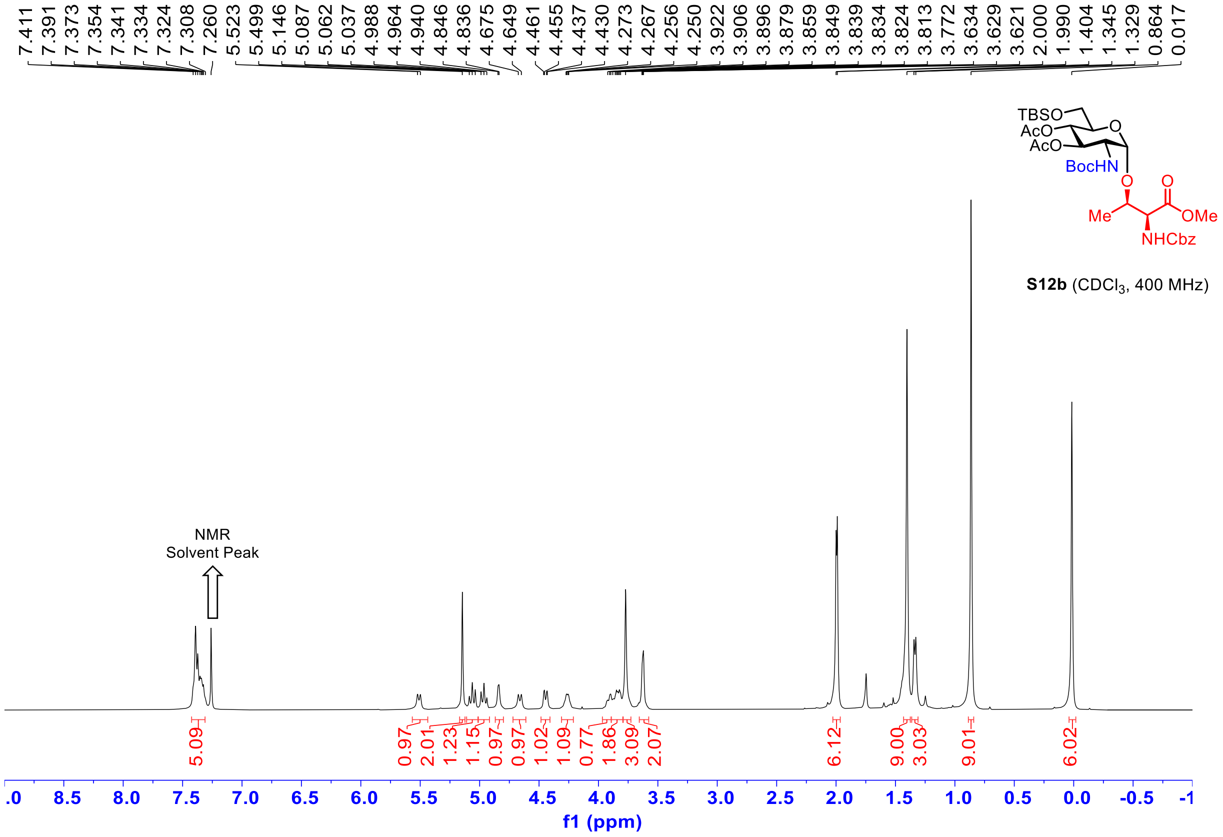
7.411
7.391
7.373
7.354
7.341
7.334
7.324
7.308
7.260
5.523
5.499
5.146
5.087
5.062
5.037
4.988
4.964
4.940
4.846
4.836
4.675
4.649
4.461
4.455
4.437
4.430
4.273
4.267
4.256
4.250
3.922
3.906
3.896
3.879
3.859
3.849
3.839
3.834
3.824
3.813
3.772
3.634
3.629
3.621
2.000
1.990
1.404
1.345
1.329
0.864
0.017
TBSO
AcO
AcO
O
BocHN
O
O
Me
OMe
NHCbz
S12b (CDCl3, 400 MHz)
NMR
Solvent Peak
5.09
0.97
2.01
1.23
1.15
0.97
0.97
1.02
1.09
0.77
1.86
3.09
2.07
6.12
9.00
3.03
9.01
6.02
.0
8.5
8.0
7.5
7.0
6.5
6.0
5.5
5.0
4.5
4.0
3.5
3.0
2.5
2.0
1.5
1.0
0.5
0.0
-0.5
-1
f1 (ppm)

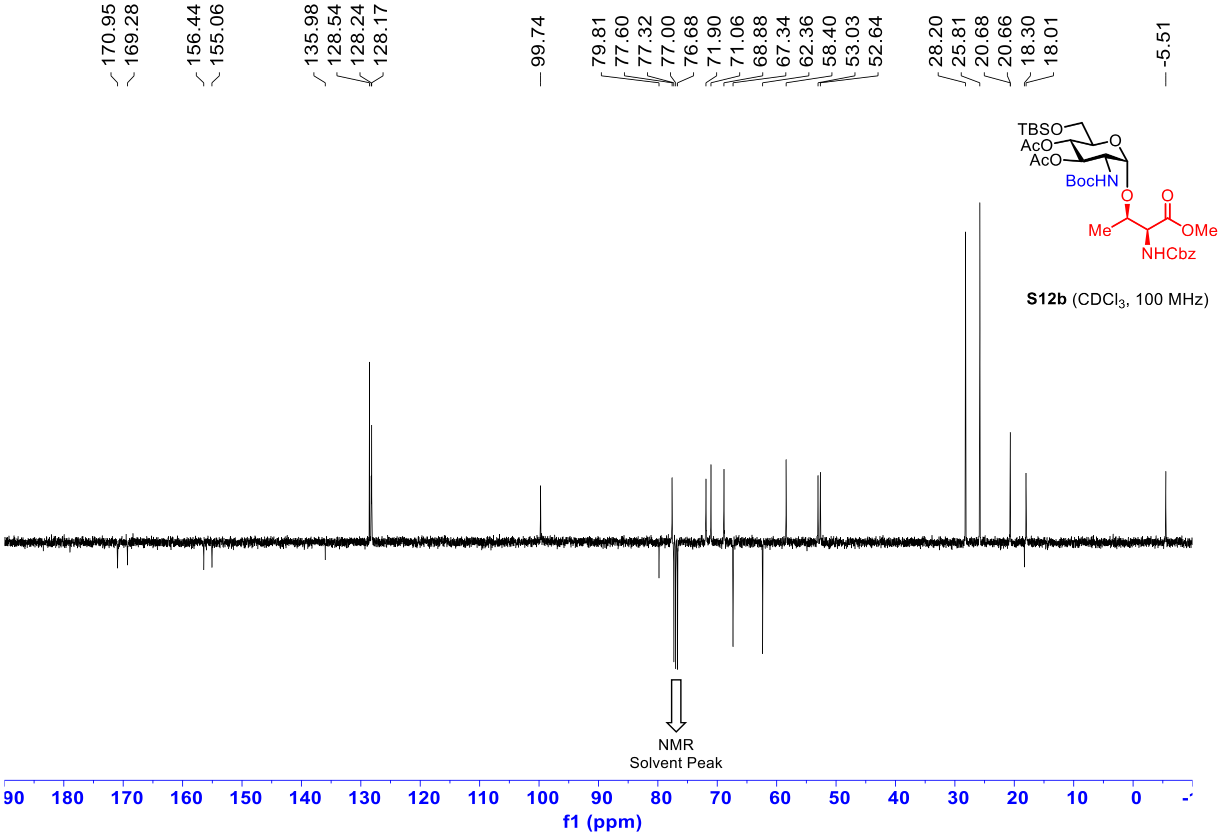
170.95
169.28
156.44
155.06
135.98
128.54
128.24
128.17
99.74
79.81
77.60
77.32
77.00
76.68
71.90
71.06
68.88
67.34
62.36
58.40
53.03
52.64
28.20
25.81
20.68
20.66
18.30
18.01
-5.51
TBSO
AcO
AcO
O
BocHN
O
O
Me
OMe
NHCbz
S12b (CDCl3, 100 MHz)
NMR
Solvent Peak
90
180
170
160
150
140
130
120
110
100
90
80
70
60
50
40
30
20
10
0
f1 (ppm)

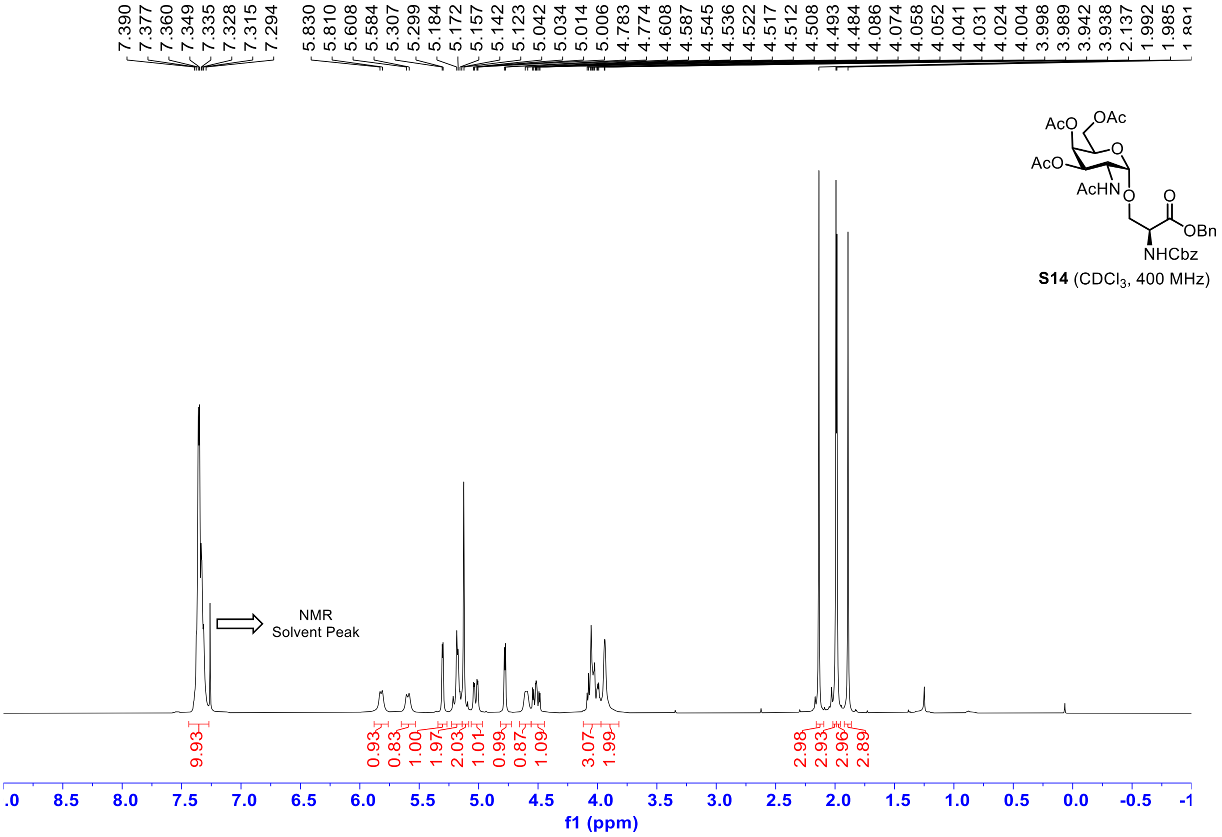
7.390
7.377
7.360
7.349
7.335
7.328
7.315
7.294
5.830
5.810
5.608
5.584
5.307
5.299
5.184
5.172
5.157
5.142
5.123
5.042
5.034
5.014
5.006
4.783
4.774
4.608
4.587
4.545
4.536
4.522
4.517
4.512
4.508
4.493
4.484
4.086
4.074
4.058
4.052
4.041
4.031
4.024
4.004
3.998
3.989
3.942
3.938
2.137
1.992
1.985
AcO
OAc
AcO
AcHN
O
OBn
NHCbz
S14 (CDCl3, 400 MHz)
NMR
Solvent Peak
9.93
0.93
0.83
1.00
1.97
2.03
1.01
0.99
0.87
1.09
3.07
1.99
2.98
2.93
2.96
2.89
f1 (ppm)

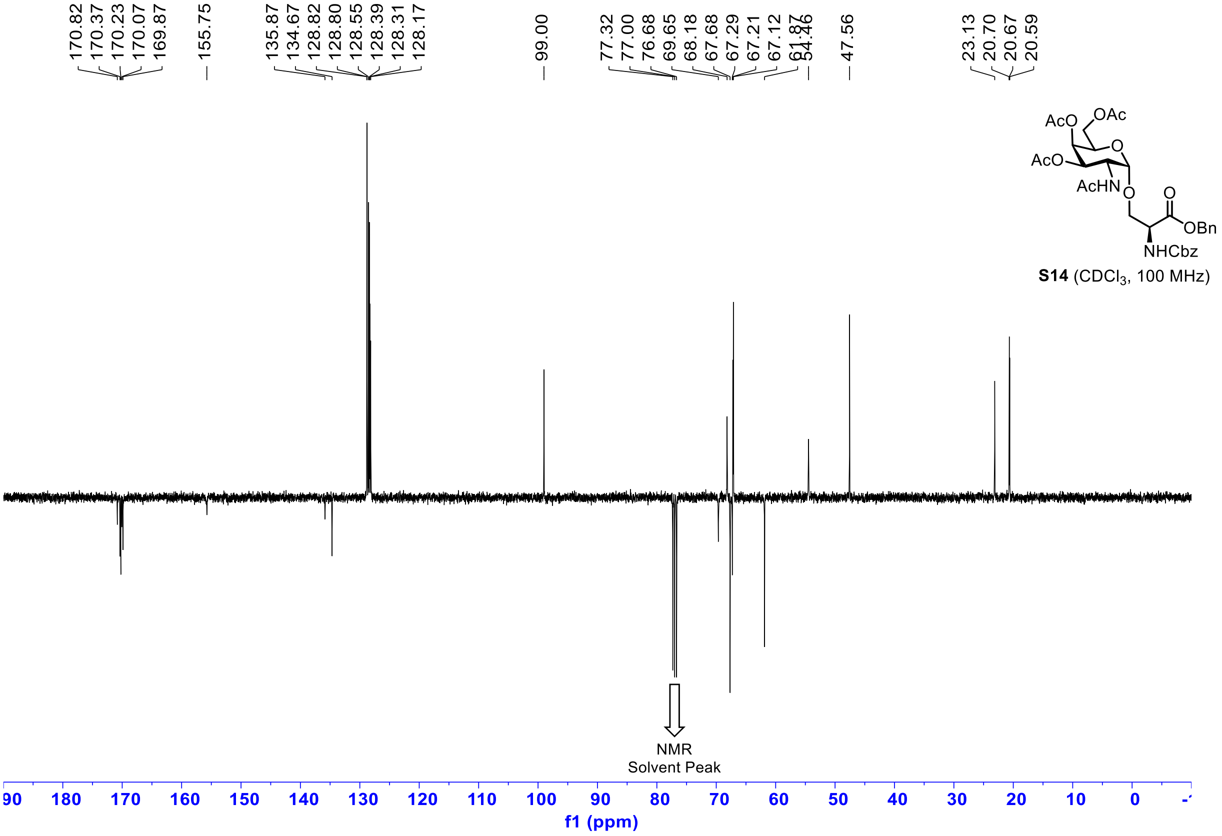
170.82
170.37
170.23
170.07
169.87
155.75
135.87
134.67
128.82
128.80
128.55
128.39
128.31
128.17
99.00
77.32
77.00
76.68
69.65
68.18
67.68
67.29
67.21
67.12
61.87
54.46
47.56
23.13
20.70
20.67
20.59
AcO
OAc
AcO
AcHN
O
OBn
NHCbz
S14 (CDCl3, 100 MHz)
NMR
Solvent Peak
f1 (ppm)

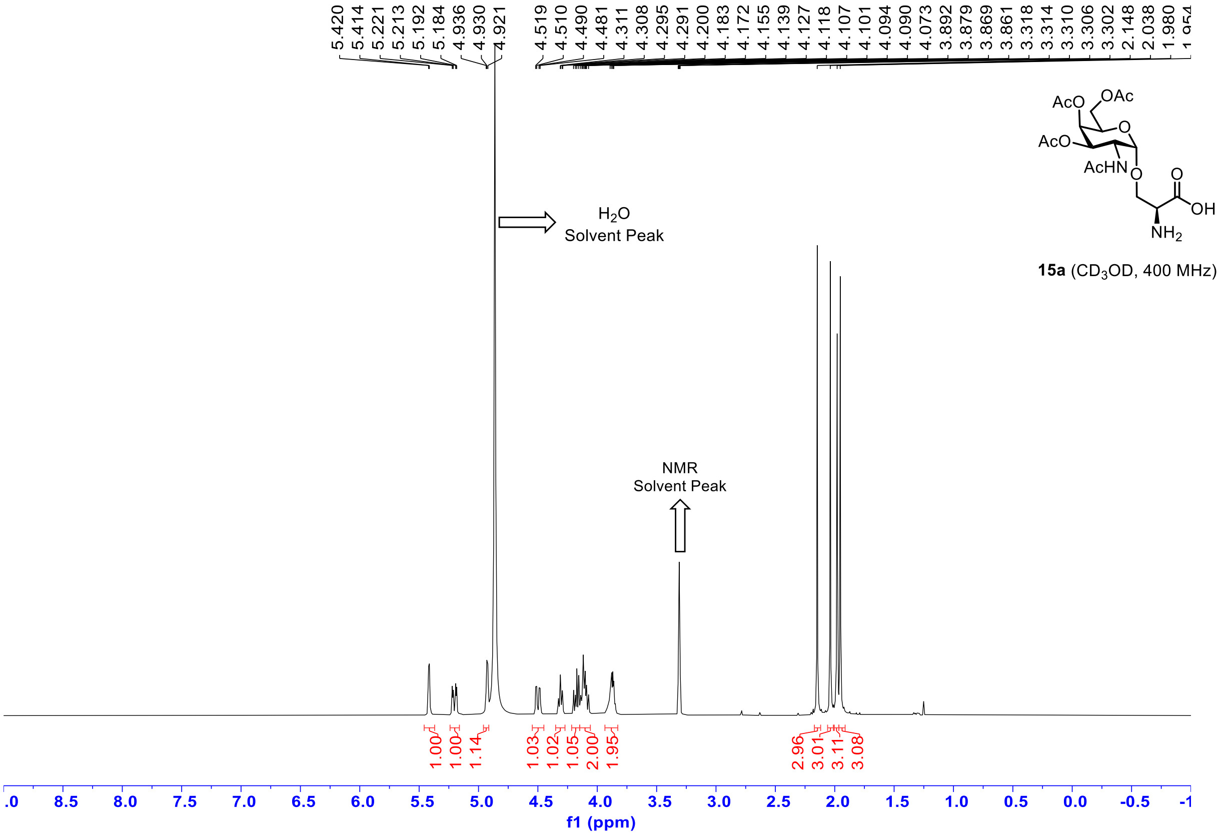
5.420
5.414
5.221
5.213
5.192
5.184
4.936
4.930
4.921
4.519
4.510
4.490
4.481
4.311
4.308
4.295
4.291
4.200
4.183
4.172
4.155
4.139
4.127
4.118
4.107
4.101
4.094
4.090
4.073
3.892
3.879
3.869
3.861
3.318
3.314
3.310
3.306
3.302
2.148
2.038
1.980
AcO
OAc
O
AcO
AcHN
O
O
OH
NH_2
15a (CD_3OD, 400 MHz)
H_2O
Solvent Peak
NMR
Solvent Peak
1.00
1.00
1.14
1.03
1.02
1.05
2.00
1.95
2.96
3.01
3.11
3.08
8.5
8.0
7.5
7.0
6.5
6.0
5.5
5.0
4.5
4.0
3.5
3.0
2.5
2.0
1.5
1.0
0.5
0.0
-0.5
f1 (ppm)

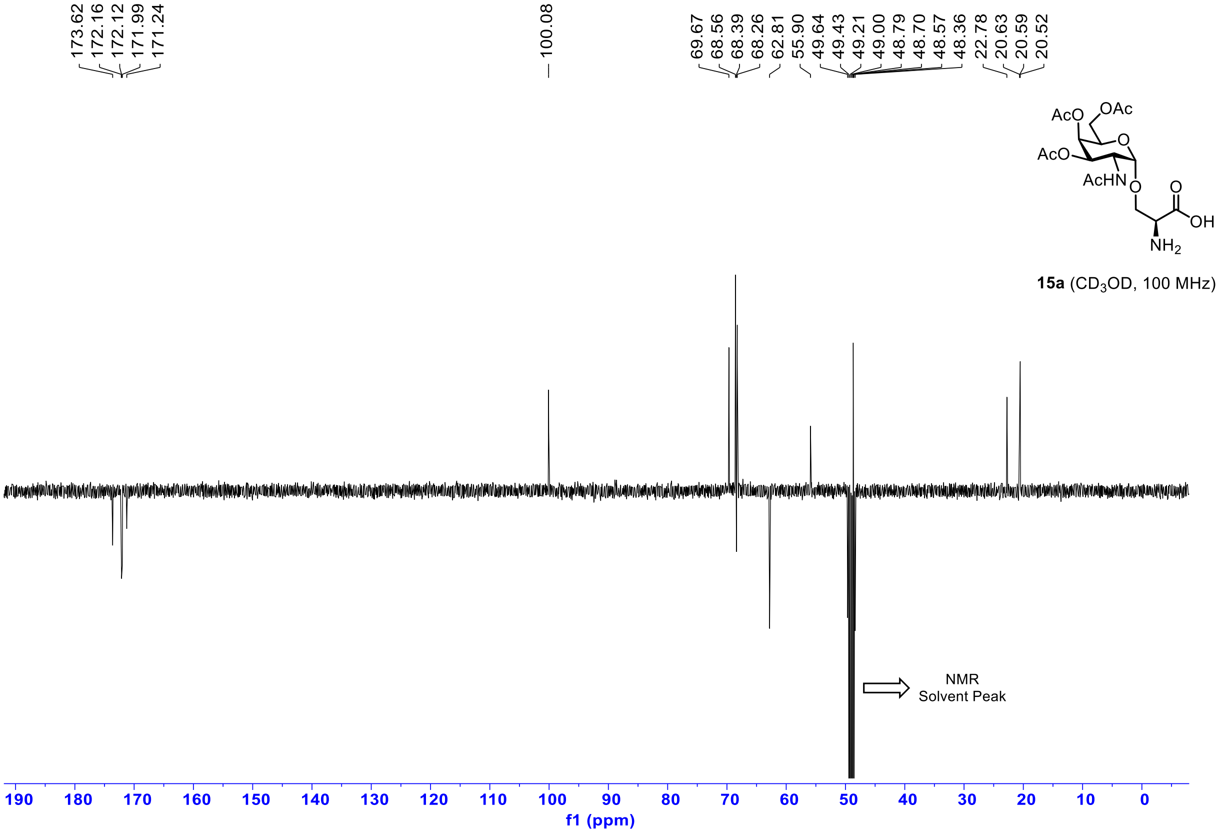
173.62
172.16
172.12
171.99
171.24
100.08
69.67
68.56
68.39
68.26
62.81
55.90
49.64
49.43
49.21
49.00
48.79
48.70
48.57
48.36
22.78
20.63
20.59
20.52
AcO
OAc
O
AcO
AcHN
O
O
OH
NH_2
15a (CD_3OD, 100 MHz)
NMR
Solvent Peak
190
180
170
160
150
140
130
120
110
100
90
80
70
60
50
40
30
20
10
0
f1 (ppm)

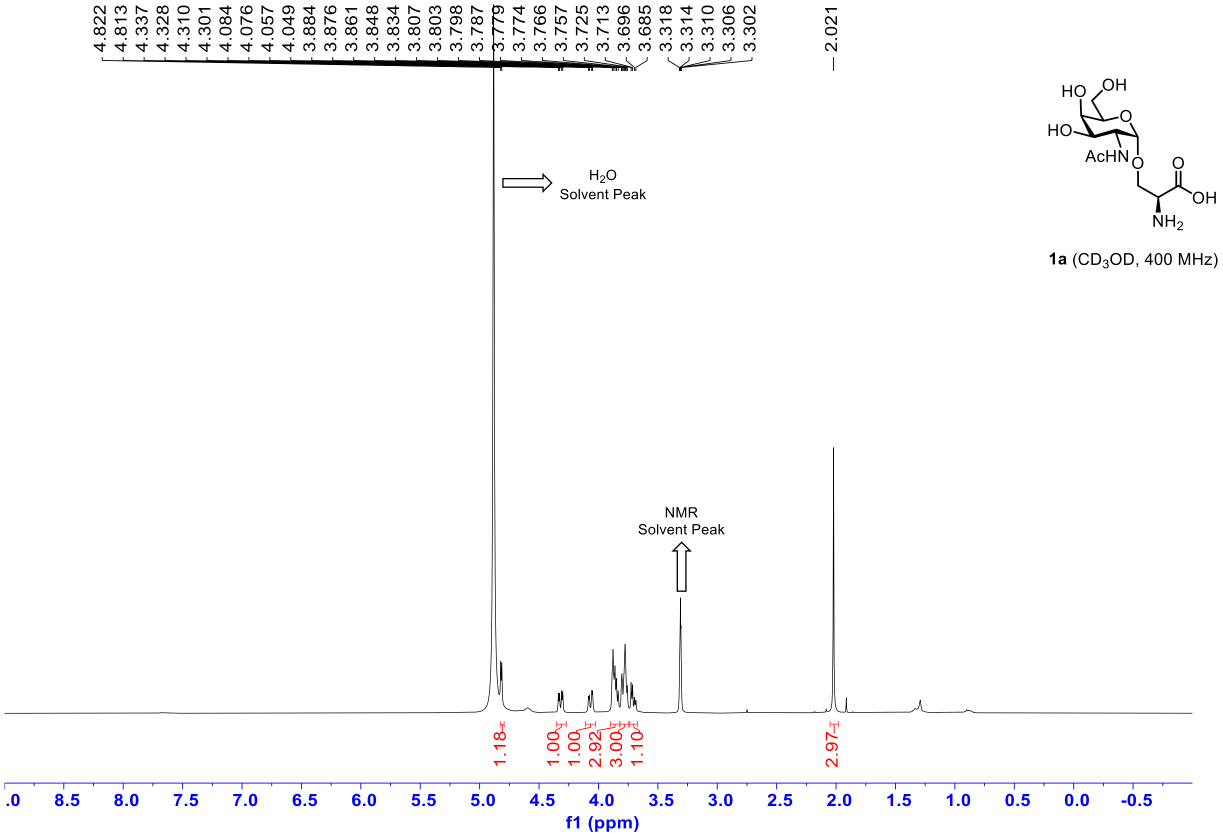

1a (CD_3OD, 400 MHz)

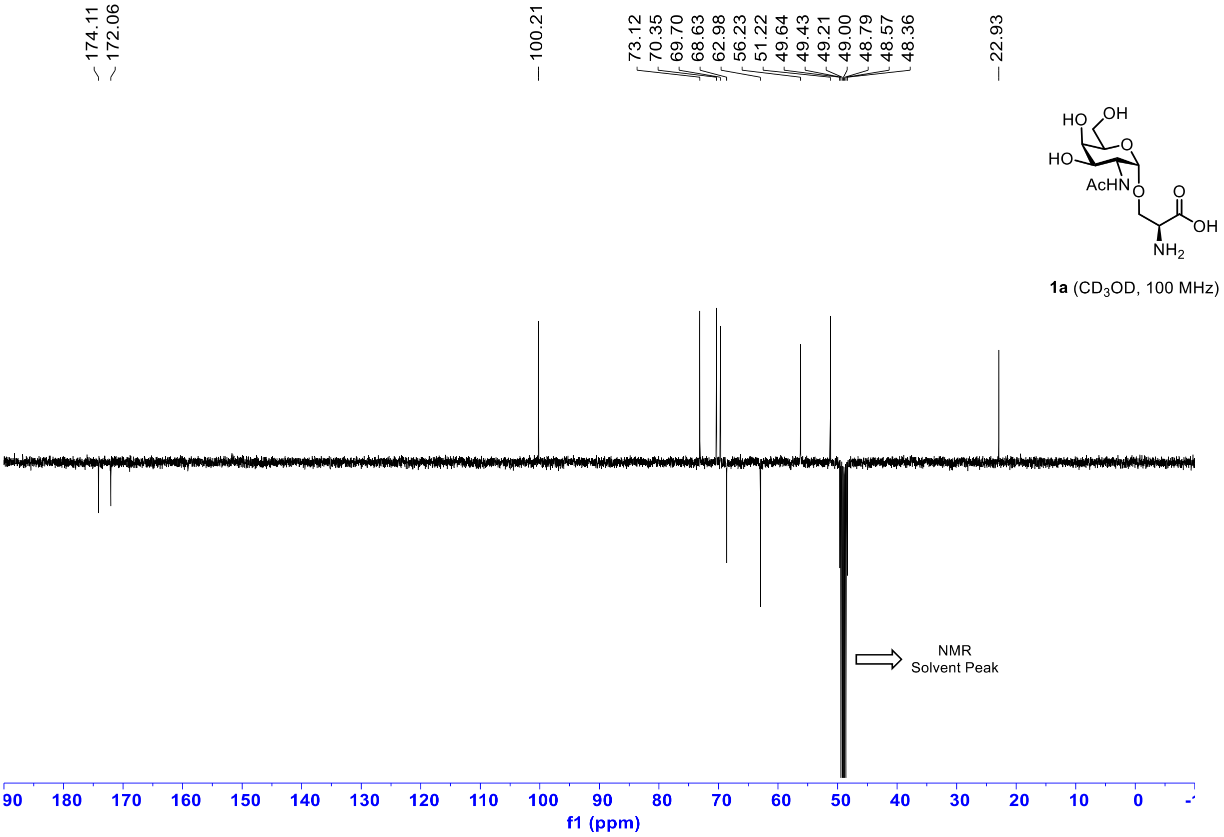

1a (CD_3OD, 100 MHz)

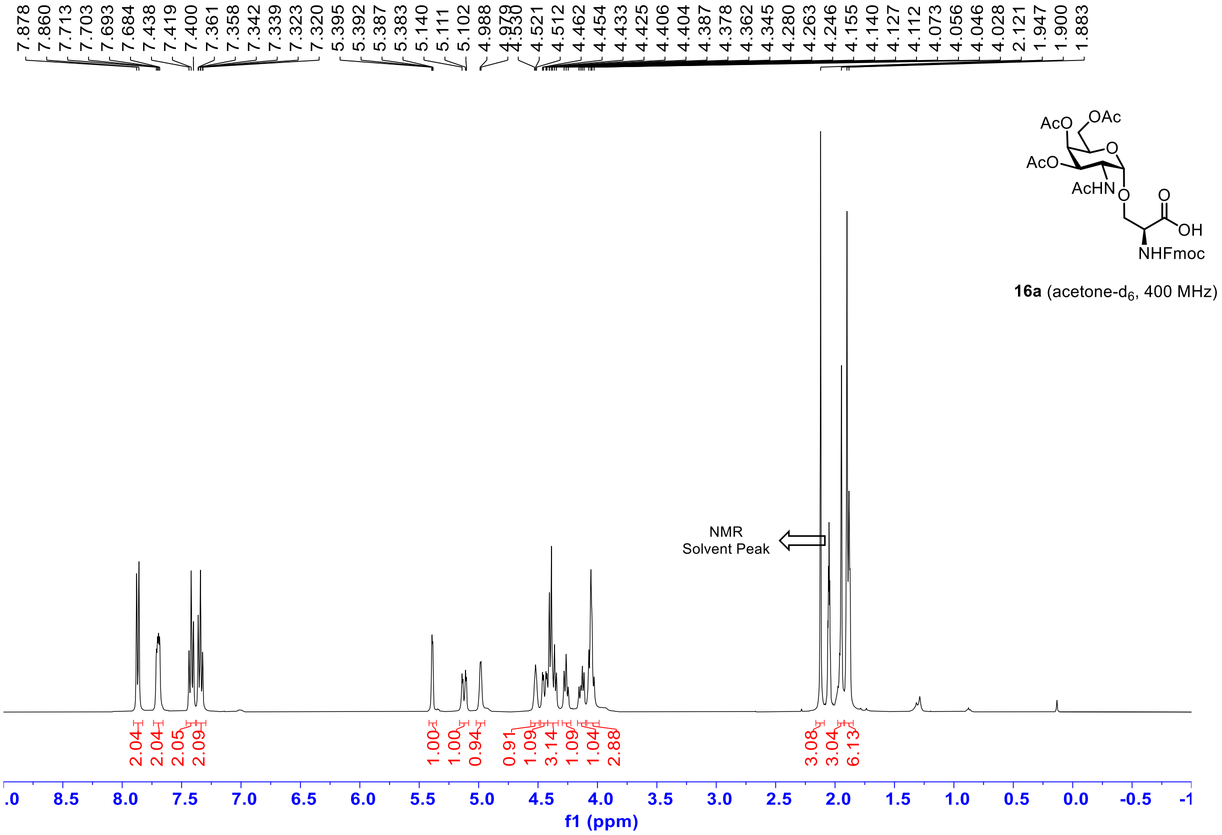
7.878 7.860 7.713 7.703 7.693 7.684 7.438 7.419 7.400 7.361 7.358 7.342 7.339 7.323 7.320 5.395 5.392 5.387 5.383 5.140 5.111 5.102 4.988 4.979 4.530 4.521 4.512 4.462 4.454 4.433 4.425 4.406 4.404 4.387 4.378 4.362 4.345 4.280 4.263 4.246 4.155 4.140 4.127 4.112 4.073 4.056 4.046 4.028 2.121 1.947 1.900 1.883
AcO
OAc
AcO
AcHN
O
OH
NHFmoc
16a (acetone-d6, 400 MHz)
NMR
Solvent Peak
2.04 2.04 2.05 2.09 1.00 1.00 0.94 0.91 1.09 3.14 1.09 1.04 2.88 3.08 3.04 6.13
.0 8.5 8.0 7.5 7.0 6.5 6.0 5.5 5.0 4.5 4.0 3.5 3.0 2.5 2.0 1.5 1.0 0.5 0.0 -0.5 -1
f1 (ppm)

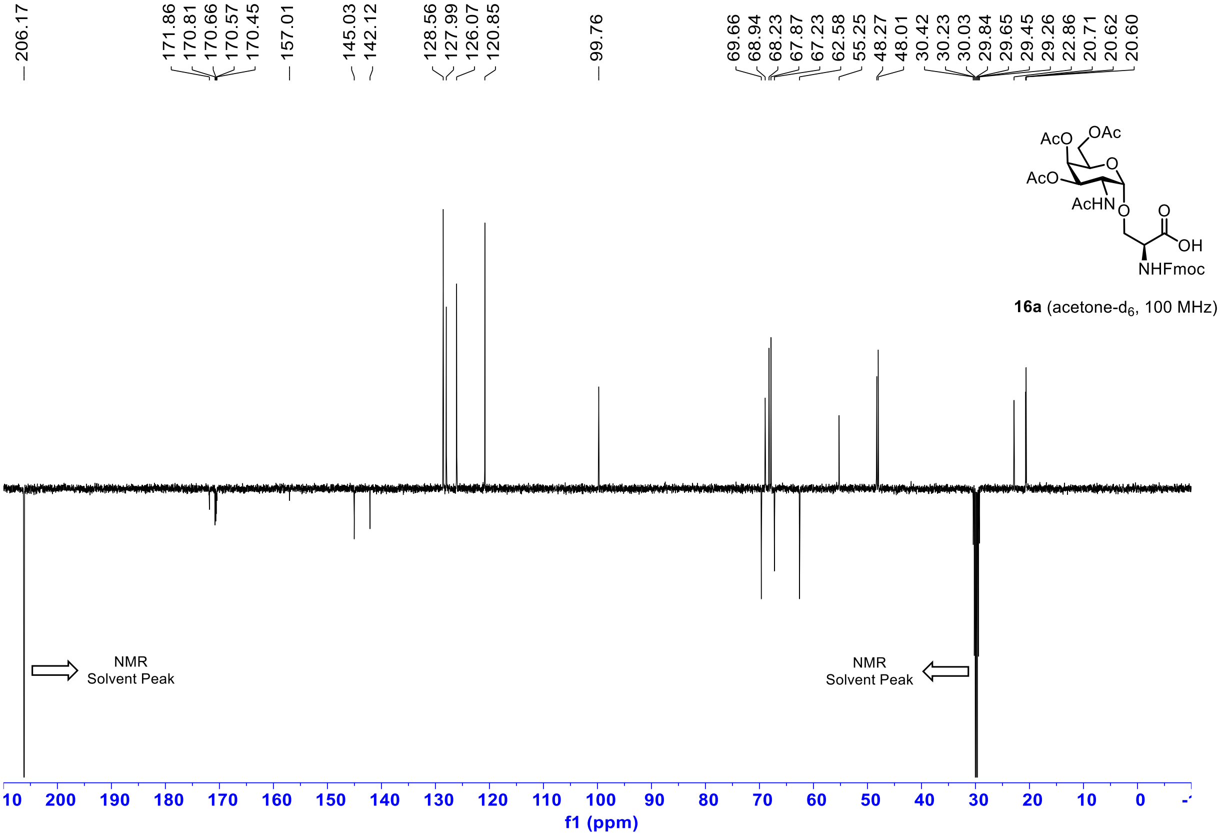
206.17 171.86 170.81 170.66 170.57 170.45 157.01 145.03 142.12 128.56 127.99 126.07 120.85 99.76 69.66 68.94 68.23 67.87 67.23 62.58 55.25 48.27 48.01 30.42 30.23 30.03 29.84 29.65 29.45 29.26 22.86 20.71 20.62 20.60
AcO
OAc
AcO
AcHN
O
OH
NHFmoc
16a (acetone-d6, 100 MHz)
NMR
Solvent Peak
NMR
Solvent Peak
10 200 190 180 170 160 150 140 130 120 110 100 90 80 70 60 50 40 30 20 10 0
f1 (ppm)

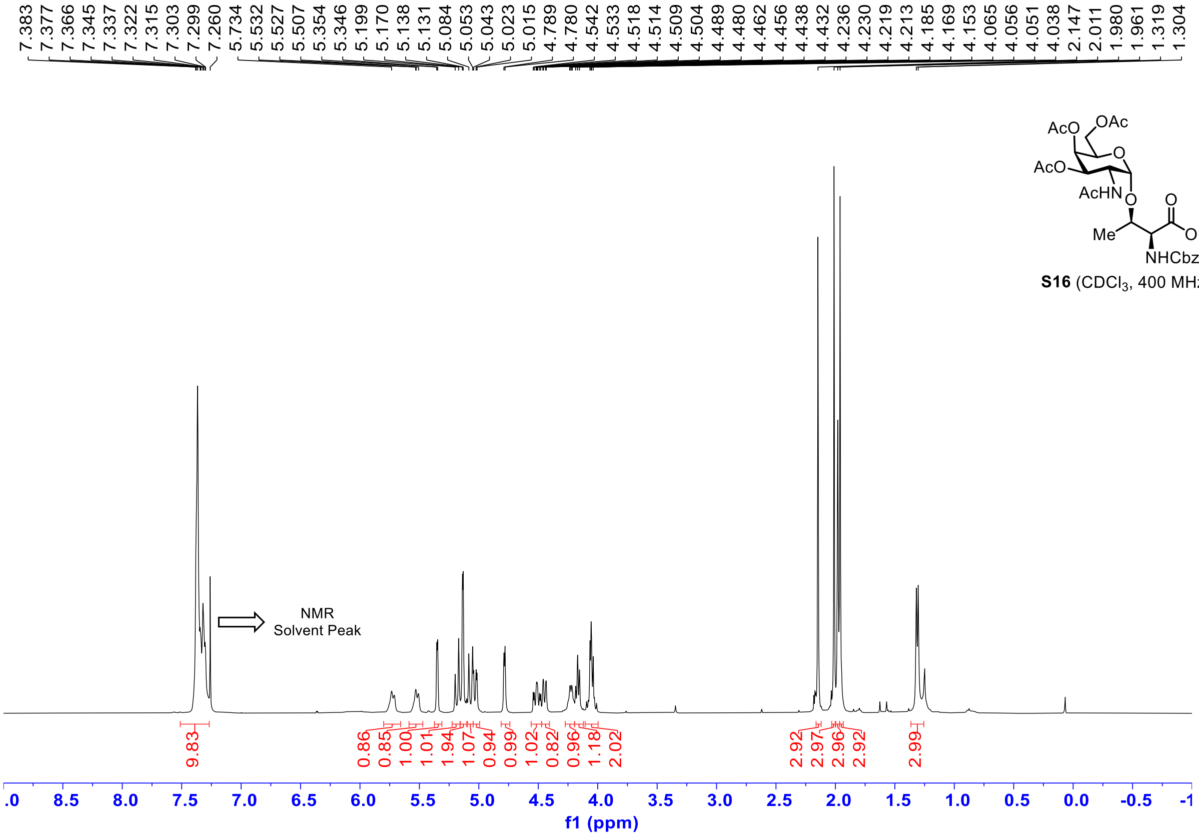
7.383 7.377 7.366 7.345 7.337 7.322 7.315 7.303 7.299 7.260 5.734 5.532 5.527 5.507 5.354 5.346 5.199 5.170 5.138 5.131 5.084 5.053 5.043 5.023 5.015 4.789 4.780 4.542 4.533 4.518 4.514 4.509 4.504 4.489 4.480 4.462 4.456 4.438 4.432 4.236 4.230 4.219 4.213 4.185 4.169 4.153 4.065 4.056 4.051 4.038 2.147 2.011 1.980 1.961 1.319 1.304
AcO
OAc
O
AcO
AcHN
O
O
Me
OBn
NHCbz
S16 (CDCl3, 400 MHz)
NMR
Solvent Peak
9.83
0.86 0.85 1.00 1.01 1.94 1.07 0.94 0.99 1.02 0.82 0.96 1.18 2.02
2.92 2.97 2.96 2.92
2.99
f1 (ppm)

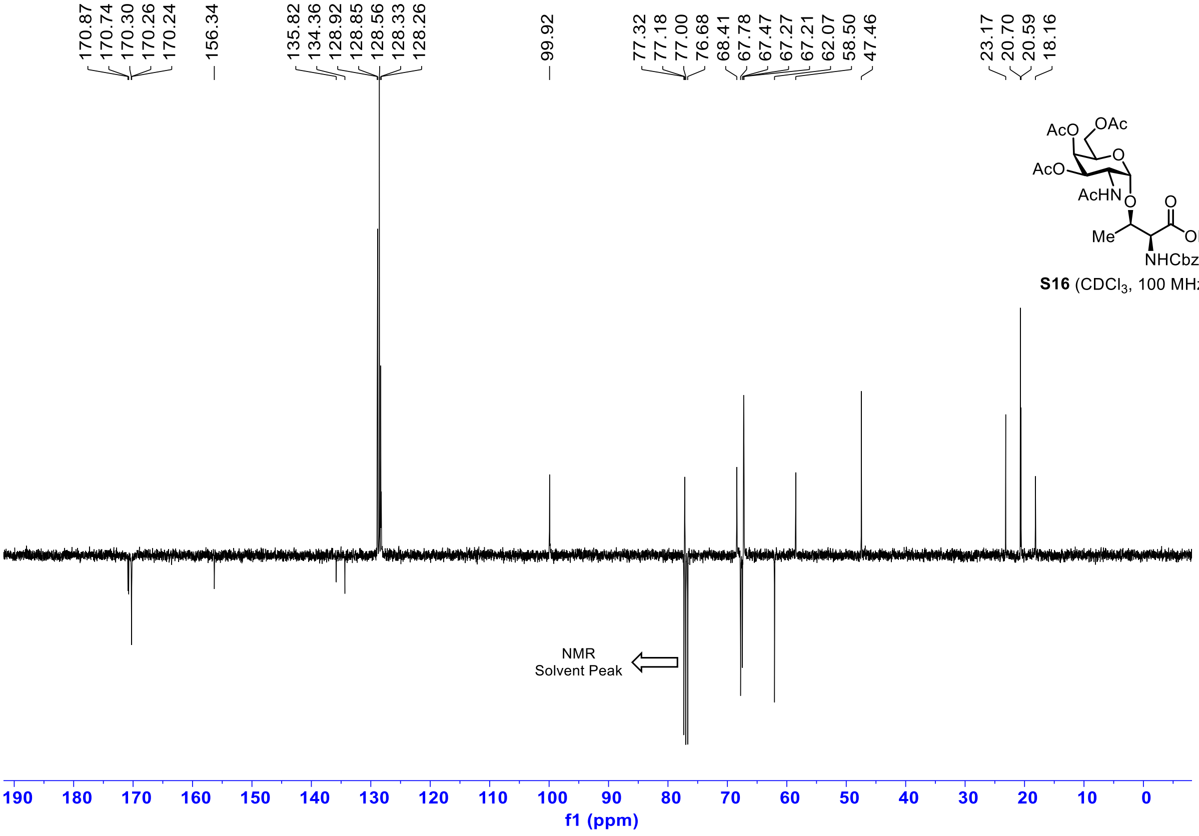
170.87 170.74 170.30 170.26 170.24 156.34
135.82 134.36 128.92 128.85 128.56 128.33 128.26
99.92
77.32 77.18 77.00 76.68 68.41 67.78 67.47 67.27 67.21 62.07 58.50 47.46
23.17 20.70 20.59 18.16
AcO
OAc
O
AcO
AcHN
O
O
Me
OBn
NHCbz
S16 (CDCl3, 100 MHz)
NMR
Solvent Peak
f1 (ppm)

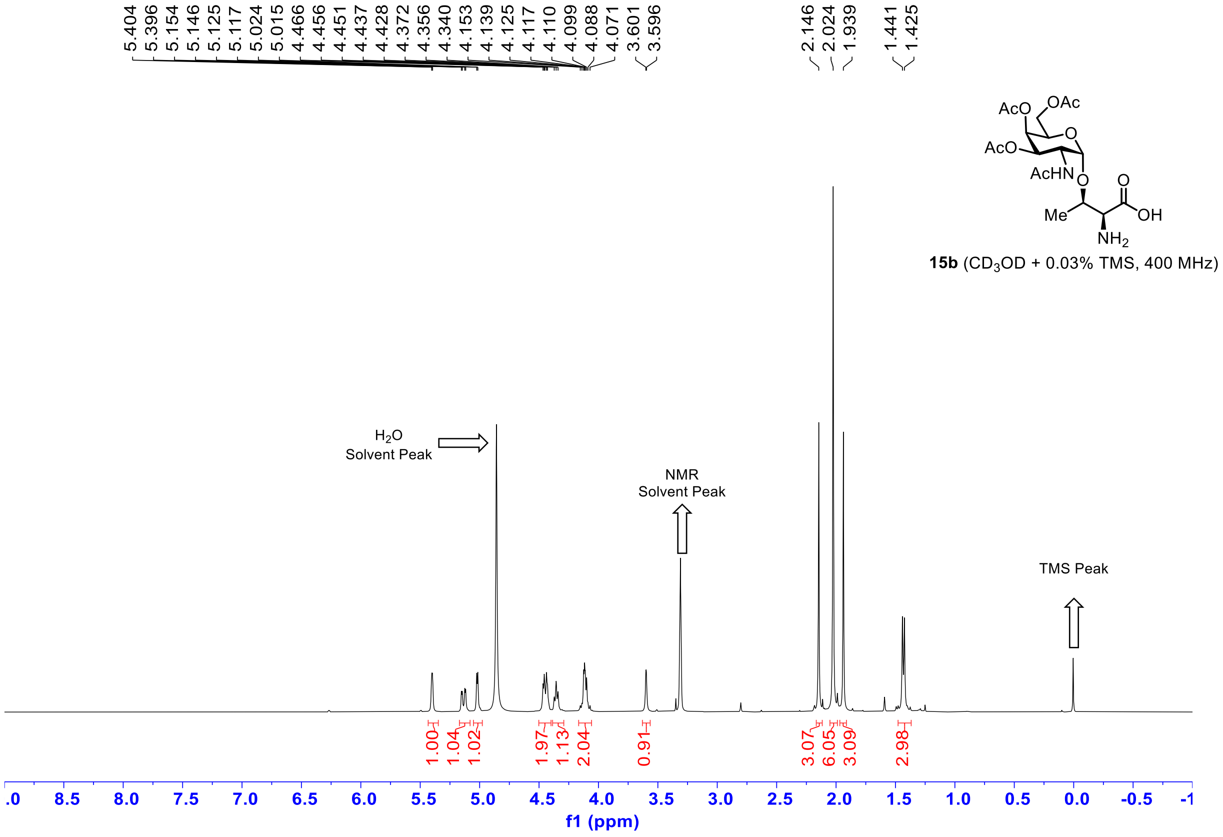
5.404
5.396
5.154
5.146
5.125
5.117
5.024
5.015
4.466
4.456
4.451
4.437
4.428
4.372
4.356
4.340
4.153
4.139
4.125
4.117
4.110
4.099
4.088
4.071
3.601
3.596
2.146
2.024
1.939
1.441
1.425
15b (CD3OD + 0.03% TMS, 400 MHz)
H2O
Solvent Peak
NMR
Solvent Peak
TMS Peak
f1 (ppm)

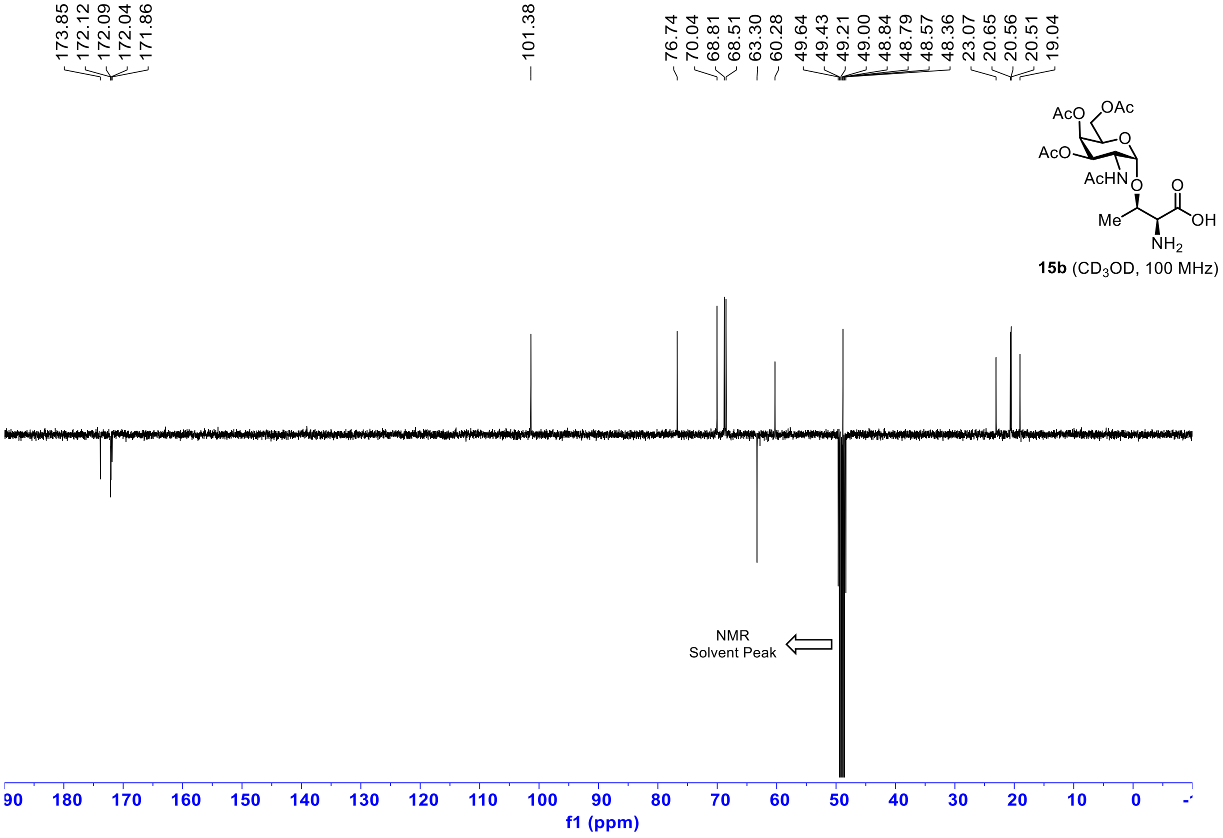
173.85
172.12
172.09
172.04
171.86
101.38
76.74
70.04
68.81
68.51
63.30
60.28
49.64
49.43
49.21
49.00
48.84
48.79
48.57
48.36
23.07
20.65
20.56
20.51
19.04
15b (CD3OD, 100 MHz)
NMR
Solvent Peak
f1 (ppm)

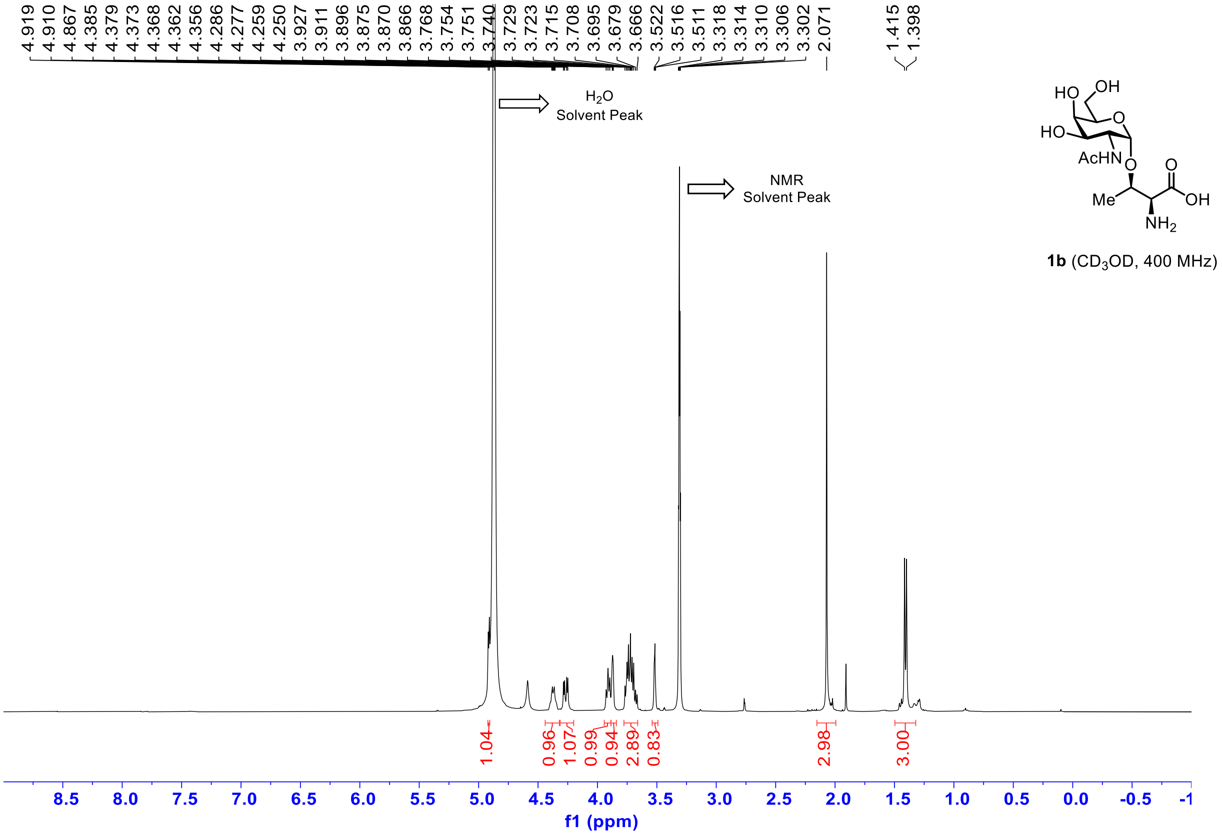

1b (CD_3OD, 400 MHz)

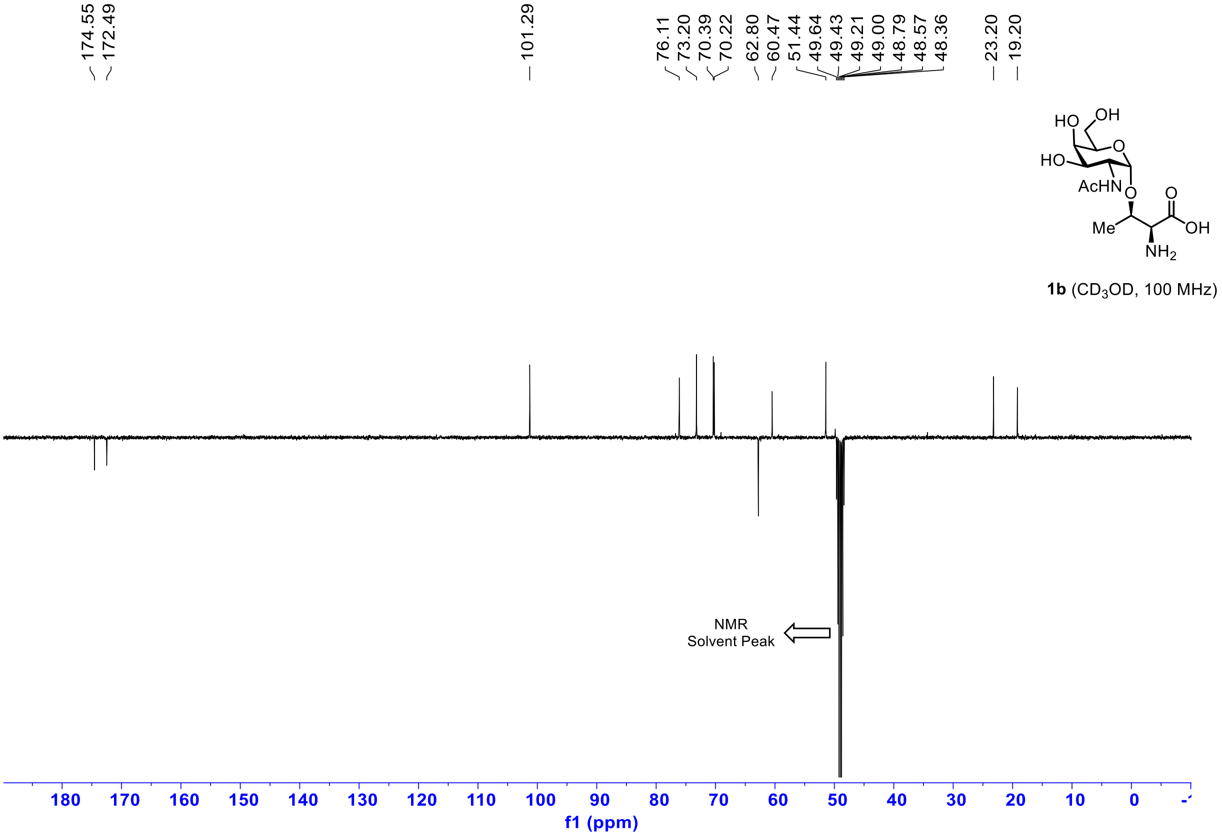

1b (CD_3OD, 100 MHz)

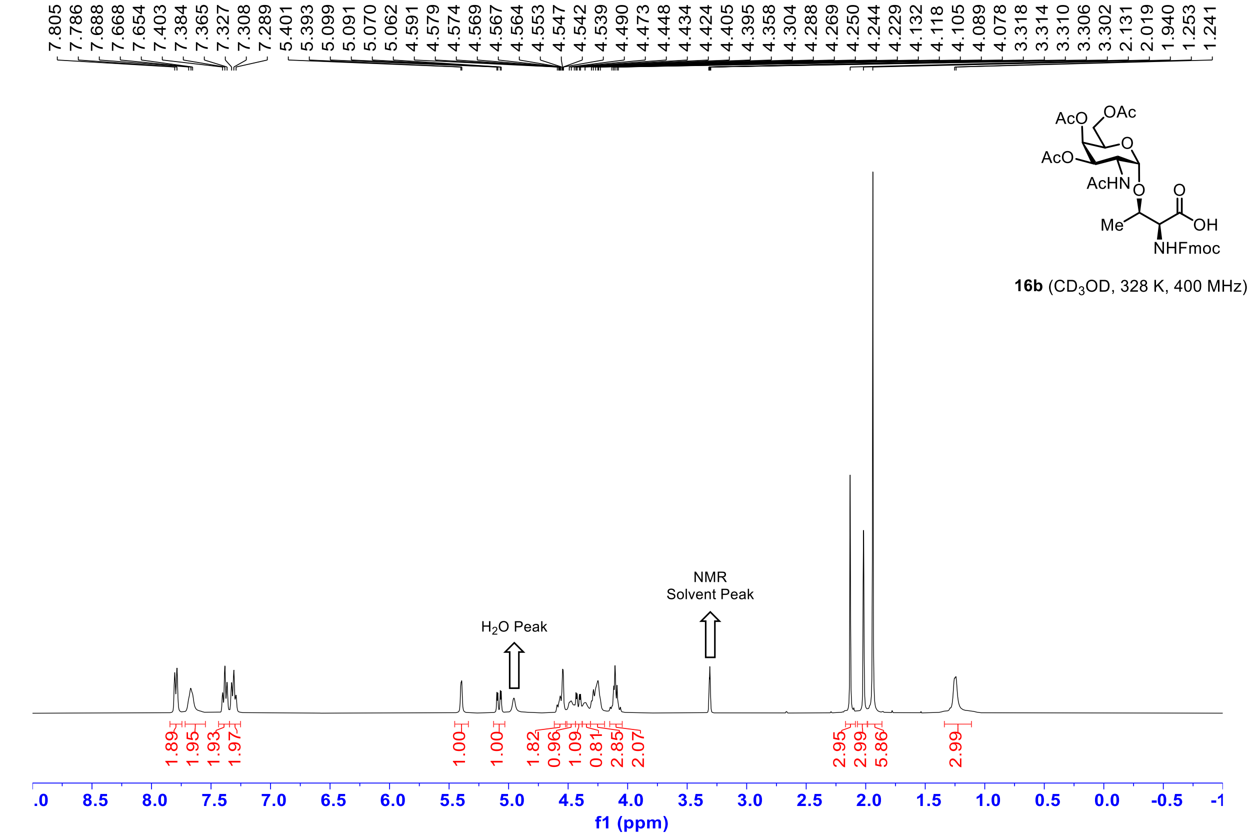
7.805 7.786 7.688 7.668 7.654 7.403 7.384 7.365 7.327 7.308 7.289 5.401 5.393 5.099 5.091 5.070 5.062 4.591 4.579 4.574 4.569 4.567 4.564 4.553 4.547 4.542 4.539 4.490 4.473 4.448 4.434 4.424 4.405 4.395 4.358 4.304 4.288 4.269 4.250 4.244 4.229 4.132 4.118 4.105 4.089 4.078 3.318 3.314 3.310 3.306 3.302 2.131 2.019 1.940 1.253 1.241
AcO
OAc
AcO
O
AcHN
O
O
OH
Me
NHFmoc
16b (CD3OD, 328 K, 400 MHz)
H2O Peak
NMR
Solvent Peak
1.89 1.95 1.93 1.97 1.00 1.00 1.82 0.96 1.09 0.81 2.85 2.07 2.95 2.99 5.86 2.99
f1 (ppm)

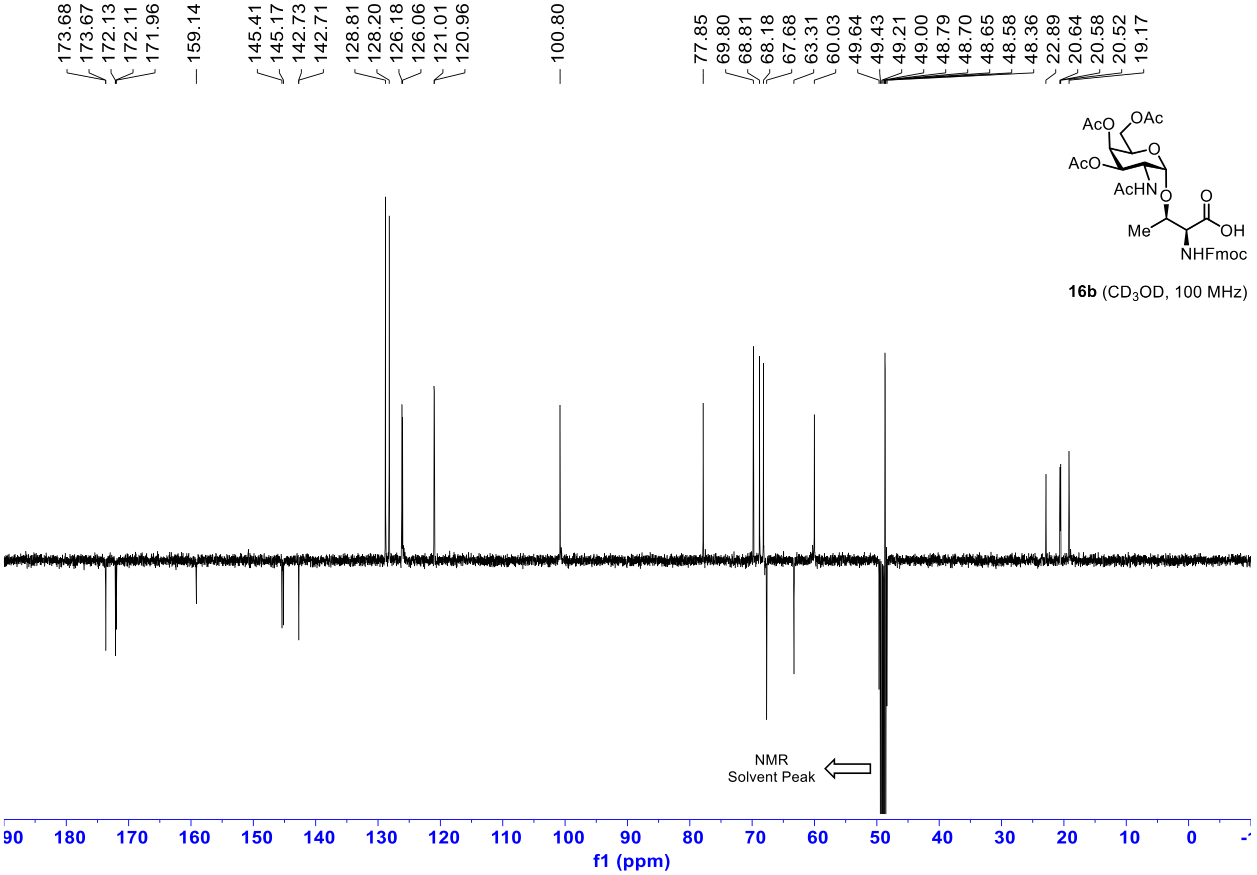
173.68 173.67 172.13 172.11 171.96 159.14 145.41 145.17 142.73 142.71 128.81 128.20 126.18 126.06 121.01 120.96 100.80 77.85 69.80 68.81 68.18 67.68 63.31 60.03 49.64 49.43 49.21 49.00 48.79 48.70 48.65 48.58 48.36 22.89 20.64 20.58 20.52 19.17
AcO
OAc
AcO
O
AcHN
O
O
OH
Me
NHFmoc
16b (CD3OD, 100 MHz)
NMR
Solvent Peak
f1 (ppm)

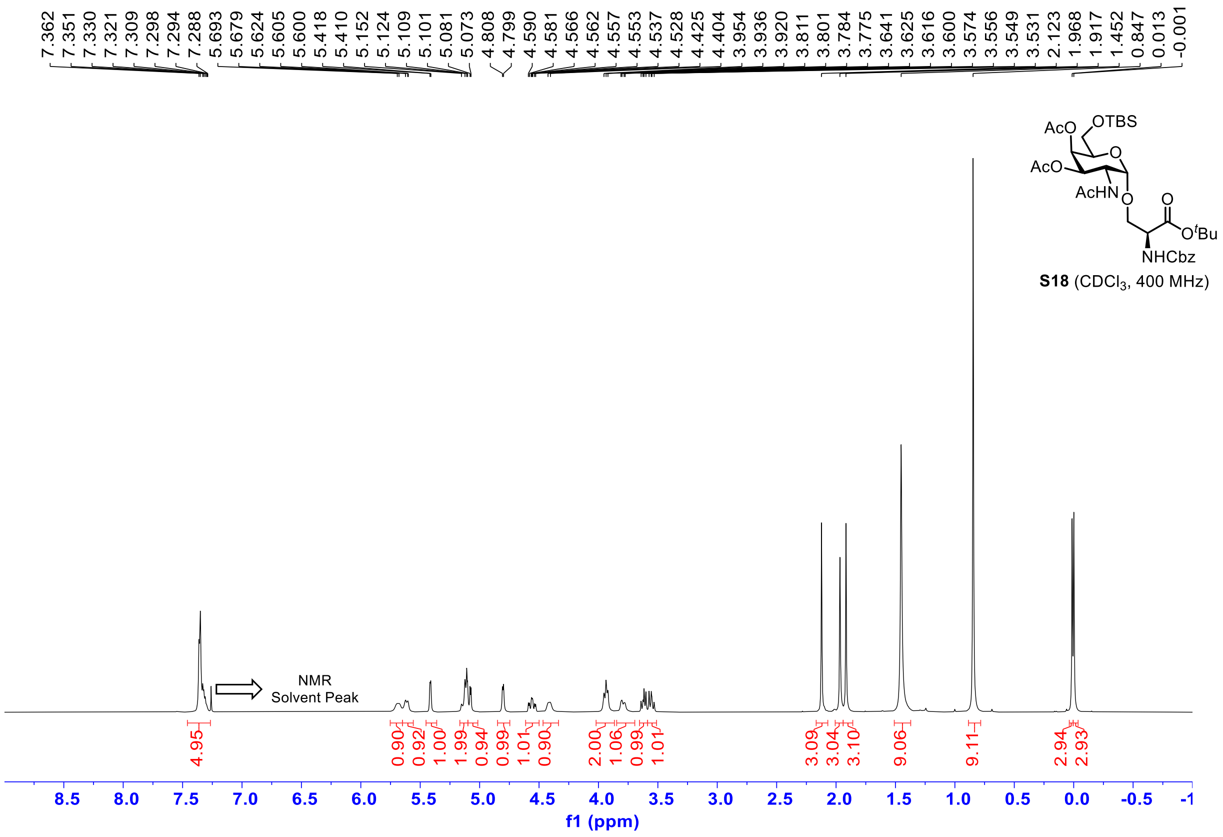
7.362 7.351 7.330 7.321 7.309 7.298 7.294 7.288 5.693 5.679 5.624 5.605 5.600 5.418 5.410 5.152 5.124 5.109 5.101 5.081 5.073 4.808 4.799 4.590 4.581 4.566 4.562 4.557 4.553 4.537 4.528 4.425 4.404 3.954 3.936 3.920 3.811 3.801 3.784 3.775 3.641 3.625 3.616 3.600 3.574 3.556 3.549 3.531 2.123 1.968 1.917 1.452 0.847 0.013 -0.001
AcO
OTBS
AcO
AcHN
O
O
O
O^{t}Bu
NHCbz
S18 (CDCl$_3$, 400 MHz)
NMR
Solvent Peak
4.95 0.90 0.92 1.00 1.99 0.94 0.99 1.01 0.90 2.00 1.06 0.99 1.01 3.09 3.04 3.10 9.06 9.11 2.94 2.93
f1 (ppm)

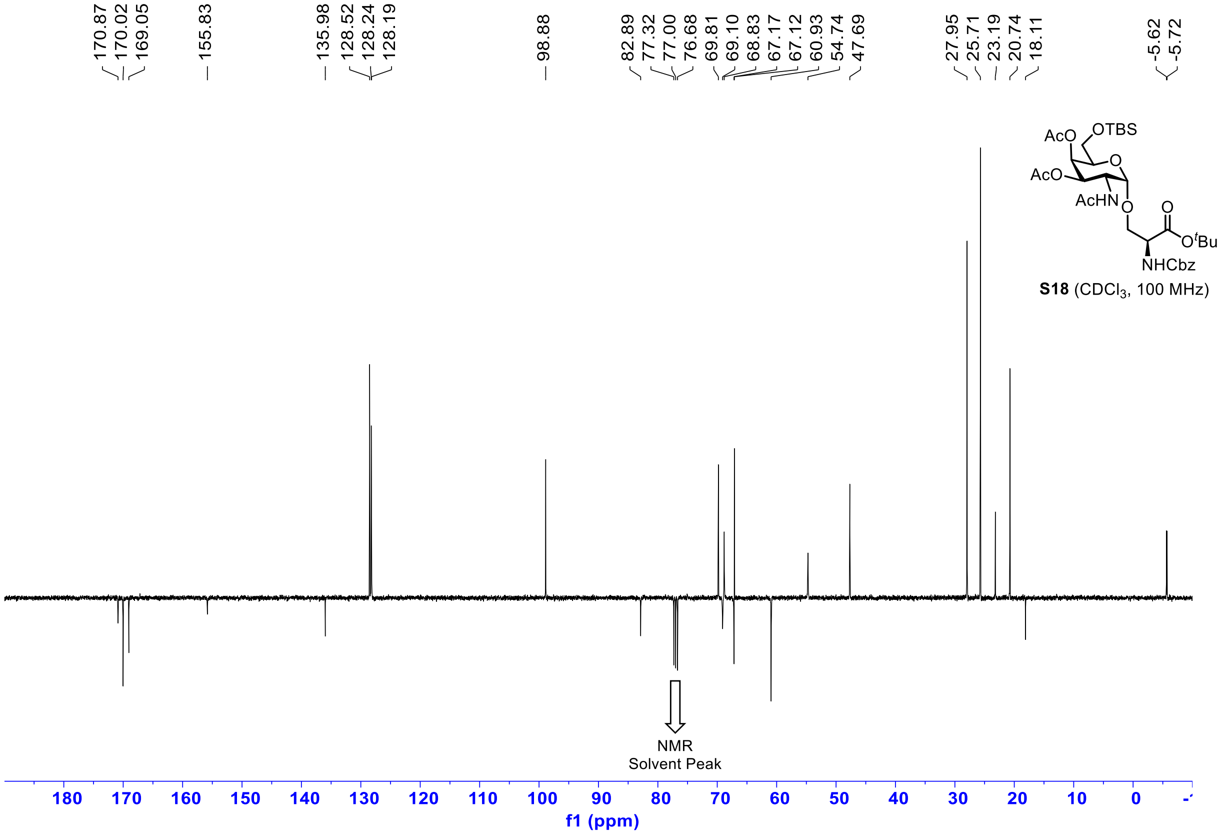
170.87 170.02 169.05 155.83 135.98 128.52 128.24 128.19 98.88 82.89 77.32 77.00 76.68 69.81 69.10 68.83 67.17 67.12 60.93 54.74 47.69 27.95 25.71 23.19 20.74 18.11 -5.62 -5.72
AcO
OTBS
AcO
AcHN
O
O
O
O^{t}Bu
NHCbz
S18 (CDCl$_3$, 100 MHz)
NMR
Solvent Peak
f1 (ppm)

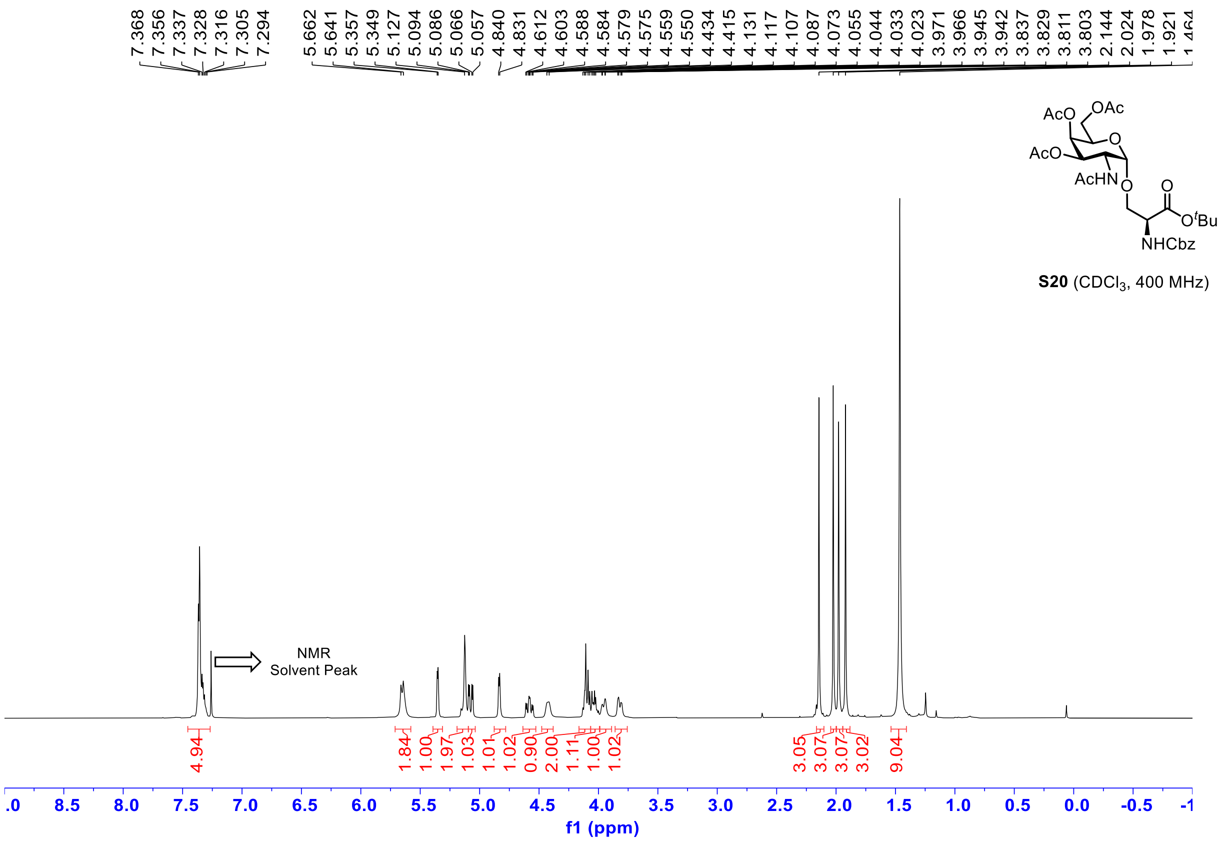
NMR
Solvent Peak
S20 (CDCl$_3$, 400 MHz)
f1 (ppm)

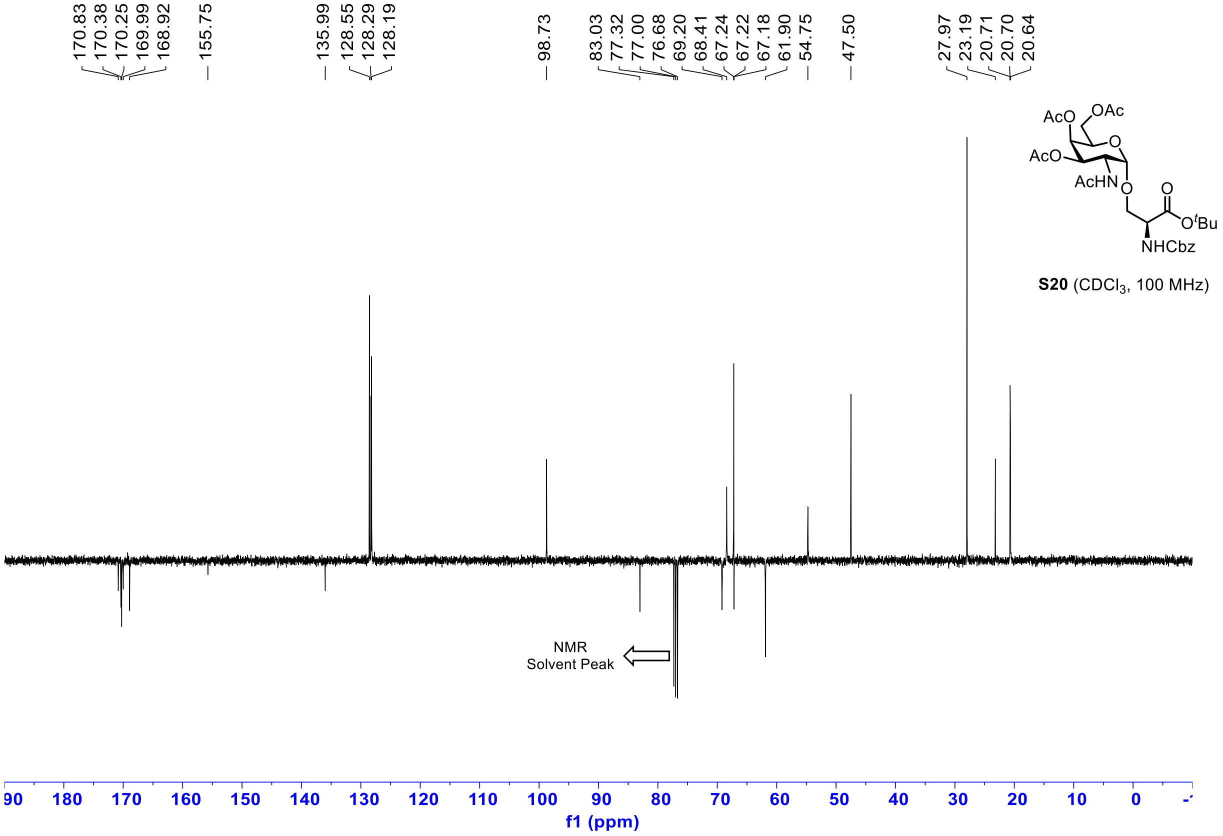
NMR
Solvent Peak
S20 (CDCl$_3$, 100 MHz)
f1 (ppm)

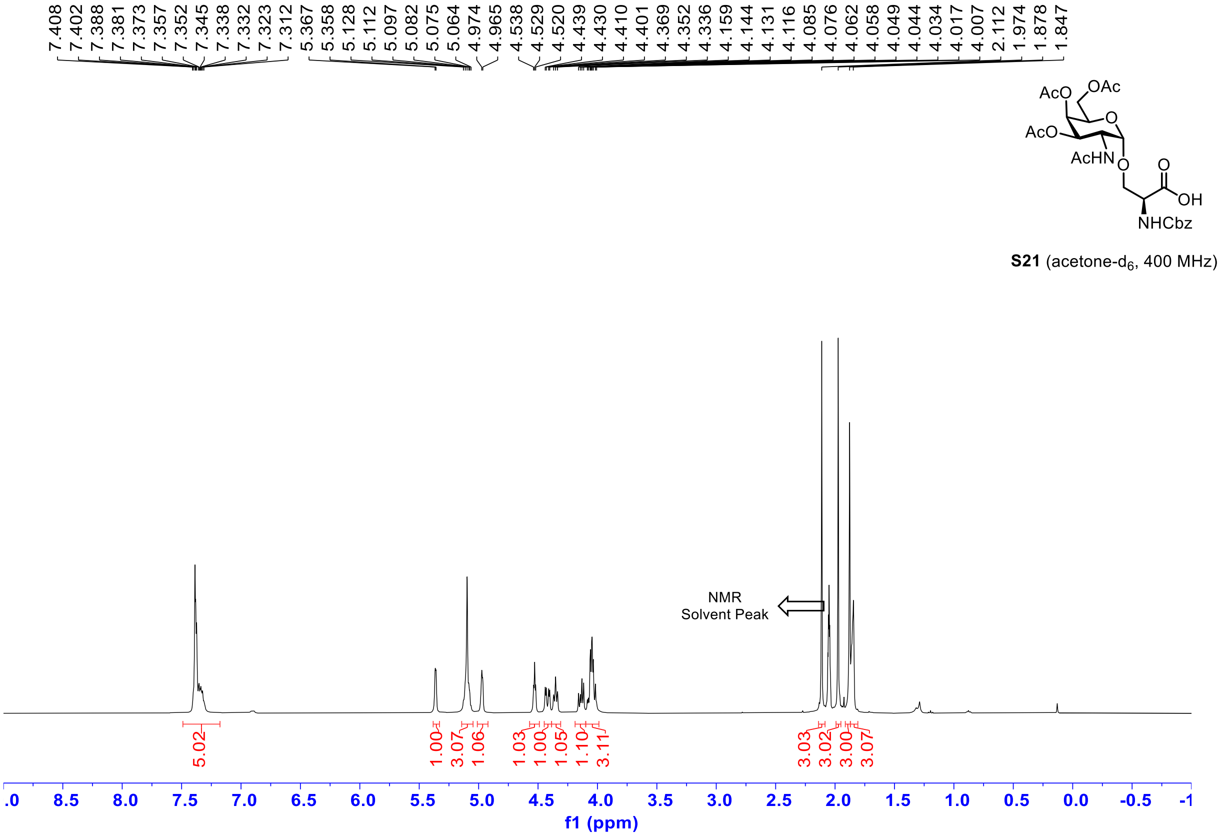
7.408 7.402 7.388 7.381 7.373 7.357 7.352 7.345 7.338 7.332 7.323 7.312 5.367 5.358 5.128 5.112 5.097 5.082 5.075 5.064 4.974 4.965 4.538 4.529 4.520 4.439 4.430 4.410 4.401 4.369 4.352 4.336 4.159 4.144 4.131 4.116 4.085 4.076 4.062 4.058 4.049 4.044 4.034 4.017 4.007 2.112 1.974 1.878 1.847
AcO
OAc
O
AcO
AcHN
O
O
OH
NHCbz
S21 (acetone-d6, 400 MHz)
NMR
Solvent Peak
5.02
1.00
3.07
1.06
1.03
1.00
1.05
1.10
3.11
3.03
3.02
3.00
3.07
f1 (ppm)

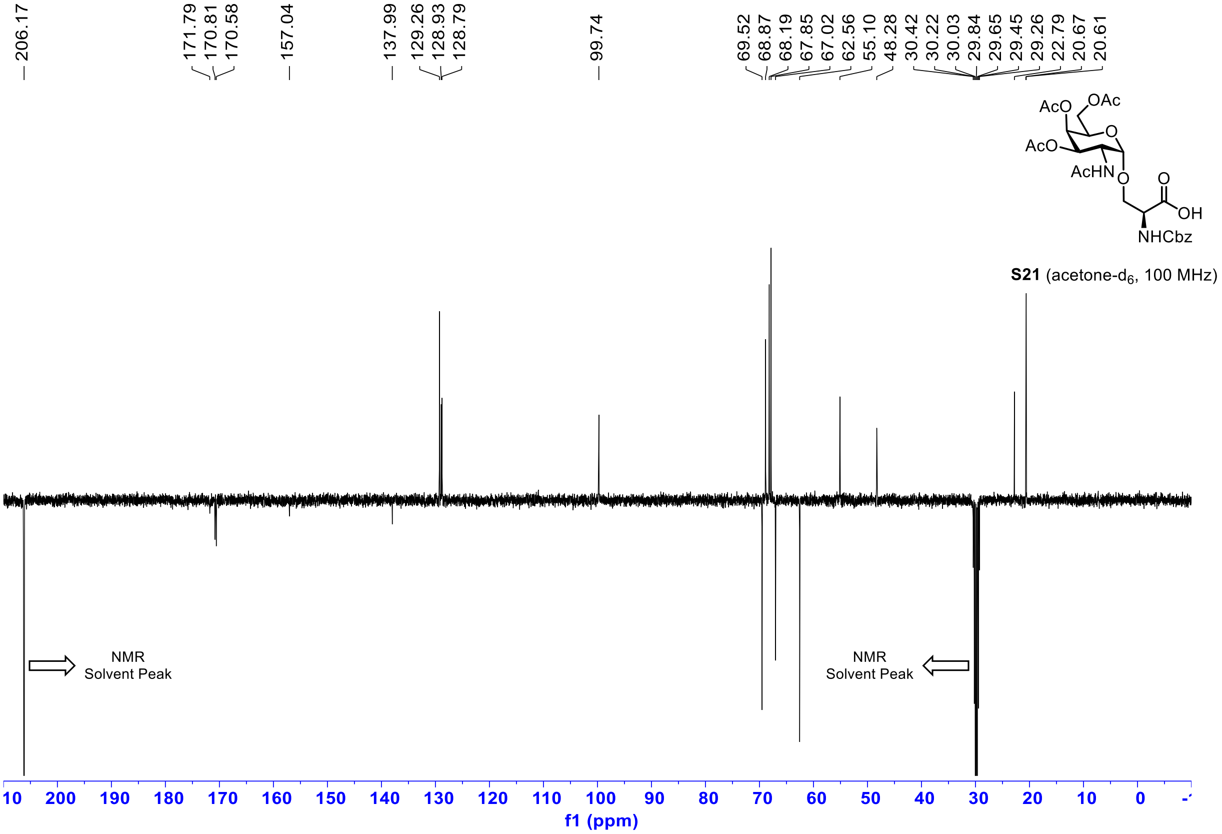
206.17 171.79 170.81 170.58 157.04 137.99 129.26 128.93 128.79 99.74 69.52 68.87 68.19 67.85 67.02 62.56 55.10 48.28 30.42 30.22 30.03 29.84 29.65 29.45 29.26 22.79 20.67 20.61
AcO
OAc
O
AcO
AcHN
O
O
OH
NHCbz
S21 (acetone-d6, 100 MHz)
NMR
Solvent Peak
NMR
Solvent Peak
f1 (ppm)
